# ASCL2 Maintains Stemness Phenotype through ATG9B and Sensitizes Gliomas to Autophagy Inhibitor

**DOI:** 10.1002/advs.202105938

**Published:** 2022-07-26

**Authors:** Li‐Hong Wang, Ye Yuan, Jiao Wang, Ying Luo, Yang Lan, Jia Ge, Lei Li, Feng Liu, Qing Deng, Ze‐Xuan Yan, Mei Liang, Sen Wei, Xin‐Dong Liu, Yan Wang, Yi‐Fang Ping, Yu Shi, Shi‐Cang Yu, Xia Zhang, You‐Hong Cui, Xiao‐Hong Yao, Hua Feng, Tao Luo, Xiu‐Wu Bian

**Affiliations:** ^1^ Institute of Pathology and Southwest Cancer Center Southwest Hospital Third Military Medical University (Army Medical University) and Key Laboratory of Tumor Immunopathology Ministry of Education of China Chongqing 400038 China; ^2^ Bio‐Bank of Southwest Hospital Third Military Medical University (Army Medical University) Chongqing 400038 China; ^3^ Department of Neurosurgery Southwest Hospital Third Military Medical University (Army Medical University) Chongqing 400038 China

**Keywords:** autophagy, adult diffuse gliomas, ATG9B, ASCL2, stemness, ROC‐325

## Abstract

Autophagy is a highly conserved process that is vital for tumor progression and treatment response. Although autophagy is proposed to maintain the stemness phenotype in adult diffuse glioma, the molecular basis of the link between autophagy and stemness is poorly understood, which makes it impossible to effectively screen for the population that will benefit from autophagy‐targeted treatment. Here, ATG9B as essential for self‐renewal capacity and tumor‐propagation potential is identified. Notably, ASCL2 transcriptionally regulates the expression of ATG9B to maintain stemness properties. The ASCL2‐ATG9B axis is an independent prognostic biomarker and indicator of autophagic activity. Furthermore, the highly effective blood–brain barrier (BBB)‐permeable autophagy inhibitor ROC‐325, which can significantly inhibit the progression of ASCL2‐ATG9B axis^High^ gliomas as a single agent is investigated. These data demonstrate that a new ASCL2‐ATG9B signaling axis is crucial for maintaining the stemness phenotype and tumor progression, revealing a potential autophagy inhibition strategy for adult diffuse gliomas.

## Graphical abstract

A novel ASCL2‐ATG9B axis for self‐renewing capacity and tumor‐propagating potential in adult diffuse gliomas is identified. Furthermore, high blood–brain barrier permeable and effective autophagic inhibitor ROC‐325, which can significantly inhibit the progression of ASCL2‐ATG9B axis^High^ gliomas as a single agent is screened. This study indicates a potential autophagy inhibition strategy for ASCL2‐ATG9B axis^High^ glioma patients.

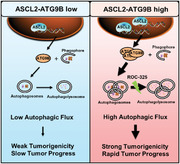

## 1 Introduction

Adult diffuse gliomas are the most common malignant primary brain tumors that exhibit extensive infiltration, and the most malignant and frequent tumor is glioblastoma (GBM), with a 5‐year overall relative survival of only 6.8%.^[^ ^1^ ^]^ Accumulating evidence suggests that glioma stem‐like cells (GSCs) play an indispensable role in the progression of glioma. GSCs exhibit self‐renewal capacity, tumor‐propagation potential, and expression of stemness genes, which account for gliomagenesis, invasion, recurrence, and therapeutic resistance.^[^ ^2^ ^]^ Recently, based on scRNA‐seq and in vivo experiments, GSCs have been considered to be plastic cellular phenotypes rather than discrete cell types.^[^ ^3^ ^]^ Therefore, individual cell surface markers, such as CD133, CD15, and CD44, are far from comprehensive and often lack specificity to depict the plasticity of GSCs.^[^ ^4^ ^]^ Instead, the intrinsic functional hierarchy in glioma cells endows them with an ideal biomarker fulfilling necessary criteria to characterize the stemness phenotype. To this end, revealing the biological process via which stemness properties are maintained is vital for glioma treatment and highlights this process as a promising potential therapeutic target.^[^ ^5^ ^]^

Macroautophagy (referred to as autophagy) is an evolutionarily conserved biological process that includes the formation of double‐membrane vesicles for engulfing cellular proteins and organelles for delivery to the lysosome, wherein cargos are eventually degraded and released to the cytoplainsm.^[^ ^6^ ^]^ As inhibition of autophagy can suppress the growth of established tumors and improve therapeutic effects, many autophagy inhibitors have been developed for cancer treatment, and hydroxychloroquine (HCQ) was repurposed in extensive clinical trials.^[^ ^7^ ^]^ Recent research has revealed that autophagy maintains glioma stem‐like cell traits to sustain tumorigenesis and resist therapy,^[^ ^8^, ^9^ ^]^ which indicates that targeting autophagy to inhibit the stemness phenotype is a promising treatment strategy. However, no significant improvement in overall survival was reported in patients treated with HCQ combined with chemoradiotherapy compared to patients treated with chemoradiotherapy alone.^[^ ^10^ ^]^ One essential reason for the failure of clinical trials is that the upstream molecular mechanism of autophagy in stemness maintenance remains largely unknown. As a result, it is impossible to screen for patients who might benefit from autophagy‐targeted therapy.

To investigate the core molecular mechanism of autophagy in sustaining stemness properties, we screened autophagy‐related genes preferentially expressed in GSCs. We identified ATG9B as a potential autophagy‐related molecule for regulating the stemness phenotype. Knockdown of ATG9B inhibited the stemness phenotype in vitro and in vivo. Through multidimensional database analysis and experimental verification, we identified the stemness transcription factor ASCL2, which upregulated ATG9B to enhance autophagy and maintain stemness. Furthermore, a high level of expression of components of the ASCL2‐ATG9B axis is an unfavorable marker for glioma patients. Finally, we identified ROC‐325 as a single agent for the treatment of glioma patients with high expression of components of the ASCL2‐ATG9B axis. Our study revealed a novel mechanism of the ASCL2‐ATG9B axis in stemness maintenance and autophagy regulation in adult diffuse gliomas and demonstrated that the ASCL2‐ATG9B axis is a potential criterion for the blood–brain barrier (BBB)‐permeable autophagy inhibitor ROC‐325.

## 2 Results

### 2.1 ATG9B is Correlated with Stemness and Poor Prognosis in Adult Diffuse Gliomas

**Figure 1:**
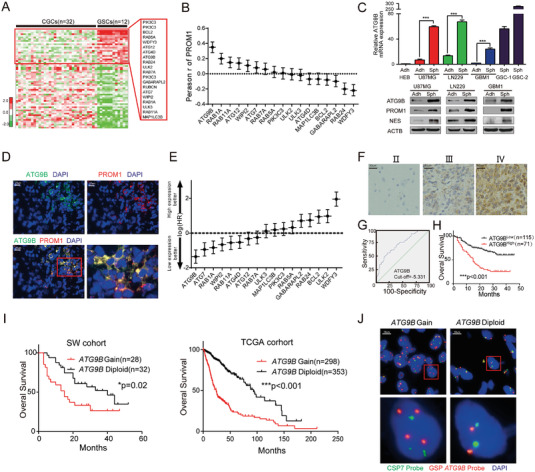
ATG9B is correlated with stemness and poor prognosis in gliomas. A) Expression heatmap of core autophagy‐related genes in GSCs (*n* = 12) relative to CGCs (*n* = 32) from GEO profiles (GDS3885). Candidate genes (*P* < 0.01) enriched in GSCs are enlarged in the red rectangle. The heatmap was visualized using Cluster/Java Treeview. B) Correlation analysis of the expression of candidate autophagy‐related genes and PROM1 in the TCGA LGGGBM dataset. *n* = 669, *P* < 0.001, Pearson's *r* test. C) The mRNA expression of ATG9B and protein expression of the indicated genes in adherent cells/tumorspheres of 2 glioma cell lines (U87MG, LN229) and 1 primary glioma cell line (GBM1). HEB cell line as negative control and primary glioma stem cells as positive control (GSC‐1 and GSC‐2). D) Coimmunofluorescence staining of ATG9B (in green) and a glioma stemness marker PROM1 (in red) in human GBM samples. The scale bar represents 20 µm. The magnified image is shown. E) The hazard ratios of candidate autophagy‐related genes in patients in the TCGA LGGGBM dataset (http://gliovis.bioinfo.cnio.es/); the cutoff value was the median expression level. F) Representative images of ATG9B immunohistochemistry in glioma tissues from 338 glioma patients from Southwest Hospital. Magnified images are shown. G) The cutoff value of the IOD of ATG9B in (F) was determined by ROC analysis. Patients with higher ATG9B expression were classified as the ATG9B^High^ group, and those with lower ATG9B expression were classified as the ATG9B^Low^ group. H) Kaplan–Meier curves of the overall survival rate of glioma patients from Southwest Hospital with high ATG9B expression (ATG9B^High^) versus low ATG9B expression (ATG9B^Low^). I) Overall survival analysis of glioma patients with *ATG9B* gain versus *ATG9B* diploid from the SW cohort and TCGA cohort. J) Representative images of fluorescence in situ hybridization (FISH) of samples with *ATG9B* gain and *ATG9B* diploid. Red dots indicate *ATG9B*, and green dots indicate chromosome 7. *p < 0.05, **p < 0.01, ***p < 0.001.

To investigate the function of autophagy in the maintenance of stemness in gliomas, we screened the expression of core autophagy genes in microarray profiles with GSCs (*n* = 12) and conventional glioma cell lines (CGCs) (*n* = 32).^[^ ^11^ ^]^ We identified 17 genes whose expression were elevated in GSCs relative to CGCs (*p* < 0.01) (**Figure** **1**A) and analyzed their correlation with the glioma stemness marker PROM1 in the TCGA LGGGBM dataset (Figure 1B). The linear correlation coefficient (*r*) of ATG9B was highest, which indicated the coexpression of ATG9B and PROM1 (Figure 1B). The correlation was verified in lower grade gliomas (LGG) and glioblastomas (GBM) in TCGA and CGGA datasets (Figure S1A, Supporting Information). ATG9 is an indispensable integral membrane protein that nucleates vesicles during autophagy initiation.^[^ ^12^ ^]^ In yeast, Atg9 proteoliposomes first recruit the phosphatidylinositol 3‐phosphate kinase complex, followed by Atg21, the Atg2‐Atg18 lipid transfer complex, and the E3‐like Atg12‐Atg5‐Atg16 complex, which promote Atg8 lipidation.^[^ ^13^ ^]^ The ATG9 family consists of two members: ATG9A and ATG9B. Knockdown of ATG9B reduces autophagosome formation under basal and induced autophagic process.^[^ ^14^, ^15^ ^]^ Although ATG9B participates in the prevention of hepatocarcinogenesis,^[^ ^16^ ^]^ it is also involved in tumor growth and poorer prognosis,^[^ ^17^ ^]^ which is consistent with the fact that autophagy plays different roles in tumorigenesis and tumor progression.^[^ ^18^ ^]^ ATG9B is reported to be involved in temozolomide resistance in glioblastoma,^[^ ^15^ ^]^ which indicates that ATG9B may be involved in the progression of gliomas; however, the function and mechanism of maintaining the stemness of ATG9B in adult diffuse gliomas are still largely unknown. We first studied the correlation of ATG9B expression with glioma stemness markers. In two gliomas datasets, ATG9B was significantly correlated with multiple glioma stemness markers (Figure S1B, Supporting Information). ATG9B expression was elevated in tumorspheres compared to adherent cells in mRNA and protein level (Figure 1C; and Figure S1C, Supporting Information). The correlation between ATG9B and PROM1 expression was also consistent with coimmunostaining of frozen glioma sections (Figure 1D). We analyzed hazard ratios (HRs) when patients with glioma (TCGA LGGGBM datasets in Gliovis) were divided into groups with expression above and below the median for the indicated autophagy genes. The HR was lowest when patients were grouped by ATG9B expression (Figure 1E). Furthermore, we measured ATG9B expression in 338 glioma samples from our hospital (SW cohort) with IHC (Table S1, Supporting Information) and downloaded the mRNA expression of ATG9B in gliomas (TCGA cohort) in the TCGA LGGGBM dataset (Table S2, Supporting Information). ATG9B expression increased in glioma samples according to the WHO grade (Figure 1F). In 186 patients with survival information in our cohort and patients in the TCGA cohort, ROC analysis was performed to determine the cutoff value (Figure 1G; and Figure S1D, Supporting Information), and overall survival analysis confirmed the significant correlation between higher expression of ATG9B and poorer OS (Figure 1H; and Figure S1E, Supporting Information). ATG9B expression, the clinicopathological and molecular characteristics of the SW cohort were summarized in Figure S1F, Supporting Information. We further performed survival analysis of patients with *ATG9B* gain/diploid in two cohorts. Overall survival was decreased in patients with *ATG9B* gain compared to those with ATG9B diploid (Figure 1I). An *ATG9B* probe was developed to confirm the status of *ATG9B* in glioma samples (Figure 1J). These data demonstrate that ATG9B is associated with an elevated stemness phenotype and poorer prognosis in gliomas.

### 2.2 Knockdown of ATG9B Impedes the Maintenance of the Stemness Phenotype of Glioma In Vitro and In Vivo

**Figure 2:**
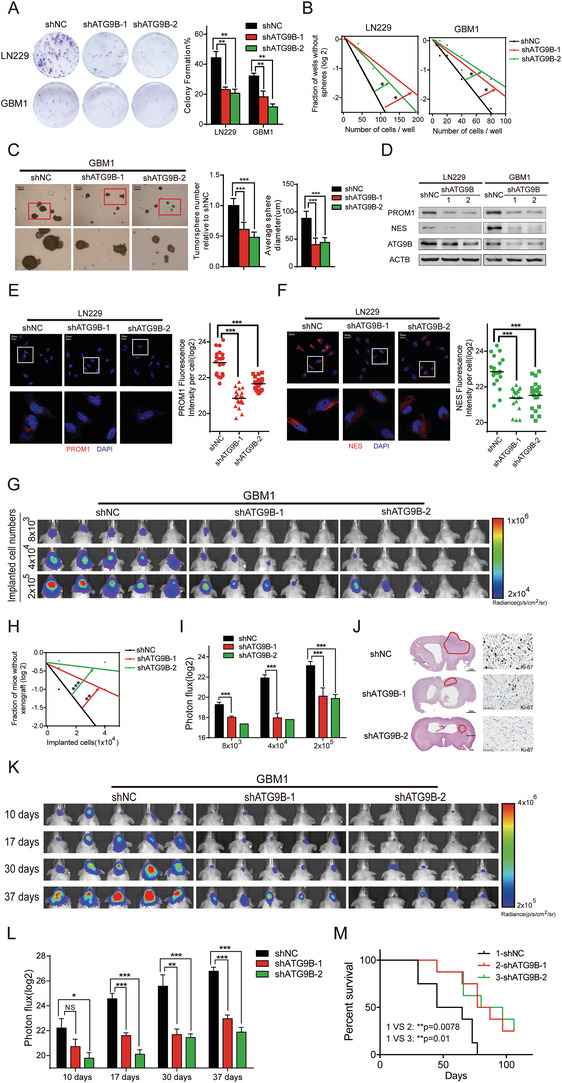
Knockdown of ATG9B impedes the maintenance of glioma stemness. A,B) The clone formation assay A) and in vitro limiting dilution assay B) of LN229/GBM1 cells expressing shNC/shATG9B‐1/shATG9B‐2. The data were collected on the 10th day after cell placement. C) Tumorsphere formation assay of GBM1 cells expressing shNC/shATG9B‐1/shATG9B‐2. Representative images of tumorspheres are shown (left panel). The numbers and diameters of tumorspheres were quantified (right panel). The data were collected on the 10th day after cell placement. D) The indicated molecules were measured by immunoblotting in LN229/GBM1 cells expressing shNC/shATG9B‐1/shATG9B‐2. E,F) Immunofluorescence of PROM1 E) and NES F) in LN229 cells expressing shNC/shATG9B‐1/shATG9B‐2 (left panel). Fluorescence intensity per cell was calculated (right panel). G) Representative bioluminescence images of intracranial xenografts derived from different numbers of GBM1 cells expressing shNC/shATG9B‐1/shATG9B‐2 *n* =8. H) The tumor formation incidence of **G** was determined through IVIS bioluminescence imaging, and the tumor formation efficiency was calculated by extreme limiting dilution analysis. I) Bioluminescence quantification of the intracranial xenografts in **G**. J) Representative HE staining of mouse brains from **G** derived from 2x10^5^ GBM1 cells. The tumor mass is outlined. Ki‐67 staining is shown in the right panel. K) Representative bioluminescence images of intracranial xenografts derived from 2x10^5^ GBM1 cells expressing shNC/shATG9B‐1/shATG9B‐2 at the indicated time points *n* = 8. L) Quantification of intracranial xenografts in **K**. M) Kaplan–Meier survival analysis of mice of **K**. *p < 0.05, **p < 0.01, ***p < 0.001.

To select suitable cell lines for functional experiments, ATG9B expression was measured in the human normal glial cell line (HEB), human glioma cell lines (T98G, U87MG and LN229), and primary human glioma cells (GBM1 and GBM2). We found that HEB expressed lower ATG9B and that the expression of ATG9B was higher in LN229 and GBM1 cells (Figure S2A, Supporting Information). As it was reported that ATG9B deficiency suppresses autophagy,^[^ ^14^ ^]^ we knocked down ATG9B with two independent short hairpin RNAs (shRNA, shATG9B‐1, and shATG9B‐2) and measured basal autophagy. Basal LC3 and autophagic flux were decreased relative to those of glioma cells expressing shNC (Figure S2B,C, Supporting Information). As ATG9B was elevated in gliomas, we investigated the function of ATG9B in maintaining stemness. Colony formation ability was inhibited by silencing ATG9B (**Figure** **2**A), and an in vitro limiting dilution assay demonstrated that knockdown of ATG9B impeded the self‐renewal ability of gliomas, which was confirmed by the reduced number and size of tumorspheres derived from glioma cells expressing shATG9B relative to those expressing shNC (Figure 2B,C). Consistently, the expression of the stemness markers PROM1 and NES decreased as ATG9B was silenced (Figure 2D–F). These in vitro data implied that silencing ATG9B reduced the stemness of glioma. An in vivo limiting dilution assay was performed to further verify the stemness function of ATG9B. The intracranial tumor formation incidence indicated that glioma cells expressing shATG9B initiated tumors at a lower frequency than those expressing shNC (Figure 2G–I). The expression of the proliferation marker Ki‐67 was significantly decreased in glioma cells expressing shATG9B (Figure 2J). Xenograft growth was also inhibited, and the survival date of mice with xenografts was prolonged when ATG9B was silenced (Figure 2K–M). With in vitro and in vivo data, we demonstrate that knockdown of ATG9B reduces the stemness properties of glioma.

### 2.3 ASCL2 Binds to the Promoter of ATG9B and Improves Basal Autophagy

**Figure 3:**
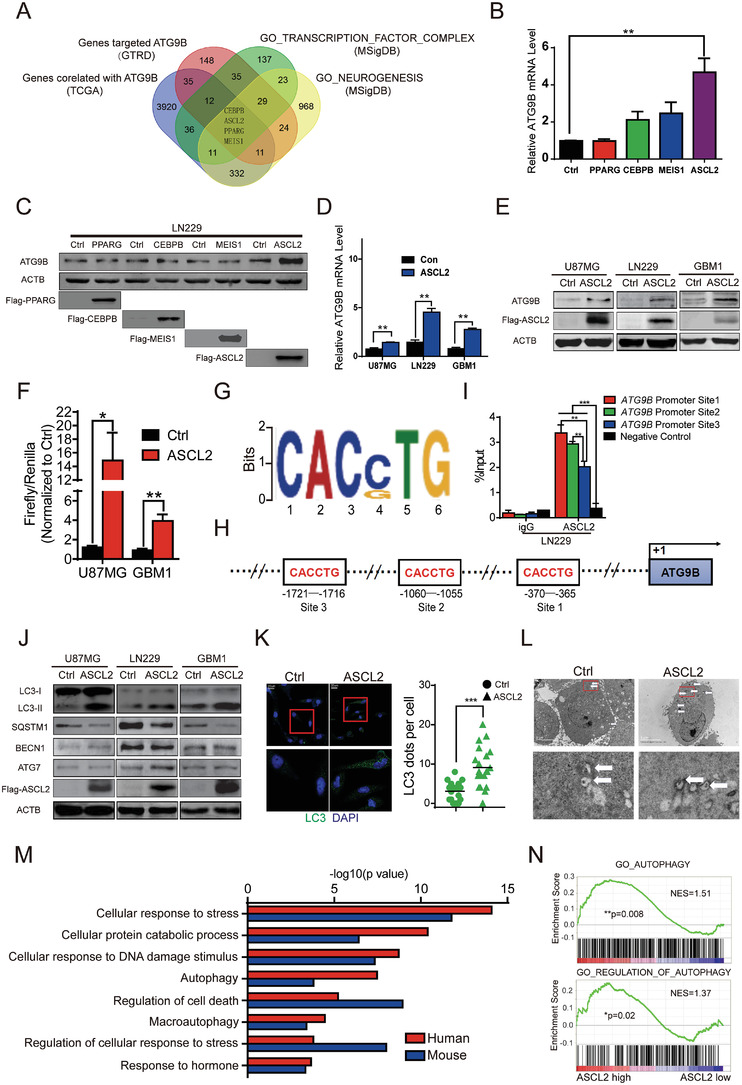
ASCL2 binds to the promoter of *ATG9B* and improves basal autophagy. A) Four candidate transcription factors were identified by multidimensional database analysis. B,C) ATG9B expression was measured with RT‐PCR B) and immunoblotting C) in LN229 cells transfected with the indicated transcription factors. D,E) The expression of ATG9B was detected with RT‐PCR D) and immunoblotting E) in the indicated glioma cells overexpressing Ctrl/ASCL2. F) A dual‐luciferase reporter assay was performed in U87MG/GBM1 cells transfected with Ctrl/ASCL2 plasmids and the *ATG9B* promoter reporter vector. G) The binding motifs of ASCL2 were analyzed with ChIP‐seq data from the GEO database (GSM1208812, GSM1208591). H) The three potential binding sites of ASLC2 in the promoter of *ATG9B* are shown. I) ChIP‐PCR assays were performed with primers targeting the binding sites of **I** in GBM1 cells. J) Autophagy‐related molecules were detected by immunoblotting in the indicated glioma cells overexpressing Ctrl/ASCL2. K) Representative images of LC3 staining in GBM1 cells overexpressing Ctrl/ASCL2, with magnified images (left panel). The number of LC3 dots per cell was quantified (right panel). Data are shown as scatter plots and means *n* = 20. L) Representative electron micrographs of autophagic vesicles or autophagosomes of GBM1 cells overexpressing Ctrl/ASCL2. Arrows denote autophagosomes. The scale bar represents 5 µm. Magnified image is shown. M) GO enrichment was performed for the genes that have an ASCL2 binding site in their promoter by using ChIP‐seq data from GEO (GSM1208812, GSM1208591, GSM1276937, GSM1276938). N) Autophagy was analyzed with a gene set enrichment assay (GSEA) according to ASCL2 expression in gliomas from the TCGA LGGGBM dataset. The normalized enrichment score (NES) and *p* value were calculated with GSEA software. *p < 0.05, **p < 0.01, ***p < 0.001.

Although ATG9B was a prognostic marker in univariate regression analysis, neither the expression status nor the copy number of ATG9B had independent prognostic value in the multivariable regression model (Table S3, Supporting Information). Furthermore, mRNA expression of genomic neighborhood of *ATG9B* grouped by gain/diploid status of the indicated genes was analyzed, and the elevation of gene expression was not consistent with the copy number status of each gene (Figure S3A, Supporting Information), which indicated that upstream regulators promoted *ATG9B* expression. Recent research implies that not only interactions in the cytoplasm but also transcriptional regulation in the nucleus affects the function of autophagy, which is mostly mediated by transcription factors.^[^ ^19^ ^]^ To identify the upstream regulator of ATG9B, 287 genes that could bind to the promoter of *ATG9B* were identified through ChIP‐seq data from Gene Transcription Regulation Database (GTRD).^[^ ^20^ ^]^ A list of genes whose expression correlated with ATG9B expression in the TCGA LGGGBM datasets, genesets containing transcription factor complexes in neurogenesis from MSigDB (Molecular Signatures Database) and genes from GTRD were intersected to identify 4 candidate transcription factors that might transcriptionally regulate ATG9B (**Figure** **3**A). We constructed overexpression plasmids to transfect glioma cells, and only elevated ASCL2 could significantly increase the mRNA and protein expression of ATG9B (Figure 3B,C), which was consistent in multiple glioma cells transfected with an ASCL2 overexpression lentivirus (Figure 3D,E). The promoter of *ATG9B* (2 kb upstream of the transcriptional start site) was inserted into the dual‐luciferase reporter plasmid, and glioma cells with ASCL2 overexpression exhibited higher transcriptional activity than those with Ctrl overexpression (Figure 3F). The binding motif of ASCL2 was illustrated with ChIP‐seq data, and the potential sites in the promoter of *ATG9B* were screened (Figure 3G,H). ChIP‐PCR confirmed that ASCL2 could bind to the promoter of *ATG9B* at three sites, which exhibited different efficiencies (Figure 3I). Via database screening and experimental testing, we identified that the transcription factor ASCL2 could bind to the promoter of *ATG9B* to transcriptionally regulate it.

ASCL2 (Achaete‐scute complex homolog 2, Mash2) is a member of the basic helix‐loop‐helix (BHLH) family of transcription factors that bind to e‐box (5′‐CANNTG‐3′) to activate transcription.^[^ ^21^ ^]^ As ASCL2 could transcriptionally regulate ATG9B, we assumed that ASCL2 could increase basal autophagy. As expected, overexpression of ASCL2 caused increased autophagic flux, as evidenced by conversion from LC3‐I to LC3‐II and degradation of SQSTM1 in multiple glioma cells, but did not significantly affect ATG7 and BECN1 (Figure 3J). LC3 fluorescent dots measured by fluorescence microscopy and autophagosomes visualized by transmission electron microscopy were increased in glioma cells overexpressing ASCL2 (Figure 3K,L; and Figure S3B,C, Supporting Information). Furthermore, after blockade of the autophagosomal‐lysosomal fusion process with HCQ, overexpression of ASCL2 resulted in increased accumulation of autophagosomes (Figure S3D, Supporting Information). We further performed pathway enrichment analysis with ASCL2 ChIP‐seq data for the promoters of genes from GSM1208591^[^ ^22^ ^]^ and GSM1276938,^[^ ^21^ ^]^ which indicated that ASCL2 may regulate the stress response and autophagy pathways (Figure 3M). Consistently, gene set enrichment analysis (GSEA) of the TCGA LGGGBM dataset proved that a high level of ASLC2 was correlated with autophagy‐related gene sets (Figure 3N). By measuring basal autophagy and performing ChIP‐seq and RNA‐seq data analysis, we conclude that overexpression of ASCL2 promotes basal autophagy.

### 2.4 ATG9B is Indispensable for ASCL2‐Mediated Autophagy and Stemness

**Figure 4:**
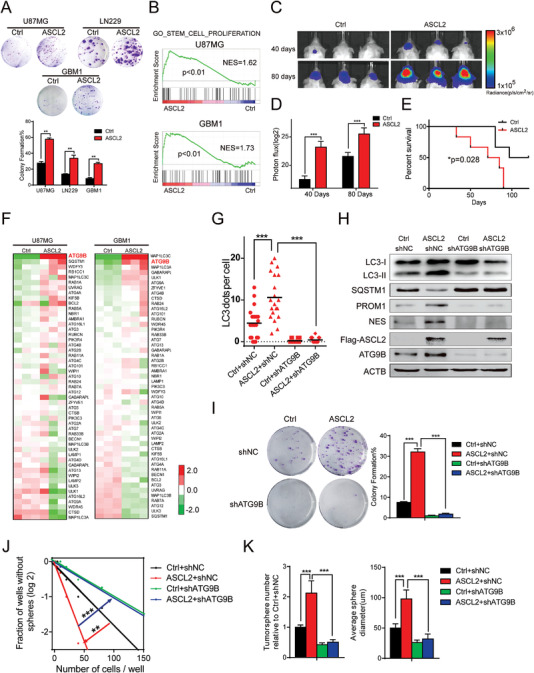
ATG9B is indispensable for ASCL2‐mediated autophagy and stemness. A) A clone formation assay was performed in the indicated glioma cells overexpressing Ctrl/ASCL2 (upper panel). Colony formation was calculated and is shown in the lower panel. The data were collected on the 7th day after cell placement. B) A stemness‐related gene set was analyzed by GSEA of Ctrl versus ASCL2 U87MG/GBM1 cells. C) Representative bioluminescence images of intracranial xenografts derived from GBM1 cells overexpressing Ctrl/ASCL2 *n* = 6. D) Bioluminescence quantification of the intracranial xenografts in (C). E) Kaplan–Meier survival analysis of mice bearing GBM1‐derived xenografts overexpressing Ctrl/ASCL2. F) Heatmap of autophagy‐related genes in U87MG/GBM1 cells overexpressing Ctrl/ASCL2. G,H) LC3 puncta per cell G) and the indicated molecules H) were measured in GBM1 cells expressing Ctrl/ASCL2 and shNC/shATG9B. Data are shown as scatter plots and means *n* = 20. I–K) The clone formation assay I), vitro limiting dilution assay J), and tumorsphere assay K) were performed in GBM1 cells expressing Ctrl/ASCL2 and shNC/shATG9B. The data were collected on the 7th day after cell placement. *p < 0.05, **p < 0.01, ***p < 0.001.

ASCL2 is first reported in neuronal precursors and played an essential role in extraembryonic development.^[^ ^23^, ^24^ ^]^ ASCL2 is associated with stemness maintenance of intestinal stem cells^[^ ^25^ ^]^ and is involved in tumor progression.^[^ ^26^ ^]^ However, the function of ASCL2 in glioma has been poorly illustrated. Stemness‐related experiments were performed. Enforced expression of ASCL2 in glioma cells improved colony formation and self‐renewal abilities (**Figure** **4**A; and Figure S4A,B, Supporting Information). With RNA‐seq data from U87MG/GBM1‐overexpressing Ctrl/ASCL2 cells, we analyzed the transcriptomes through GSEA, which revealed that stemness pathways were affected by ASCL2 overexpression (Figure 4B). mRNA and protein expression of stemness markers were elevated in glioma cells with ASCL2 relative to Ctrl (Figure S4C,D, Supporting Information). Furthermore, overexpression of ASCL2 in xenografts accelerated tumor growth, and mice inoculated with ASCL2 enforced‐expression cells survived shorter than those inoculated with control cells (Figure 4C–E). These results suggest that ASCL2 maintains stemness in glioma cells.

Although ATG9B is regulated by ASCL2, it is still unknown whether ATG9B is involved in ASCL2‐mediated autophagy and stemness. To demonstrate this hypothesis, we first analyzed the expression of core autophagy genes in glioma cells overexpressing ASCL2 relative to control cells, and ATG9B was significantly elevated in two glioma cell lines (Figure 4F). By analyzing markers of autophagy and autophagosomes, we found that knockdown of ATG9B in ASCL2‐overexpressing glioma cells significantly blocked ASCL2‐mediated upregulation of autophagy (Figure 4G,H). Furthermore, inhibition of ATG9B also impeded ASCL2‐mediated elevation of colony formation and self‐renewal abilities (Figure 4H–K), which implied that in addition to autophagy, ASCL2 also regulated the stemness phenotype in an ATG9B‐dependent manner, it was also verified in primary glioma stem cells (Figure S5A–C, Supporting Information). These data demonstrate that ATG9B is essential for the maintenance of stemness and the upregulation of autophagy by ASCL2.

Wnt/*β*‐catenin is reported as upstream of ASCL2 in intestinal crypts, gastric carcinoma, and colorectal cancer.^[^ ^26^, ^27^, ^28^ ^]^ However, it is unknown whether wnt/*β*‐catenin regulates ASCL2 in gliomas. The correlation of ASCL2 and CTNNB1 (*β*‐catenin) was significant in TCGA gliomas dataset (Figure S5D, Supporting Information). Activation of wnt/*β*‐catenin signaling increased the mRNA and protein expression of ASCL2 and inhibition of wnt/*β*‐catenin signaling decreased them (Figure S5E–H, Supporting Information). These data indicate that ASCL2 is regulated by wnt/*β*‐catenin in gliomas.

### 2.5 The ASCL2‐ATG9B Axis is an Independent Prognostic Marker in Gliomas

**Figure 5:**
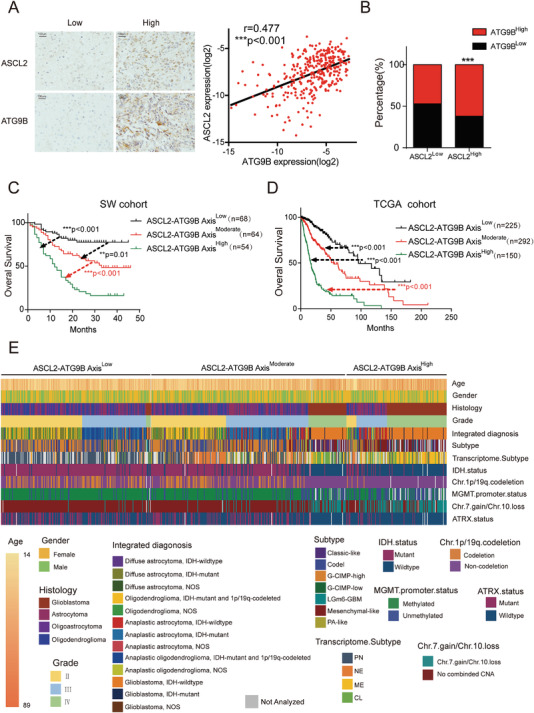
The ASCL2‐ATG9B axis is associated with poor prognosis in gliomas. A) Representative IHC of ASCL2 and ATG9B in glioma specimens (left panel). The scale bar represents 50 µm. Associations between the expression of ASCL2 and ATG9B assessed by linear regression and Pearson's *r* test in 338 glioma specimens (right panel). B) The relationship between ASCL2 and ATG9B mRNA expression in glioma on the basis of the TCGA cohort. C) Kaplan–Meier survival analysis of patients from the SW cohort divided into three groups (ASCL2‐ATG9B axis^Low^ indicates patients with low expression of ASCL2 and ATG9B; ASCL2‐ATG9B axis^High^ indicates patients with high expression of ASCL2 and ATG9B; and ASCL2‐ATG9B axis^Moderate^ indicates the remaining patients) *n* = 186. The cutoff value was defined by the ROC curve in Figure 1G; and Figure S5C (Supporting Information). D) Kaplan–Meier survival analysis of patients from the TCGA cohort divided into the three indicated groups *n* = 667. E) Heatmap showing the distribution of clinical features and genetic characteristics in glioma specimens from the TCGA cohort grouped by expression of components of the ASCL2‐ATG9B axis. ***p < 0.001.

To further investigate the clinical application value of ASCL2, we analyzed the expression of ASCL2 in the SW cohort and TCGA cohort. ASCL2 was elevated according to the WHO grade (Figure S6A,B, Supporting Information). IHC of ASCL2 was performed in 338 glioma specimens, ROC analysis was performed to determine the cutoff value, and Kaplan–Meier survival analysis indicated that patients with high ASCL2 expression exhibited poorer overall survival than those with low ASCL2 expression (Figure S6C, Supporting Information). This result was validated in the TCGA cohort (Figure S6D, Supporting Information). These results suggested that ASCL2 was associated with poor prognosis in gliomas. As the function and mechanism of the ASCL2‐ATG9B axis was revealed in gliomas, we further considered the relationship between ASCL2 and ATG9B in glioma samples. In the SW cohort, ASCL2 was positively correlated with ATG9B (**Figure** **5**A). Similar to protein expression, mRNA expression was also correlated between ASCL2 and ATG9B (Figure 5B). With cutoff values for ASCL2 and ATG9B expression, the patients were separated into three groups (ASCL2^Low^/ATG9B^Low^ as ASCL2‐ATG9B axis^Low^, ASCL2^High^/ATG9B^Low^ and ASCL2^Low^/ATG9B^High^ as ASCL2‐ATG9B axis^Moderate^, and ASCL2^High^/ATG9B^High^ as ASCL2‐ATG9B axis^High^). Regardless of the mRNA or protein level, the patients in the ASCL2‐ATG9B axis^High^ group exhibited poorer prognosis than the other 2 groups, which indicated that the ASCL2‐ATG9B axis could be a prognostic marker in gliomas (Figure 5C,D). Furthermore, we analyzed the relationship between the ASCL2‐ATG9B axis and clinicopathological/mutational characteristics. Relative to gliomas in the ASCL2‐ATG9B axis^Low^ group, gliomas with high expression of the ASCL2‐ATG9B axis exhibited more GBM and Chr.7.gain/Chr.10.loss, higher age and grade, fewer IDH mutations and 1p/19q codeletion (Figure 5E). To investigate whether the ASCL2‐ATG9B axis is an independent biomarker of prognosis, we performed Cox regression analysis. In the multivariable analysis, the ASCL2‐ATG9B axis (hazard ratio 1.28, 95% confidence interval 1.00–1.63) was independently associated with overall survival (**Table** **1**). Taken together, these results imply that the ASCL2‐ATG9B axis is positively associated with poorer survival and could be an independent prognostic marker in gliomas.

**Table 1:** Univariate and multivariable Cox regression analyses of factors associated with overall survival in glioma patients

Variable	Univariate analysis		Multivariable analysis	
	HR (95% CI)	*p*	HR (95% CI)	*p*
Age	5.68(4.20–7.68)	<0.01	1.79(1.26–2.55)	<0.01
Gender	0.93(0.70–1.23)	0.61	∖	∖
Grade	4.83(3.84–6.07)	<0.01	1.78(1.34–2.35)	<0.01
Histology	1.93(1.66–2.25)	<0.01	∖	∖
Integrated diagnosis	1.47(1.37–1.57)	<0.01	∖	∖
Subtype	1.48(1.37–1.60)	<0.01	0.69(0.57–0.85)	<0.01
IDH.status	0.10(0.08–0.14)	<0.01	0.08(0.04–0.17)	<0.01
Chr.1p/19q.codeletion	0.24(0.15–0.38)	<0.01	∖	∖
MGMT.promoter.status	0.30(0.22–0.40)	<0.01	∖	∖
Chr.7.gain/Chr.10.loss	8.72(6.33–12.02)	<0.01	∖	∖
ATRX.status	0.45(0.32–0.62)	<0.01	∖	∖
ASCL2‐ATG9B axis	2.66(2.17 –3.26)	<0.01	1.28(1.00–1.63)	<0.05

### 2.6 Targeting the ASCL2‐ATG9B Axis for the Treatment of Gliomas with Autophagy Inhibitors

**Figure 6:**
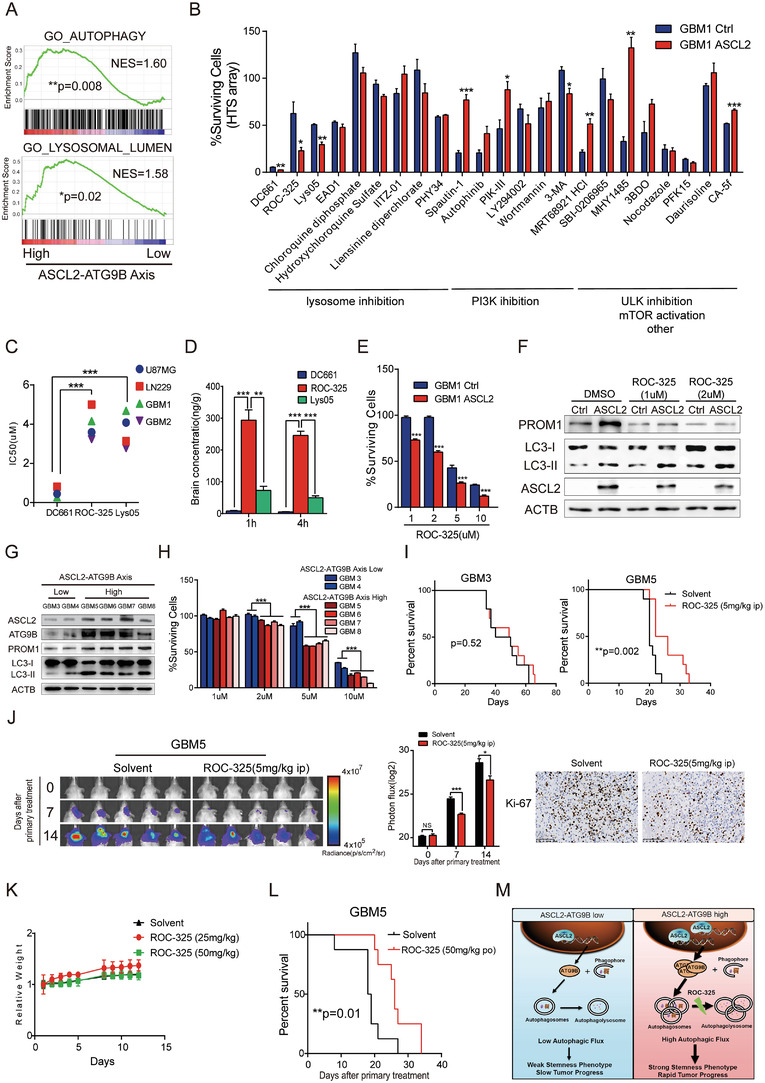
Targeting the ASCL2‐ATG9B axis for the treatment of gliomas with autophagy inhibitors. A) Autophagy‐related gene sets were analyzed with GSEA according to the ASCL2‐ATG9B axis of gliomas from the TCGA LGGGBM dataset. B) The percentage of surviving GBM1 cells overexpressing Ctrl/ASCL2 treated with the indicated autophagy inhibitors by HTS array. C) IC50 graphs for DC661, ROC‐325, and Lys05 in the indicated glioma cell lines and primary glioma cells generated from 72 h MTT assays. D) The ratio of compounds in the brain and plasma at different time points after treatment with 10 mg kg^−1^ DC661, ROC‐325 and Lys05 via intraperitoneal injection (i.p.). E) The percentage of surviving GBM1 cells overexpressing Ctrl/ASCL2 treated with the indicated concentration of ROC‐325 for 72 h. F) The indicated molecules were detected by immunoblotting in GBM1 cells overexpressing Ctrl/ASCL2 treated with 1 and 2 µm ROC‐325 for 48 h. G) The indicated molecules were measured in primary glioma cells with different expression levels of components of the ASCL2‐ATG9B axis. H) The percentage of surviving primary glioma cells treated with the indicated concentration of ROC‐325 for 72 h. I) Kaplan–Meier survival analysis of mice bearing GBM3‐ and GBM5‐derived xenografts treated with solvent/ROC‐325 (5 mg kg^−1^, 5 days a week, ip) *n* = 10. J) Representative bioluminescence images and quantification of intracranial xenografts derived from GBM5 cells treated with solvent/ROC‐325 (5 mg kg^−1^, 5 days a week, ip). Ki‐67 staining Bioluminescence of intracranial xenografts in the right panel. K) C57BL/6 mice were treated with solvent/ROC‐325 (25 or 50 mg kg^−1^) for 5 days every week for 2 weeks by oral administration. Relative weight was calculated at the indicated time points *n* = 3. L) Kaplan–Meier survival analysis of mice bearing GBM5‐derived xenografts treated with solvent/ROC‐325 (50 mg kg^−1^, 5 days a week, po) *n* = 8. M) Overview of the results. In glioma with high expression of components of the ASCL2‐ATG9B axis, more ASCL2 binds to the promoter of *ATG9B*, increasing the expression of ATG9B and autophagic activity and leading to a strong stemness phenotype and tumor progression. The oral autophagy inhibitor ROC‐325 attenuated autophagic flux to slow tumor growth. *p < 0.05, **p < 0.01, ***p < 0.001.

Autophagy is potential target of glioma treatment. However, inhibition of autophagy did not significantly improve the OS of glioma patients in a clinical trial. Screening for patients who will benefit from autophagy inhibitors is a good strategy. We performed preclinical studies to validate the ASCL2‐ATG9B axis as a predictive biomarker for the efficacy of autophagy inhibitors. Patients with ASCL2‐ATG9B axis^High^ possessed high autophagic and lysosomal activity, thereby suggesting higher susceptibility to autophagy inhibitors (**Figure** **6**A). To test this hypothesis, we assessed the tumor inhibition effect of an autophagy inhibitor library by performing high‐throughput screening (HTS). These results showed that DC661, ROC‐325, and Lys05 significantly inhibited the proliferation of ASCL2‐overexpressing glioma cells (Figure 6B). Although DC661 exhibited the lowest IC50 in glioma cells, ROC‐325 exhibited the highest permeability through the blood–brain barrier (Figure 6C,D). ROC‐325 is a novel orally available inhibitor of lysosome‐mediated autophagy that exhibits anticancer activity as a single agent.^[^ ^29^, ^30^ ^]^ ROC‐325 inhibited the proliferation and expression of stemness markers in ASCL2‐overexpressing glioma cells in a dose‐dependent manner (Figure 6E,F), and we confirmed the proliferative inhibition ability of primary glioma cells with an ASCL2‐ATG9B Axis^High^ phenotype (Figure 6G,H). ROC‐325 significantly extended the survival time of mice bearing ASCL2‐ATG9B Axis^High^ glioma cells but not mice bearing ASCL2‐ATG9B Axis^Low^ glioma cells (Figure 6I). Intraperitoneal administration of ROC‐325 restrained malignant hyperplasia of glioma cells and the proliferation index (Figure 6J; and Figure S7A, Supporting Information). To further address the application prospects of oral administration, we treated a xenograft model intragastrically with ROC‐325. The results showed that intragastric administration of ROC‐325 also prolonged the survival time of mice bearing ASCL2‐ATG9B Axis^High^ glioma cells without significant toxicity (Figure 6K,L; and Figure S7B, Supporting Information). These preclinical data imply that patients with ASCL2‐ATG9B Axis^High^ gliomas could benefit from autophagy inhibitor therapy. Finally, we measured synergistic effect of ROC‐325 and temozolomide (TMZ) in vitro and in vivo. The synergy scores were presented in both a matrix format and surface plots, the data in red squares in the matrix plots was furtherly analyzed by combination index. As expected, ROC‐325 and TMZ exhibited synergistic effect in gliomas (Figure S8A,B, Supporting Information), ROC‐325 also inhibited PROM1 expression by blocked TMZ‐induced autophagic flux (Figure S8C, Supporting Information). Furthermore, combination of ROC‐325 and TMZ significantly inhibited of tumor growth of intracranial xenografts compared to temozolomide alone (Figure S8D, Supporting Information). These data indicate that ROC‐325 could sensitize gliomas to TMZ treatment.

## 3 Discussion

Therapeutics specifically targeting cancer stem cells pave the way for novel strategies to prevent cancer replacement and prolong survival. In recent years, experimental observations have suggested that autophagy inhibition represents a promising target for counteracting cancer stem cell aggressiveness, thereby sensitizing cancer cells to therapy.^[^ ^31^, ^32^ ^]^ However, the function of autophagy in the stemness phenotype is paradoxical, as autophagy plays a dual role in cancer.^[^ ^6^ ^]^ On the one hand, autophagy sustains the self‐renewal and therapy resistance of cancer stem cells;^[^ ^33^, ^34^ ^]^ on the other hand, loss of autophagy impedes stem cell differentiation and increases tumorigenicity.^[^ ^35^ ^]^ In glioma stem cells, diverse inducer‐mediated autophagy plays distinct roles.^[^ ^8^, ^36^ ^]^ Thus, obtaining insights into the core regulatory factors and molecular mechanisms of cancer stem cells in which autophagy exploits its function is imperative for identifying more prudent anticancer strategies. Nevertheless, the core basal autophagic genes maintaining the stemness phenotype are unknown. In our study, we rigorously characterized the core autophagy‐related genes elevated in GSCs and tested their correlation with stemness markers in clinical samples. ATG9B, which was vital in phagophore formation, was appraised as a potential core gene for autophagy in maintaining the stemness of gliomas. We investigated whether *ATG9B* gain or high expression was correlated with poor overall survival of glioma patients. Functionally, we found that disruption of ATG9B inhibited the self‐renewal and neurosphere formation ability in vitro and in vivo. These results reveal that ATG9B is essential for sustaining the stemness phenotype.

Autophagic regulation is commonly seen as an interaction network in the cytoplasm. However, emerging research has uncovered that autophagy is vulnerable to transcriptional regulation in the nucleus.^[^ ^19^ ^]^ Although autophagy was proven to be indispensable in stem cells, the upstream regulatory mechanism needs further research. In the present study, it is worth noting that ATG9B did not reach statistical significance in predicting the prognosis of glioma patients in a multivariable regression model, which indicated that the upstream regulator of ATG9B is an influencing factor and endowed it with prognostic value. To further refine the upstream targets, we identified four functional transcription factors binding to the promoter of ATG9B, and ASCL2 was screened by experimental validation. Interestingly, it was reported that ASCL2 was essential for sustaining stemness properties in embryonic and adult stem cells.^[^ ^25^ ^]^ However, its function in gliomas has been poorly investigated. Our study unveiled that ASCL2 improved basal autophagy to maintain stemness through transcriptional regulation of ATG9B and that elevated ASCL2 promoted glioma progression in vivo. We further proved that ASCL2 was positively correlated with ATG9B and that the ASCL2‐ATG9B axis could be an independent prognostic factor, which indicated that the ASCL2‐ATG9B axis might be a functional unit in regulating glioma progression. The findings demonstrate that ASCL2‐dependent transcriptional regulation of autophagy is a crucial nuclear event for maintaining stemness and we identify a new ASCL2‐ATG9B signaling axis, which reveals the connection between autophagy and stemness.

Extensive preclinical evidence has demonstrated that genetic or pharmacological inhibition of autophagy improves clinical outcomes in cancer patients.^[^ ^7^ ^]^ However, pharmacological inhibition of autophagy with hydroxychloroquine in gliomas is disappointing.^[^ ^10^ ^]^ There are several reasons for this failure, including a lack of indications for autophagy inhibitors, low permeability of the BBB and drug toxicity. Screening molecular indications and identifying BBB‐permeable autophagy inhibitors are indispensable for improving the effectiveness of autophagy‐targeted therapy. In our study, we demonstrated that combined ASCL2 and ATG9B expression could be an indicator of autophagic activity. To track the therapeutic potential of the ASCL2‐ATG9B axis in more detail, we identified ROC‐325, an autophagy inhibitor with high BBB permeability and effectivity, as a single agent for the treatment of ASCL2‐ATG9B Axis^High^ glioma cells. As expected, validation studies showed that ROC‐325 significantly decreased the progression of glioma cells with high ASCL2‐ATG9B axis activity, indicating that ROC‐325 has great potential to improve the survival of glioma patients with high expression of the ASCL2‐ATG9B signaling axis (Figure 6M). Our work not only reveals the potential indication for autophagy‐targeted therapy but also discovers autophagy inhibitors with high BBB permeability and good efficacy for treating adult diffuse gliomas.

## 4 Conclusion

Our research reveals that the connection between stemness and autophagy mediated by the ASCL2‐ATG9B axis is crucial for glioma progression. More importantly, the ASCL2‐ATG9B axis can serve as a promising prognostic marker and therapeutic target in gliomas.

## 5 Experimental Section

### Study Design

The aim of the study was to provide mechanistic insights between autophagy and stemness and pharmacological interference of ACL2‐ATG9B axis in adult diffuse gliomas. ASCL2/ATG9B expression by IHC staining and *ATG9B* amplification by FISH from SW cohort were characterized as routine diagnostic workup. Glioma datasets from Gene Expression Omnibus (GEO) and The Cancer Genome Atlas (TCGA) were combined and autophagy‐related gene 9B (ATG9B) was identified. Comprehensive molecular biology techniques were used to investigate the role of ATG9B in adult diffuse gliomas. Multidimensional database analysis and molecular and functional experiments were used to evaluate the relationship between ASCL2 and ATG9B. HTS and liquid chromatography‐mass spectrometry (LC‐MS) systems were used to identify potential autophagy inhibitors for gliomas. For in vitro studies, experiments included at least three biological replicates. For in vivo studies, experimental groups were composed of at least 6 animals each. The studies were not performed blindly.

### Autophagy‐Related Gene Analysis Using the GEO Database

GEO microarray data (GEO: GDS3885) were applied to determine the expression of autophagy‐related genes in 12 GSC lines and 32 CGCs.^[^ ^11^ ^]^ The list of core autophagy‐related genes was obtained from previous reports.^[^ ^37^ ^]^ Analyses were visualized using Java Treeview. Candidate genes enriched in GSCs were identified when the *p* < 0.01.

### Patient Samples and Glioma Databases

A total of 338 glioma samples were obtained from patients diagnosed at Southwest Hospital (Chongqing, P. R. China) from January 2015 through December 2017 (SW cohort). Specimens were fixed in a 4% buffered formaldehyde solution and then paraffin embedded. Histopathological diagnoses were independently made by two experienced pathologists according to the World Health Organization (WHO) classification (2016). The DNA of glioma samples was obtained with a TIANamp FFPE DNA Kit (DP331) from Tiangen BIotech Co. Ltd., IDH1/2 mutation analysis was performed by an ABI 3500DX Genetic Analyser with an IDH1/2 mutation detection kit from Yuanqi Bio Co. Ltd., MGMT methylation status was determined by SINOMD Co. Ltd., and 1p/19q codeletion testing was performed by fluorescence in situ hybridization (FISH) with probes from Guangzhou LBP Medicine and Technology Co. Ltd. The clinical and mutational information of these patients is summarized in Table S1, Supporting Information. A cohort of 669 glioma samples (TCGA cohort) including gene expression and clinical information from the TCGA_GBMLGG database was obtained from Gliovis (http://gliovis.bioinfo.cnio.es/). The clinical and mutational information of these patients is summarized in Table S2, Supporting Information. A cohort of 649 primary glioma samples (CCGA cohort) of gene expression was obtained from Gliovis (http://gliovis.bioinfo.cnio.es/). Protein extracts of human glioma specimens were ground with TGrinder (OSE‐Y50, TIANGEN) and prepared with RIPA lysis buffer (P0013B, Beyotime Biotechnology) according to the product manual. The clinicopathologic information of these patients is summarized in Table S4, Supporting Information. The experiment of patient samples was approved by the Ethics Committee of The First Affiliated Hospital of Army Medical University (KY2020288).

### Primers and Antibodies

Primers for ASCL2, ATG9B, PROM1, NES, and GAPDH were designed by Primer 3.0 or obtained from Primer Bank (https://pga.mgh.harvard.edu/primerbank/). The primers for suspicious ASCL2 binding sites at the promoter region of *ATG9B* were designed by Primer 3.0. All primers were listed in Table S5, Supporting Information. The antibodies used in this study are listed in Tables S6 and S7, Supporting Information.

### Immunohistochemistry (IHC) and Immunoreactivity Scores

Immunohistochemical staining and analyses were performed on human glioma tissues and tumor xenografts. The procedure was carried out based on the manufacturer's guidelines of the Dako REAL EnVision Detection System (DAKO). The IHC scoring method was performed as previously described.^[^ ^38^ ^]^ Three representative IHC images of each slide were captured with a microscope. The area sum and integrated optical density (IOD) sum of the positive site (brown) were measured in pixels by Image‐Pro Plus 5.0 software. The expression intensity was expressed by the mean value of IOD sum/area sum of 3 photographs for each slide. To ensure data comparability, the same parameter settings were kept for all photographs. The mean value of IOD is summarized in Table S1, Supporting Information.

### Cell Culture

HEB was maintained as described.^[^ ^39^ ^]^ U87MG and LN229 cells were obtained from the ATCC American Type Culture Collection. Primary glioblastoma cells (GBM1‐8) were established from the tumor specimens of patients with gliomas (Southwest Hospital). The clinicopathological information of GBM cells used in the study was listed in Table S8, Supporting Information. To isolate glioma cells from GBM tumors, tumor resection tissues were collected and cut into small pieces and then were isolated using the Papain Dissociation System (Worthington Biochemical, Lakewood, NJ) according to the manufacturer's instructions. The tumor tissues were further mechanically dissociated and the suspension was filtered with a 70 µm cell strainer (BD Biosciences, San Jose, CA) to remove tissue pieces. U87MG, LN229, HEB, and primary glioblastoma cells were maintained in Dulbecco's modified Eagle's medium (DMEM; Gibco) containing 10% FBS (Gibco). Gliomas stem cells(GSC‐1 and GSC‐2) were cultured in Neurobasal‐A medium (Gibco, 12349015) supplemented with B‐27 (Gibco, 17504‐044), 10 ng mL^−1^ EGF (Peprotech, #AF‐100‐15), 10 ng mL^−1^ bFGF (Peprotech, #AF‐100‐18B), 1 × MEM Non‐Essential Amino Acids (Gibco, 11140‐050), 1 × GlutaMAX (Gibco, 35035‐061), 1 × Sodium pyruvate (Gibco, 11360‐070) and 1 × Penicillin‐Streptomycin Solution (HyClone, SV30010). All the cells were incubated at 37 °C in a humidified incubator with 5% CO_2_/95% air.

### ATG9B Knockdown and ASCL2 Overexpression

To knock down ATG9B in glioma cells, two targeting shRNAs (named shATG9B) and a nontargeting scrambled RNA (named shNC) were constructed in lentivirus vectors. The shRNA sequence list was as follows: shATG9B‐1: GCGGCTGGTCCCGCCTGGCGCGCTT; shATG9B‐2: GCGCGCTTGCAGCTGCGCCACTTCA. Stably transfected cells were selected by a FACSAria II cell sorter (BD Biosciences). For ASCL2 overexpression in glioma cells, full‐length human ASCL2 (named ASCL2), and an empty vector (named Ctrl) were constructed with a lentivirus vector. Stably transfected cells were selected with 3 µg mL^−1^ puromycin in culture. All lentivirus particles were packaged by Hanbio Biotechnology.

ATG9B‐specific siRNA (GGCTCAACCTGCAATGACA) and a scrambled siRNA as a negative control (siNC) were purchased from RIBOBIO. Cells were seeded at 5 × 105 cells per well in six‐well flat bottom plates. After 2 h of incubation, 300 *µ*L Opti‐MEM reduced‐serum medium liquid (Gibico), 9 *µ*L lipofectamine RNAiMAX and siRNA (siNC or siATG9B) were mixed and incubated for 5 min. Then, the reagents were added to each well. The RNA interference assay was performed after 24 h of incubation.

### RNA Extraction and Quantitative PCR

Total RNA was isolated using RNAiso TRIzol reagent (Takara) and reverse transcribed with PrimeScript RT Master Mix (Takara) according to the manufacturer's instructions. Then, SYBR Fast qPCR Mix (Takara) in a Bio‐Rad CFX96 Real‐Time PCR Detection System (Bio‐Rad) was used for qRT‐PCR. qRT‐PCR was performed in triplicate, and the results were normalized against GAPDH.

### Western Blot Analysis

Glioma cells were washed twice with ice‐cold PBS and then lysed with protein extraction reagent (Beyotime Institute of Biotechnology) containing 1% protease inhibitors (Thermo Fisher Scientific). The lysate was centrifuged at 4 °C at 12 000 g for 15 min, and proteins were separated by SDS‐PAGE and transferred to polyvinylidene difluoride membranes (Millipore). After being blocked with 5% skim milk for 30 min, the membranes were incubated at 4 °C overnight with the indicated primary antibodies. After washing with PBST, the membranes were incubated with HRP‐conjugated secondary antibodies (Cell Signaling Technology) or IRDYR800CW goat antibodies for 1 h. Then, proteins were visualized with Super ECL Plus Western Blotting Substrate (BG0001, bgbiotech) and detected by a ChemiDocXRS system (Bio‐Rad) or Odyssey 2608 (LI‐COR).

### Clone Formation Assay

Cells were plated in 6‐well plates at a density of 300 cells per well. On the indicated day after cell placement, the clone number was counted, and the cloning efficiency was calculated.

After implantation, tumors were monitored by bioluminescence activity.

### Tumorsphere Formation Assay and In Vitro/Vivo Limiting Dilution Assay

Cells were plated in 24‐well plates at a density of 4000 cells per well in Neurobasal‐A medium supplemented with B‐27, 10 ng mL^−1^ EGF, 10 ng mL^−1^ bFGF, 1 × MEM Non‐Essential Amino Acids, 1 × GlutaMAX, 1 × Sodium pyruvate and 1 × Penicillin‐Streptomycin Solution, and tumorsphere numbers and sizes were calculated on the indicated day after cell placement.^[^ ^40^ ^]^ In the in vitro assay, cells were implanted into a 96‐well plate at a gradient of 5, 10, 20, 50, 100, or 200 cells per well in Neurobasal‐A medium as mentioned above, with 10 replicates for each gradient. The number of tumorspheres in each well was determined after incubation for the indicated days. In the in vivo assay, cells were implanted in the brains of six‐week‐old female SCID mice (Laboratory Animal Center, Third Military Medical University) at a density of 8 × 10^3^, 4 × 10^4^, or 2 × 10^5^ cells. After two weeks, the tumor volume was detected by an IVIS Spectrum system. The sphere/xenograft formation efficiency was calculated using extreme limiting dilution analysis (http://bioinf.wehi.edu.au/software/elda).

### Tumor Implantation

To determine the role of ATG9B disruption and ASCL2 overexpression on tumor growth, GBM1 cells (4 × 10^5^ cells per mouse) expressing shNT/shATG9B and GBM1 cells (2 × 10^5^ cells per mouse) expressing Ctrl/ASCL2 were implanted in the brains of female SCID mice. Tumor growth was monitored via bioluminescence imaging using an IVIS Spectrum system. When the mice could not actively eat and drink, they were sacrificed and considered deceased cases in the survival assay. All other tumor‐bearing mice were sacrificed at 18 weeks but classified as live cases. Fresh brain tissues were collected from sacrificed mice, fixed with formaldehyde, and embedded in paraffin for HE staining and IHC. Animal experiments were approved by the Laboratory Animal Welfare Ethics Committee of Third Military Medical University according to the Guide for the Care and Use of Laboratory Animals (AMUWEC20201467).

### Immunofluorescence (IF), Fluorescence Intensity Scores, and LC3 Dot Counting

Cells were seeded on a Cell Imaging Dish (Eppendorf) overnight growth, rinsed with PBS, and then fixed with 4% paraformaldehyde for 30 min. Cells were then rinsed with PBS three times and permeabilized with 0.5% Triton X‐100 for 20 min. Cells or slides with formalin‐fixed GBM tissues were blocked with 5% goat serum for 30 min at 37 °C. Primary antibodies were incubated overnight at 4 °C, secondary antibodies were incubated for 1 h at room temperature, and Hoechst was incubated for 30 min. Images were analyzed on an LSM780 confocal system (Zeiss, Jena, Germany). ZEN lite 2012 was used to analyze the expression of the indicated gene and LC3 dots. The fluorescence intensity of 20 random cells was calculated with a polygon tool and logarithmically transformed. The number of LC3 dots was counted in 20 randomly selected cells.

### Multidimensional Database Analysis

To predict the upstream transcription factor of ATG9B, multidimensional database analysis was performed. A total of 4361 genes that were significantly positively correlated with ATG9B (*r* > 0.2, adj. *p* < 0.01) were filtered in the RNA‐seq database of TCGA_GBMLGG (Gliovis). A total of 287 transcription factors that could bind to the promoter of ATG9B from *Homo sapiens* were identified according to the ChIP‐seq database (GTRD, http://gtrd.biouml.org/). The GO gene sets of TRANSCRIPTION_FACTOR_COMPLEX and NEUROGENESIS were obtained from the Molecular Signatures Database (http://software.broadinstitute.org/gsea/msigdb). A Venn diagram (http://bioinformatics.psb.ugent.be/webtools/Venn/) was generated to filter 4 genes that existed in all gene sets.

### ASCL2 ChIP‐seq Data Analysis

Rawdata of ASCL2 ChIP‐seq were downloaded in GEO (GSM1208812, GSM1208591, GSM1276937, GSM1276938). Format conversion proceeded with Fastq‐Dump, and quality control proceeded with fastQC. Uniquely mapped reads obtained with bowtie2 were used to call peaks with MACS14, and peaks were annotated with CEAS. DAVID was used to analyze the GO enrichment, and the binding motif was analyzed with MEME.

### Transfection and Dual‐Luciferase Reporter Assay

GBM cells seeded in 48‐well plates at a density of 1 × 10^5^ were cotransfected with 500 ng Ctrl/ASCL2 plasmids and 500 ng ATG9B promoter reporter vector (GENECHEM Shanghai, China) by using Lipofectamine 2000 (Invitrogen). After 24 h of incubation, luciferase activities were measured by using the Dual‐Luciferase Reporter Assay System (Promega, Madison, WI). The results are presented after normalization to the measured values of firefly luciferase.

### ChIP‐PCR

Chromatin immunoprecipitation (ChIP) was performed using the SimpleChIP Enzymatic Chromatin IP Kit (Cell Signaling) according to the manufacturer's protocol. Isotype IgG (#2985, Cell Signaling) and anti‐ASCL2 (MAB4418, MERCK) were used to immunoprecipitate chromatin fragments. The primer pairs that were designed according to 3 predicted binding regions of ATG9B are listed in Table S9, Supporting Information.

### RNA Sequencing

Total RNA was prepared from U87MG and GBM1 cells expressing ASCL2 (or Ctrl). RNA sequencing was performed with three replicates per group by BGI. Gene expression was quantified by RSEM.^[^ ^41^ ^]^ The functional classifications of the changed genes were described by Gene Set Enrichment Assay software (http://software.broadinstitute.org/gsea/index.jsp). Heatmaps were generated by Treeview.

### In Vitro Cell Viability Assay

The indicated cells were seeded in 96‐well plates (2000 cells per well) treated with/without autophagy inhibitorss (DC661, ROC‐325 and Lys05 from Selleck) at the indicated concentrations. Cell viability was determined at 72 h by cell counting kit‐8 (Dojindo Laboratories) according to the manufacturer's instructions. The IC50 values were determined by exposing cells to the indicated autophagy inhibitors/control (DMSO) at increasing concentrations (DC661, from 50 nm to 10 *µ* m; ROC‐325, from 0.5 to 20 *µ* m; and lys05, from 0.2 to 50 *µ* m). Cell viability was measured at 72 h after treatment.

### Brain Penetration Study

ICR mice were treated with autophagy inhibitors (DC661/ROC‐325/Lys05 at 10 mg kg^−1^) via ip administration. After 1 or 4 h, as indicated, the mice were sacrificed, and the brain and plasma were analyzed by LC‐MS/MS system (HPLC, Shimadzu LC30AD; Mass Spectrometer, LCMS8050‐2) according to the manufacturer's instructions.

### HTS

Cell viability assays in response to drug treatment were performed by plating 1000 cells in 96‐well plates (black clear bottom, Corning 3603). After overnight incubation, cells were treated with the indicated autophagy inhibitors for 72 h. Cells were then fixed with 4% paraformaldehyde solution and stained with DAPI to visualize the nuclei. Nine images from each well at 10x magnification were acquired using ImageXpress (Molecular Devices). For all analyses, nine images per well were collected from adjacent imaging sites on the same plane. Image segmentation to identify DAPI‐positive cells was performed by defining the minimum diameter of the object and intensity of the nuclear stain. The average signal intensity was extracted from nuclei, and a ratio was calculated and normalized to the medium control.

### Synergy Experiments

Experiments were analyzed for synergistic interactions by the HSA synergy and antagonism method using Combenefit (http://www.cruk.cam.ac.uk/research‐groups/jodrell‐group/combenefit). Combination index (CI) of Chou–Talalay analysis was calculated using the software CompuSyn (https://www.combosyn.com/index.html) to further validate the drug combinations. CI values offer quantitative definition for additive effect (CI = 1), synergism (CI < 1), and antagonism (CI >1).

### Statistical Analysis

All experiments were performed at least three times, and the results from representative experiments are presented as the mean ± s.e.m. or scatter plots and means. Unpaired Student's *t*‐test was applied to compare the statistical significance of differences. The correlation between indicated genes expression was analyzed using Pearson's *r* test. Patient survival was analyzed by the Kaplan–Meier method. These statistical analyses were performed using Prism software(GraphPad Software, San Diego, CA). ROC curve analysis and univariate/multivariable Cox regression analysis were performed in SPSS 18. All statistical tests were two‐tail. *p* < 0.05 was considered to indicate statistical significance (*p < 0.05, **p < 0.01, ***p < 0.001, NS: no significance).

## Conflict of Interest

The authors declare no conflict of interest.

## Supporting information

Supporting Information

## Data Availability

The data that support the findings of this study are available on request from the corresponding author. The data are not publicly available due to privacy or ethical restrictions.
