# Supplementary material for: ASCL2 Maintains Stemness Phenotype through ATG9B and Sensitizes Gliomas to Autophagy Inhibitor
Source: Adv Sci (Weinh). 2022 Jul 26;9(27):2105938. doi: 10.1002/advs.202105938 (PMC9507388; doi:10.1002/advs.202105938)
Supplement: Supplementary file 1 — Supporting Information [file ADVS-9-2105938-s001.pdf]

ADVANCED SCIENCE Open Access

Supporting Information

for *Adv. Sci.*, DOI 10.1002/advs.202105938

ASCL2 Maintains Stemness Phenotype through ATG9B and Sensitizes Gliomas to Autophagy Inhibitor

Li-Hong Wang, Ye Yuan, Jiao Wang, Ying Luo, Yang Lan, Jia Ge, Lei Li, Feng Liu, Qing Deng, Ze-Xuan Yan, Mei Liang, Sen Wei, Xin-Dong Liu, Yan Wang, Yi-Fang Ping, Yu Shi, Shi-Cang Yu, Xia Zhang, You-Hong Cui, Xiao-Hong Yao, Hua Feng, Tao Luo and Xiu-Wu Bian**

Supporting information

ASCL2 maintains stemness phenotype through ATG9B and sensitizes gliomas to autophagy inhibitor

Li-Hong Wang, Ye Yuan, Jiao Wang, Ying Luo, Yang Lan, Jia Ge, Lei Li, Feng Liu, Qing Deng, Ze-Xuan Yan, Mei Liang, Sen Wei, Xin-Dong Liu, Yan Wang, Yi-Fang Ping, Yu Shi, Shi-Cang Yu, Xia Zhang, You-Hong Cui, Xiao-Hong Yao, Hua Feng, Tao Luo[*] *and Xiu-Wu Bian*[*].

The file includes:

Figure S1. ATG9B is correlated with poor prognosis in gliomas.

Figure S2. Knock-down of ATG9B inhibits autophagy flux in gliomas.

Figure S3. The mRNA expression of genomic neighborhood of *ATG9B* was not consistent and ASCL2 overexpression activates autophagy.

Figure S4. Enforced expression of ASCL2 improves stemness phenotype.

Figure S5. ATG9B is indispensable for ASCL2-mediated in glioma stem cell and ASCL2 is regulated by CTNNB1 in gliomas.

Figure S6. ASCL2 is correlated with poor prognosis in gliomas.

Figure S7. ROC-325 inhibits proliferation of intracranial xenografts and has no severe systemic side effects.

Figure S8. ROC-325 sensitizes glioma cells to TMZ.

Table S1. The clinicopathologic mutational and survival information of patients with glioma in southwest (SW) hospital.

Table S2. The clinicopathologic mutational and survival information of patients with glioma in TCGA dataset.

Table S3. Univariate and multivariable Cox regression analyses of factors associated with overall survival in glioma patients(n=610).

Table S4. The clinicopathologic information of patients whose samples were used in figure S6.

Table S5. The primers of indicated genes.

Table S6. Primary antibodies information list.

Table S7. Second antibodies information list.

Table S8: The clinicopathologic information of primary glioma cells.

Table S9. The primer of 3 predicted binding region of ATG9B.

Figure S1

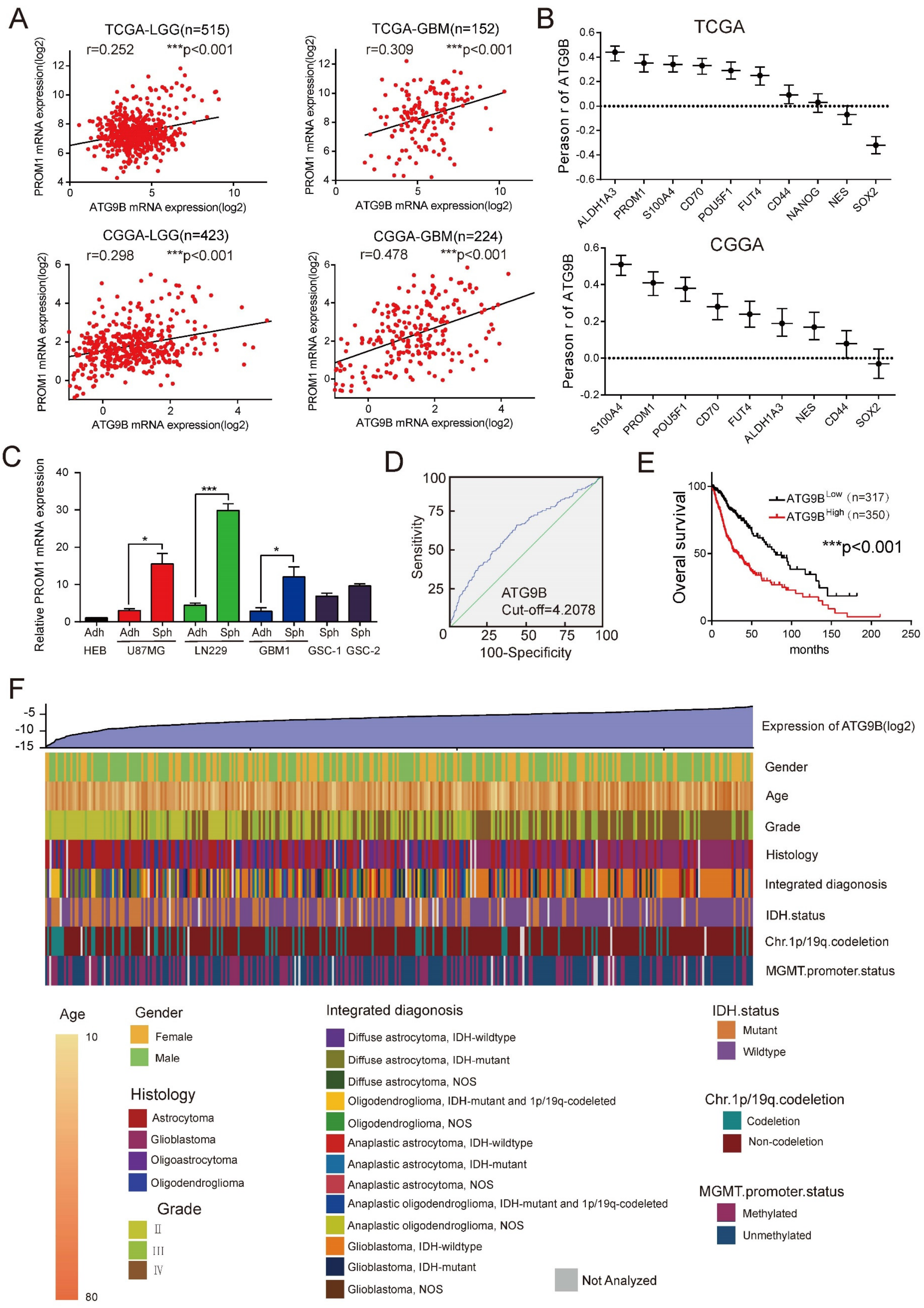

Figure S1. ATG9B is correlated with poor prognosis in gliomas. (A) Correlation analysis of the expression of ATG9B and stemness-related genes in the TCGA

(n=669) and CGGA(n=649) LGGGBM dataset, Pearson's r test. (B) Correlation analysis of the expression of ATG9B and PROM1 in the TCGA LGG(n=515)/GBM(n=152) and CGGA LGG(n=423)/GBM(n=224) datasets. Pearson's r test. (C) The mRNA expression of PROM1 in adherent cells/tumorspheres of 2 glioma cell lines (U87MG, LN229) and 1 primary glioma cell line (GBM1). HEB cell line as negative control and primary glioma stem cells as positive control (GSC-1 and GSC-2). (D, E) ROC analysis (D) and Kaplan–Meier survival analysis (E) were performed in patients with glioma in the TCGA database according to ATG9B mRNA expression. n=667, log-rank test. (F) Heatmap showing the distribution of clinical features and genetic characteristics in glioma specimens from Southwest Hospital. n=338.

Figure S2

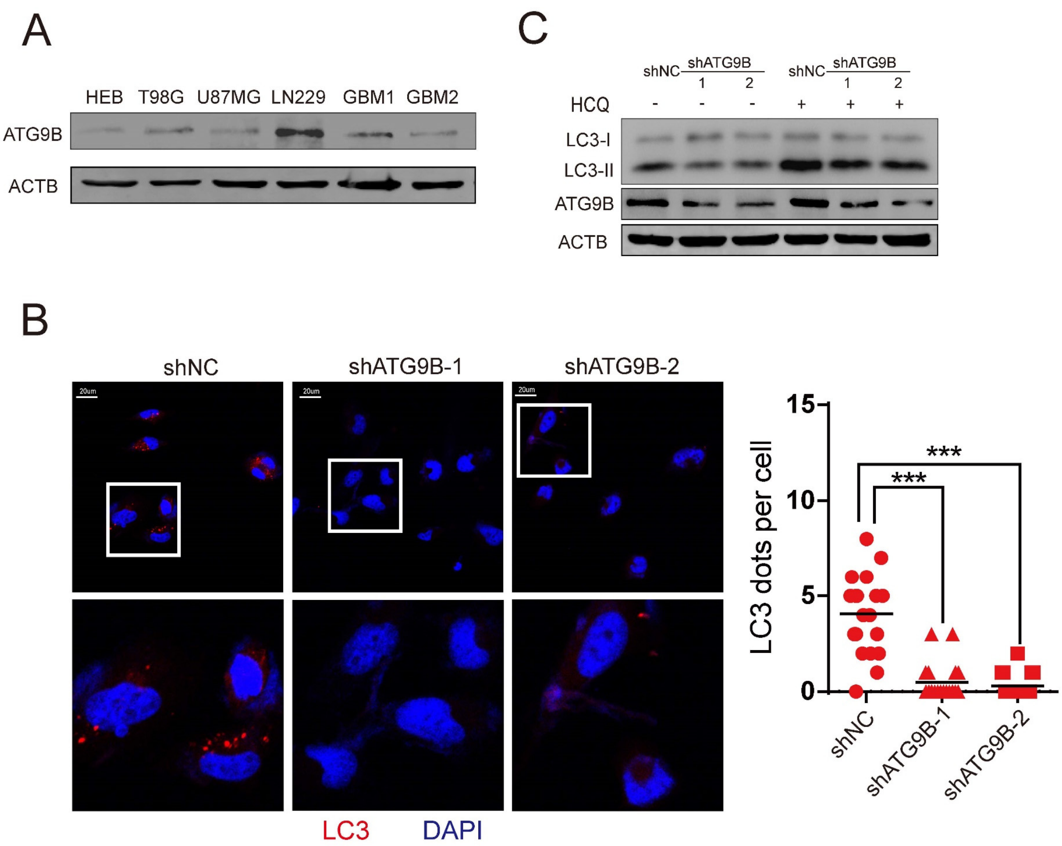

Figure S2. Knock-down of ATG9B inhibits autophagy flux in gliomas. (A) The expression of ATG9B was detected in glial cells, glioma cell lines and primary glioma cells. (B) Representative images of LC3 staining in LN229 cells expressing shNC/shATG9-1/shATG9B-2, with a magnified image (left panel). LC3 dots per cell were quantified (right panel). The scale bar represents 20 μm. Data are shown as scatter plots and means. n=20. (C) With or without hydroxychloroquine (HCQ, 10 μM) treatment, LC3-II accumulation was measured in LN229 cells expressing shNC/shATG9-1/shATG9B-2. ***P<0.001.

Figure S3

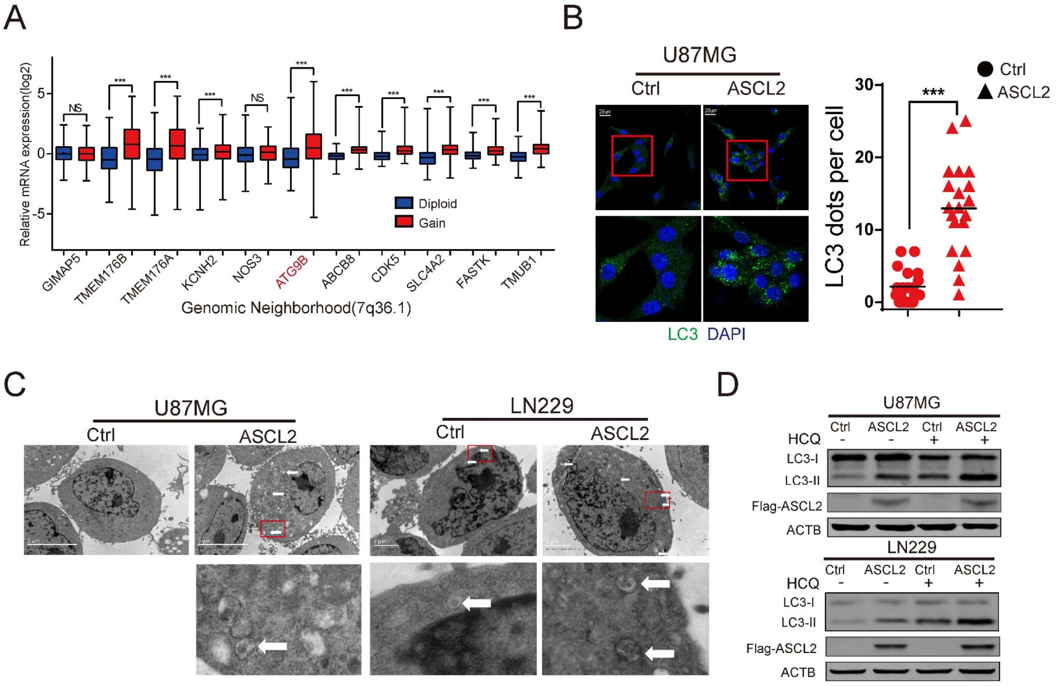

Figure S3. The mRNA expression of genomic neighborhood of *ATG9B* was not consistent and ASCL2 overexpression activates autophagy. (A) mRNA expression of the indicated genes in the indicated gene gain/diploid groups. The patients were from TCGA LGGGBM dataset. The genes were in the Genomic Neighborhood of *ATG9B,* which was located on 7q36.1. (B) Representative images of LC3 staining in U87MG cells overexpressing Ctrl/ASCL2, with magnified images (left panel). The number of LC3 dots per cell was quantified (right panel). Data are shown as scatter plots and means. n=20. (C) Representative electron micrographs of autophagic vesicles or autophagosomes of U87MG/LN229 cells overexpressing Ctrl/ASCL2. Arrows denote autophagosomes. Magnified images are shown. (D) With or without

hydroxychloroquine (HCQ, 10 μM), LC3-II accumulation was measured in U87MG/LN229 cells overexpressing Ctrl/ASCL2. **P<0.01, ***P<0.001.

Figure S4

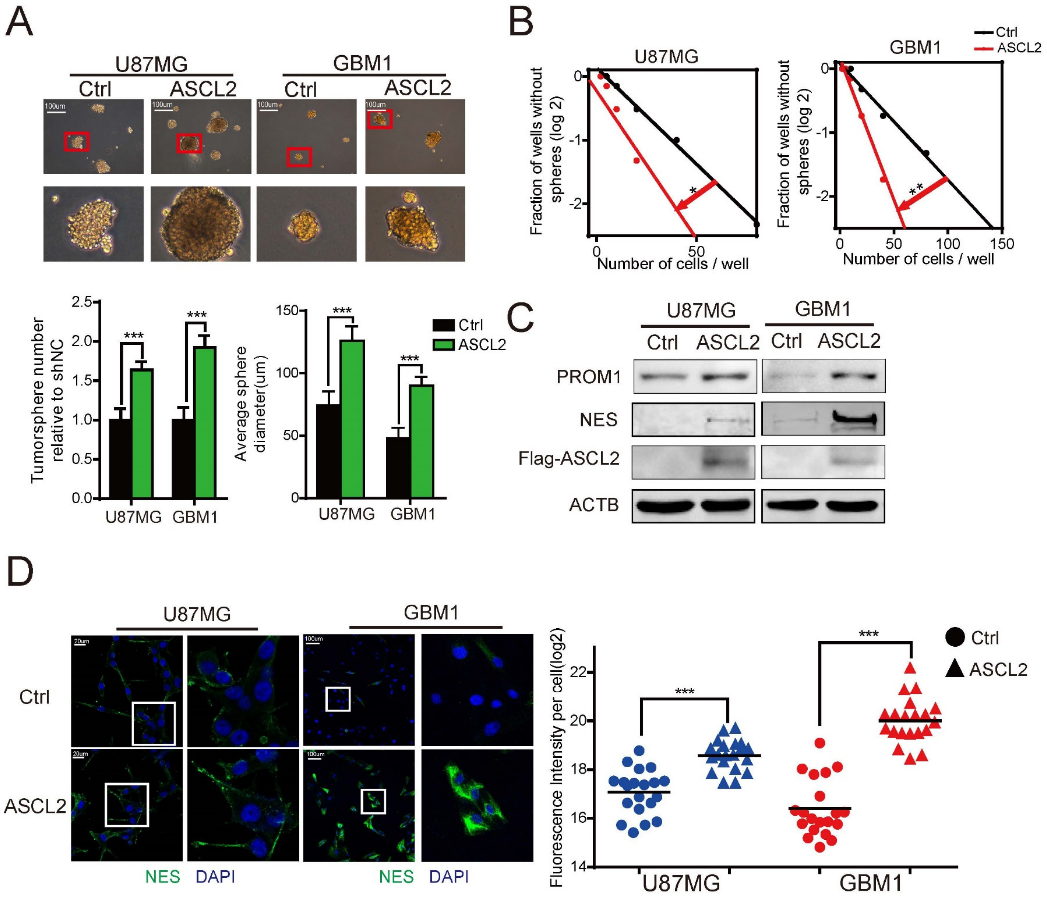

Figure S4. Enforced expression of ASCL2 improves stemness phenotype. (A) Representative images of tumorspheres in U87MG/GBM1 cells overexpressing Ctrl/ASCL2, with a magnified image (upper panel). The numbers and diameters of tumorspheres were quantified (lower panel). The data were collected on the 7th day after cell placement. (B) In vitro limiting dilution assay of U87MG/GBM1 cells overexpressing Ctrl/ASCL2. The data were collected on the 7th day after cell placement. (C) Protein level of PROM1/NES in U87MG/LN229 cells overexpressing Ctrl/ASCL2. (D) Representative images of NES staining and magnified images of the indicated cells overexpressing Ctrl/ASCL2 are shown (left panel). Fluorescence intensity per cell was

quantified (right panel). The scale bar is as shown. Data are shown as scatter plots and means. n=20. *$P<0.05$, **$P<0.01$, ***$P<0.001$.

Figure S5

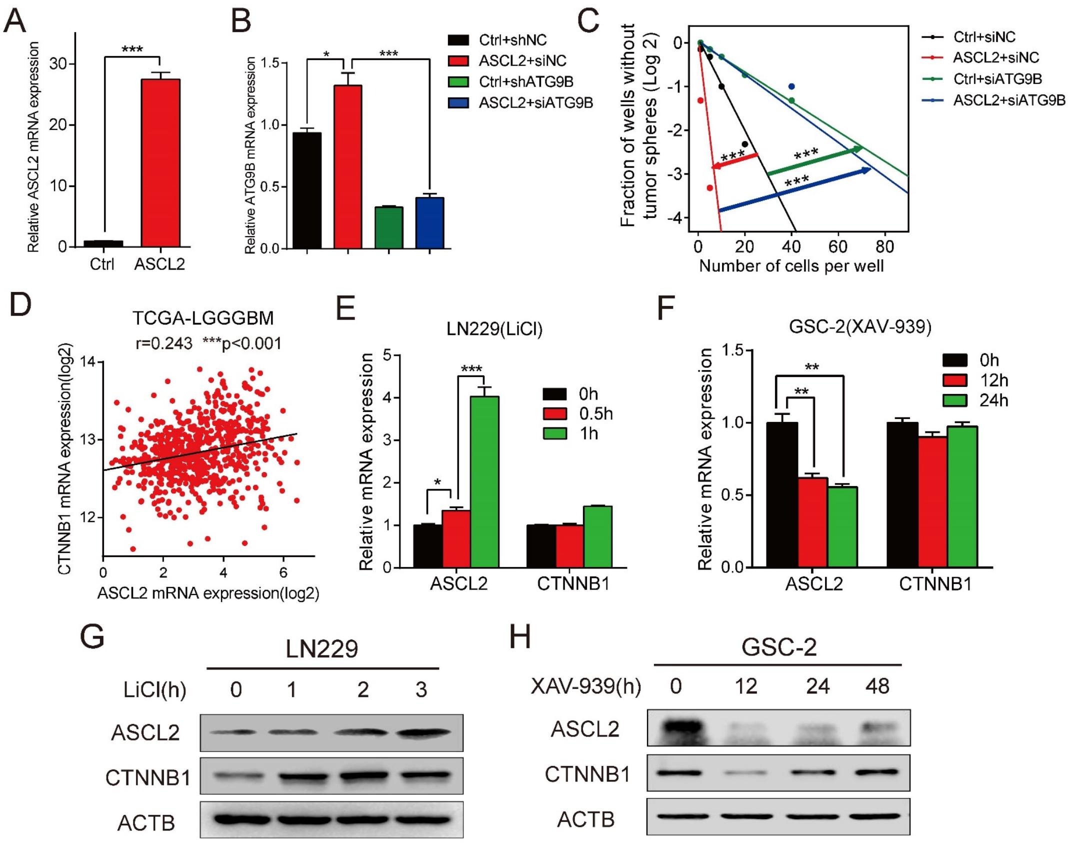

Figure S5. ATG9B is indispensable for ASCL2-mediated in glioma stem cell and ASCL2 is regulated by CTNNB1 in gliomas. (A) mRNA expression of ASCL2 in GSC-1 cells overexpressing Ctrl/ASCL2. (B) mRNA expression of ATG9B in GSC-1 cells expressing Ctrl/ASCL2 and siNC/siATG9B. (C) Limiting dilution assay was performed in GSC-1 cells expressing Ctrl/ASCL2 and siNC/siATG9B. (D) Correlation analysis of the expression of ASCL2 and CTNNB1 in the TCGA LGGGBM dataset (n=669). Pearson's r test. (E, F) mRNA expression of ASCL2 in LN229 cells treated with LiCl (activator of canonical wnt/β-catenin signaling, 20 mM) and GSC-1 cells treated with XAV-939 (inhibitor of canonical wnt/β-catenin signaling, 1 μM) for indicated times. (G, H) ASCL2 expression were detected by immunoblotting in LN229

cells treated with LiCl (20 mM) and GSC-1 cells treated with XAV-939 (1 μM) for indicated times.

Figure S6

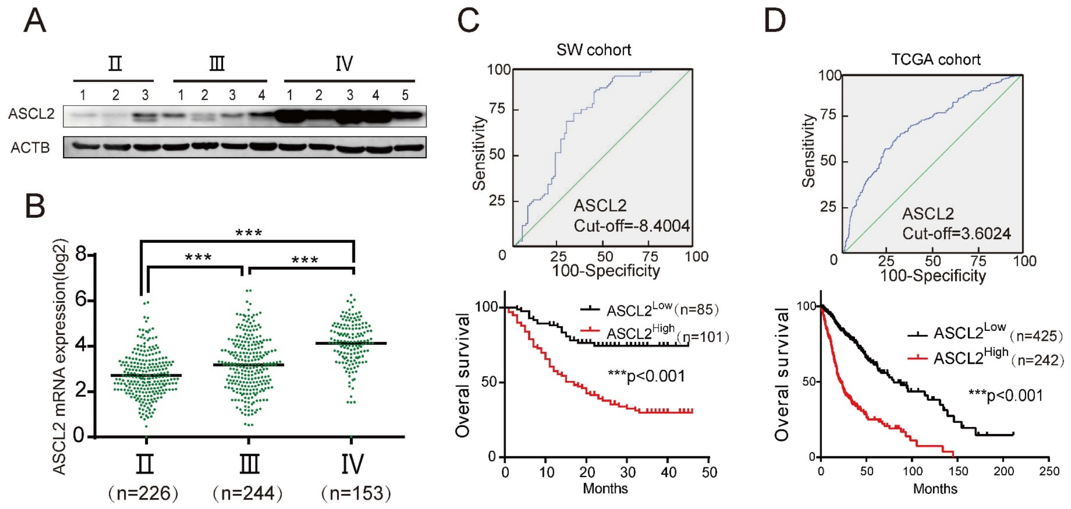

Figure S6. ASCL2 is correlated with poor prognosis in gliomas. (A) ASCL2 expression was measured by immunoblotting in glioma samples of different grades. **(B)** The ASCL2 mRNA expression of the TCGA cohort was summarized with different grades (n=623). **(C, D)** ROC analysis (upper panel) and Kaplan–Meier survival analysis (lower panel) were performed on data from glioma patients according to ATG9B protein expression in the SW cohort and ATG9B mRNA expression in the TCGA cohort.

Figure S7

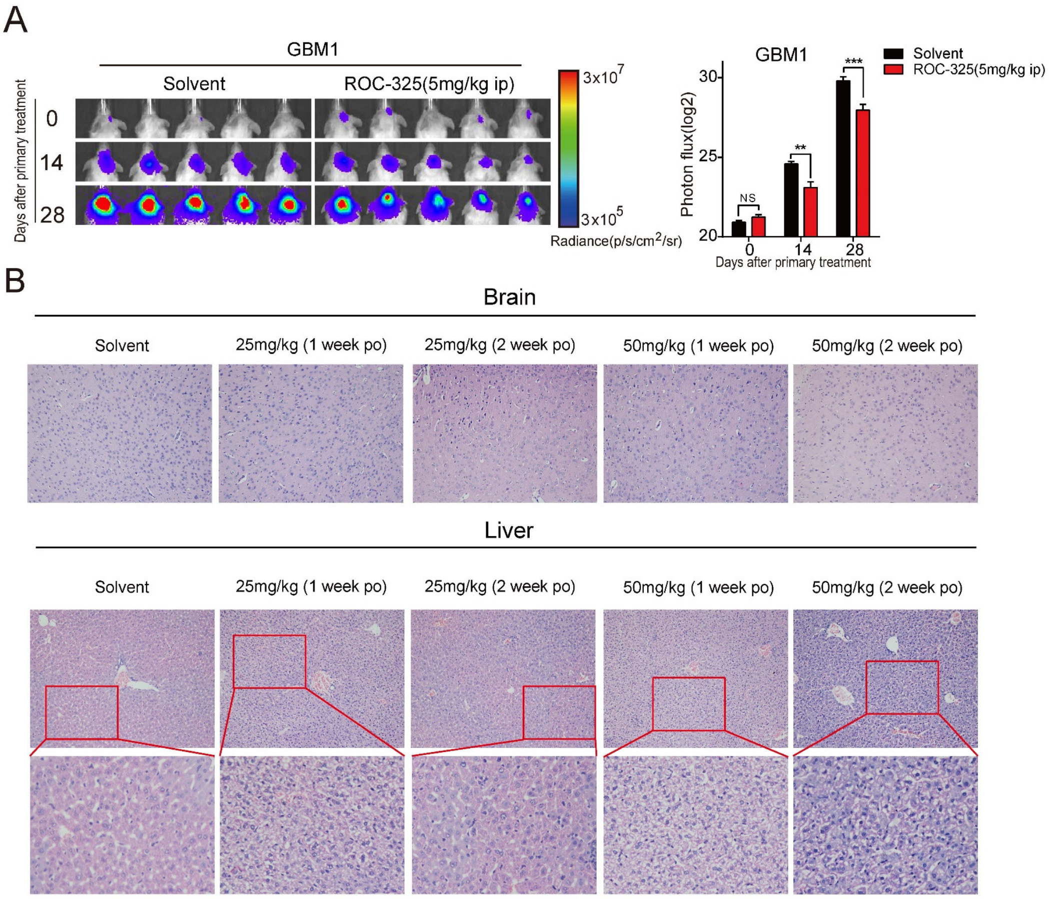

Figure S7. ROC-325 inhibits proliferation of intracranial xenografts and has no severe systemic side effects. (A) Representative bioluminescence images and quantification of intracranial xenografts derived from GBM1 cells treated with solvent/ROC-325 (5 mg/kg, 5 days a week, ip). n=10. **(B)** HE staining of brain and liver tissues from C57BL/6 mice treated with solvent/ROC-325 in Figure 6K.

Figure S8

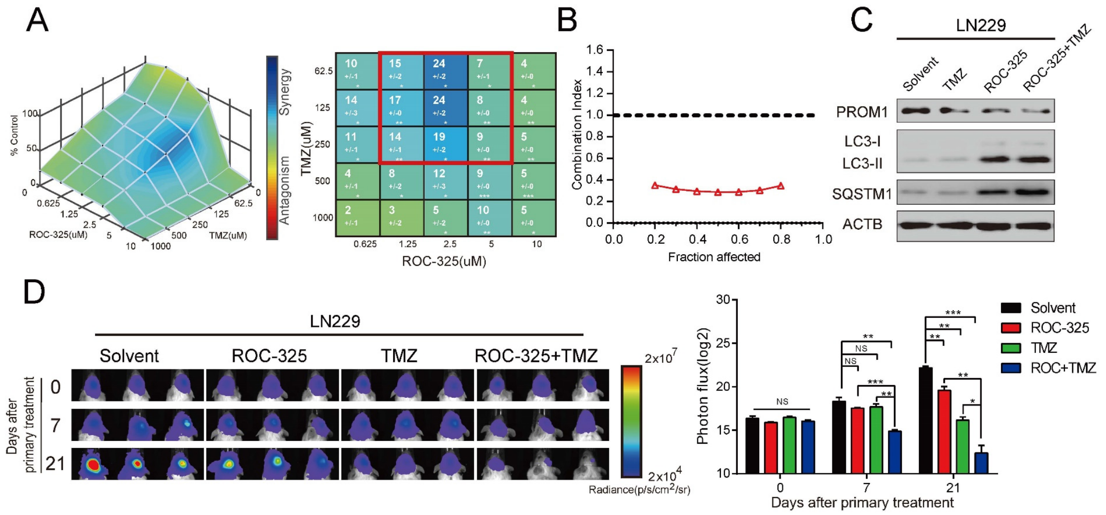

Figure S8. ROC-325 sensitizes glioma cells to TMZ. (A) Synergy surface plots in LN229 cells show increase in synergy between ROC-325 and TMZ (n=3). The data in red box was analyzed in panel B. (B) The interaction between ROC-325 and TMZ on cell killing was examined by the median-effect method of Chou–Talalay. Data are plotted as Fraction affected versus Combination Index (CI). CI < 1 represent synergistic interactions. (C) The expression of indicated molecules were detected with immunoblotting in LN229 cells treated with ROC-325(2.5 μM) and/or TMZ (250 μM) for 48 hr. (D) Bioluminescence images of intracranial xenografts derived from LN229 cells treated with ROC-325(5 mg/kg, 5 days a week, ip) and/or TMZ (5 mg/kg, 5 days a week, ip). n=3. Bioluminescence quantification of the intracranial xenografts in right panel.

Supplementary Table 1: the clinicopathologic mutational and survival information of patients with glioma in southwest hospital

Case ID	Gender	Age	Position	ASCL2 IOD(log2)	ATG9B IOD(log2)	Histology	WHO grade	Integrated Diagnosis	IDH1 mutant	1p/19q codeletion	MGMT Methylation
1	Male	43	ght frontotemporal lobe and basal gan	-12.35362775	-6.902785852	Oligodendroglioma	II	Oligodendroglioma, NOS	-	+	+
2	Male	34	Right parietal occipital lobe	-4.957338236	-4.796658369	Glioblastoma	IV	Glioblastoma, IDH-wildtype	-	-	-
3	Female	48	Right parietal occipital lobe	-7.820569138	-6.247068283	Glioblastoma	IV	Glioblastoma, IDH-wildtype	-	-	+
4	Male	35	Left cerebellum	-9.768927093	-8.56627174	Astrocytoma	II	Diffuse astrocytoma, IDH-mutant	+	-	+
5	Female	67	Left frontal lobe	-7.642458157	-5.367938033	Glioblastoma	IV	Glioblastoma, IDH-wildtype	-	-	+
6	Male	27	Left temporal lobe	-6.655160584	-7.930201665	Glioblastoma	IV	Glioblastoma, IDH-mutant	+	-	-
7	Female	63	Right frontal lobe	-8.707785712	-5.258620279	Glioblastoma	IV	Glioblastoma, IDH-wildtype	-	-	+
8	Male	62	Left frontal lobe	-8.361343406	-8.312301377	Oligoastrocytoma	III	Anaplastic astrocytoma, IDH-wildtype	-	-	+
9	Male	32	Left frontal lobe	-9.945639774	-7.611406698	Glioblastoma	IV	Glioblastoma, IDH-wildtype	-	-	+
10	Male	63	Right frontal lobe	-7.05554787	-4.651154066	Glioblastoma	IV	Glioblastoma, IDH-wildtype	-	-	-
11	Male	29	Left frontal lobe	-11.24046345	-10.98673321	Astrocytoma	II	Diffuse astrocytoma, IDH-mutant	+	-	-
12	Male	63	Right temporal occipital lobe	-8.015055899	-5.955869129	Glioblastoma	IV	Glioblastoma, IDH-wildtype	-	-	+
13	Male	52	frontotemporal lobe and corpus callo	-6.501780954	-6.660809728	Glioblastoma	IV	Glioblastoma, IDH-wildtype	-	-	+
14	Female	48	Right fronto parietal lobe	-10.41700183	-6.32908042	Astrocytoma	II	Diffuse astrocytoma, IDH-mutant	+	-	-
15	Female	54	Right temporal occipital lobe	-3.900225745	-2.906708827	Glioblastoma	IV	Glioblastoma, IDH-wildtype	-	-	+
16	Male	35	Left frontal lobe	-8.630007798	-8.32366402	Astrocytoma	II	Diffuse astrocytoma, IDH-wildtype	-	-	-
17	Male	44	Right frontal lobe	-9.236180674	-7.046914449	Astrocytoma	III	Anaplastic astrocytoma, IDH-mutant	+	-	+
18	Female	20	Left temporal occipital lobe	-10.16684872	-8.967804975	Astrocytoma	III	Anaplastic astrocytoma, IDH-wildtype	-	-	-
19	Female	44	Right fronto parietal lobe	-7.700197215	-5.498471421	Oligodendroglioma	III	ligodendroglioma, IDH-mutant and 1p/1	+	+	+
20	Female	74	Right frontal lobe	-8.444382694	-6.850382569	Oligodendroglioma	III	Anaplastic oligodendroglioma, NOS	-	-	+
21	Male	32	Left frontal lobe	-12.08103591	-10.03476621	Astrocytoma	II	Diffuse astrocytoma, IDH-mutant	+	-	+
22	Male	27	Left frontotemporal lobe	-10.48910032	-9.273680983	Oligoastrocytoma	II	endroglioma, IDH-mutant and 1p/19q-cc	+	+	+
23	Male	55	Left temporal parietal occipital lobe	-8.046433295	-7.304065996	NA	II	NA	-	-	+
24	Male	50	Left frontal lobe	-9.692027604	-9.18165328	Oligodendroglioma	II	endroglioma, IDH-mutant and 1p/19q-cc	+	+	+
25	Male	62	Right temporal parietal occipital lobe	-5.366973082	-4.689801636	Glioblastoma	IV	Glioblastoma, IDH-wildtype	-	-	+
26	Male	29	Left frontal lobe	-10.29711597	-7.509506074	Oligoastrocytoma	II	endroglioma, IDH-mutant and 1p/19q-cc	+	+	-
27	Male	42	Left parietal occipital lobe	-6.744032456	-8.372976158	Oligodendroglioma	III	ligodendroglioma, IDH-mutant and 1p/1	+	+	+
28	Male	30	Right temporal occipital lobe	-6.930050677	-6.75928257	Astrocytoma	III	Anaplastic astrocytoma, IDH-mutant	+	-	+
29	Female	59	Right parietal occipital lobe	-8.772138545	-8.545767977	Glioblastoma	IV	Glioblastoma, IDH-wildtype	-	-	+
30	Male	67	Left parietal occipital lobe	-8.620673577	-5.354088054	Glioblastoma	IV	Glioblastoma, IDH-wildtype	-	-	+
31	Male	19	Cerebellum	-8.977493348	-10.35431519	Astrocytoma	II	Diffuse astrocytoma, IDH-wildtype	-	-	NA
32	Female	67	Right parietal lobe	-6.534340526	-5.074152561	Glioblastoma	IV	Glioblastoma, IDH-wildtype	-	-	+
33	Male	55	Left temporal occipital lobe	-6.895053252	-6.005751566	Glioblastoma	IV	Glioblastoma, IDH-wildtype	-	-	-
34	Female	35	Left frontal lobe	-7.727545613	-5.610242432	Glioblastoma	IV	Glioblastoma, IDH-mutant	+	+	+
35	Male	40	Right frontal lobe	-7.524556404	-5.405603204	Astrocytoma	III	Anaplastic astrocytoma, IDH-mutant	+	-	-
36	Male	35	Right parietal lobe	-7.248627939	-4.913606734	Glioblastoma	IV	Glioblastoma, IDH-mutant	+	+	+
37	Male	52	Right frontal lobe	-6.327205412	-4.662110532	Glioblastoma	IV	Glioblastoma, IDH-wildtype	-	-	+
38	Female	43	Right frontal lobe	-9.777397144	-7.331837064	Astrocytoma	III	Anaplastic astrocytoma, IDH-wildtype	-	-	+
39	Male	46	Right temporal lobe	-7.710219342	-5.279090332	Glioblastoma	IV	Glioblastoma, IDH-wildtype	-	-	-
40	Female	42	Left frontal lobe	-7.13947988	-7.041843129	Glioblastoma	IV	Glioblastoma, IDH-wildtype	-	-	-
41	Male	42	Right frontal lobe	-12.86845341	-9.220383599	Astrocytoma	II	Diffuse astrocytoma, IDH-mutant	+	-	-
42	Female	51	Left fronto parietal lobe	-7.766621227	-6.517300276	Glioblastoma	IV	Glioblastoma, IDH-mutant	+	+	+
43	Male	39	Right frontal lobe and corpus callosun	-7.677691362	-9.270896976	Astrocytoma	III	Anaplastic astrocytoma, IDH-mutant	+	-	-
44	Female	28	Left parietal lobe	-7.802288849	-7.598783885	Glioblastoma	IV	Glioblastoma, IDH-mutant	+	-	+
45	Female	30	Midbrain	-13.31660123	-6.583270633	Astrocytoma	II	Diffuse astrocytoma, IDH-wildtype	-	-	-
46	Female	51	Cervical spinal cord	-6.852453484	-6.021278483	Oligoastrocytoma	III	Anaplastic astrocytoma, IDH-wildtype	-	-	-
47	Female	31	Left temporal lobe	-9.358865469	-5.077760543	Astrocytoma	II	Diffuse astrocytoma, IDH-wildtype	-	-	-
48	Female	52	Right parietal occipital lobe	-6.64528337	-5.699040935	Glioblastoma	IV	Glioblastoma, IDH-wildtype	-	-	-
49	Male	19	Right temporal lobe	-7.964361888	-9.06242179	Astrocytoma	II	Diffuse astrocytoma, IDH-wildtype	-	-	-
50	Male	54	Right temporal lobe	-7.021865895	-4.577312721	Glioblastoma	IV	Glioblastoma, IDH-wildtype	-	-	+
51	Male	54	Right temporal occipital lobe	-8.095607314	-8.696705854	Astrocytoma	III	Anaplastic astrocytoma, IDH-mutant	+	-	-
52	Male	64	Right parietal occipital lobe	-7.341078299	-6.891369458	Glioblastoma	IV	Glioblastoma, IDH-wildtype	-	+	-
53	Female	29	Left temporal lobe	-9.576433416	-7.521299381	Astrocytoma	II	Diffuse astrocytoma, IDH-wildtype	-	-	-
54	Female	36	Right frontal lobe	-9.425335782	-7.288239196	Astrocytoma	II	Diffuse astrocytoma, IDH-wildtype	-	-	+
55	Male	44	Right temporal lobe	-9.990621369	-8.157691176	Astrocytoma	II	Diffuse astrocytoma, IDH-mutant	+	-	+
56	Male	19	Right cerebellum	-6.860548983	-6.432392231	Astrocytoma	III	Anaplastic astrocytoma, IDH-wildtype	-	-	NA
57	Female	42	Left frontal lobe	-8.68762744	-6.380733742	Oligodendroglioma	II	endroglioma, IDH-mutant and 1p/19q-cc	+	+	+
58	Male	45	Left frontal lobe and corpus callosum	-7.621641232	-7.947342725	Oligoastrocytoma	III	ligodendroglioma, IDH-mutant and 1p/1	+	+	+
59	Male	45	lateral frontal lobe and corpus callosu	-9.413952956	-6.592307288	Astrocytoma	II	Diffuse astrocytoma, IDH-mutant	+	-	+
60	Female	32	Left temporal lobe	-6.746221913	-4.689065893	Astrocytoma	III	Anaplastic astrocytoma, IDH-mutant	+	-	+
61	Female	55	Right parietal lobe	-7.436672626	-3.882858866	Glioblastoma	IV	Glioblastoma, IDH-wildtype	-	-	-
62	Male	69	Right frontal lobe	-8.19712877	-5.479915283	Astrocytoma	III	Anaplastic astrocytoma, IDH-wildtype	-	-	-
63	Female	52	Right frontal lobe	-10.2692986	-6.161564214	NA	IV	Glioblastoma, IDH-mutant	+	-	+
64	Male	26	Left parietal lobe	-9.062441849	-8.868611244	Astrocytoma	II	Diffuse astrocytoma, IDH-mutant	+	-	-
65	Female	40	Left frontotemporal parietal lobe	-11.67631696	-6.981441983	Oligodendroglioma	III	ligodendroglioma, IDH-mutant and 1p/1	+	+	+

66	Male	37	Left frontal lobe	-8.591174606	-5.231567985	Glioblastoma	IV	Glioblastoma, IDH-mutant	+	-	-
67	Female	24	Right frontal lobe	-8.470814971	-6.630602491	Oligoastrocytoma	II	endroglioma, IDH-mutant and 1p/19q-cc	+	+	NA
68	Male	47	lateral frontal lobe and corpus callosu	-6.334959363	-6.222553312	Astrocytoma	III	Anaplastic astrocytoma, IDH-mutant	+	-	+
69	Male	52	Left frontal lobe	-7.573550335	-6.329436404	Oligodendroglioma	III	ligodendroglioma, IDH-mutant and 1p/1	+	+	+
70	Male	49	Left temporal lobe	-8.512846186	-5.160093167	Astrocytoma	III	Anaplastic astrocytoma, IDH-mutant	+	-	+
71	Male	67	Right frontal lobe	-8.804115713	-6.517369014	Glioblastoma	IV	Glioblastoma, IDH-mutant	+	-	-
72	Female	46	Left temporal occipital lobe	-9.856856462	-6.719318939	Astrocytoma	II	Diffuse astrocytoma, IDH-wildtype	-	-	+
73	Female	49	Right parietal lobe	-7.30100386	-5.655987061	Astrocytoma	III	Anaplastic astrocytoma, IDH-mutant	+	-	+
74	Male	48	teral corpus callosum and lateral vent	-8.497699893	-4.352232391	Astrocytoma	III	Anaplastic astrocytoma, IDH-mutant	+	-	+
75	Male	64	Right frontotemporal lobe	-7.5854128	-5.835664514	Oligodendroglioma	III	ligodendroglioma, IDH-mutant and 1p/1	+	+	+
76	Female	62	Left frontal lobe	-9.711587116	-6.573439563	Glioblastoma	IV	Glioblastoma, IDH-wildtype	-	-	+
77	Male	10	Left frontal lobe	-7.315716324	-7.178557349	Glioblastoma	IV	Glioblastoma, IDH-wildtype	-	-	+
78	Male	31	Left parietal occipital lobe	-7.537867683	-5.865425115	Astrocytoma	III	Anaplastic astrocytoma, IDH-wildtype	-	-	-
79	Male	27	Right frontal lobe	-8.238377126	-9.2622775	Astrocytoma	II	Diffuse astrocytoma, IDH-wildtype	-	-	+
80	Male	35	Left frontotemporal lobe	-9.125859341	-10.36007309	Astrocytoma	II	Diffuse astrocytoma, IDH-mutant	+	-	+
81	Female	49	Left hippocampus and temporal lobe	-7.896429281	-7.46455892	Astrocytoma	II	Diffuse astrocytoma, IDH-wildtype	-	-	-
82	Male	44	Left ventricle	-6.885395521	-5.467273516	Glioblastoma	IV	Glioblastoma, IDH-wildtype	-	-	+
83	Male	54	Superior cervical spinal cord	-10.07795062	-11.23150455	Astrocytoma	II	Diffuse astrocytoma, IDH-wildtype	-	-	+
84	Female	30	Right parietal lobe	-7.639604797	-6.548476606	Glioblastoma	IV	Glioblastoma, IDH-wildtype	-	-	-
85	Female	22	Right middle cranial fossa	-6.314419842	-5.944414709	Glioblastoma	IV	Glioblastoma, IDH-wildtype	-	-	-
86	Male	48	Left fronto parietal lobe	-8.533175987	-7.711941062	Astrocytoma	III	Anaplastic astrocytoma, IDH-wildtype	-	-	+
87	Male	44	Left frontotemporal lobe	-8.085686432	-6.226478275	Glioblastoma	IV	Glioblastoma, IDH-wildtype	-	-	-
88	Male	27	Brainstem	-6.481255461	-4.452373193	Astrocytoma	II	Diffuse astrocytoma, NOS	NA	-	NA
89	Male	51	Left temporal occipital lobe	-6.942235278	-3.966073609	Glioblastoma	IV	Glioblastoma, IDH-wildtype	-	+	+
90	Male	45	Left frontal lobe	-8.076937164	-9.109809081	Oligodendroglioma	III	ligodendroglioma, IDH-mutant and 1p/1	+	+	+
91	Male	30	Right fronto parietal lobe	-9.972377168	-6.25913314	Astrocytoma	III	Anaplastic astrocytoma, IDH-mutant	+	-	+
92	Male	58	Left temporal parietal occipital lobe	-8.191896949	-7.643170504	Glioblastoma	IV	Glioblastoma, IDH-wildtype	-	-	-
93	Male	38	Right temporal parietal lobe	-10.1174159	-6.45254816	Glioblastoma	IV	Glioblastoma, IDH-wildtype	-	-	-
94	Female	39	Right frontal lobe	-11.13039401	-10.15221588	Oligodendroglioma	II	endroglioma, IDH-mutant and 1p/19q-cc	+	+	+
95	Male	19	Right frontal lobe	-12.33141402	-8.866735941	Astrocytoma	II	Diffuse astrocytoma, IDH-mutant	+	-	-
96	Male	47	Left temporal lobe	-11.0549391	-9.788138048	Astrocytoma	II	Diffuse astrocytoma, IDH-mutant	+	-	-
97	Female	37	Right frontotemporal basal ganglia	-14.2555197	-8.094930117	Oligodendroglioma	II	endroglioma, IDH-mutant and 1p/19q-cc	+	+	+
98	Female	43	Right temporal lobe	-5.555422241	-5.964947665	Glioblastoma	IV	Glioblastoma, IDH-mutant	+	-	-
99	Male	39	Left frontal lobe	-7.05830551	-4.879815122	Glioblastoma	IV	Glioblastoma, IDH-mutant	+	-	-
100	Female	39	Left fronto parietal lobe	-6.836839193	-6.608767822	Oligoastrocytoma	III	ligodendroglioma, IDH-mutant and 1p/1	+	+	-
101	Female	25	Right temporal lobe	-8.439411726	-6.638299825	Astrocytoma	III	Anaplastic astrocytoma, IDH-wildtype	-	-	-
102	Male	22	Left parietal occipital lobe	-9.49028715	-5.052223983	Glioblastoma	IV	Glioblastoma, IDH-wildtype	-	-	-
103	Male	30	Right fronto parietal lobe	-8.540449744	-9.919461565	Astrocytoma	II	Diffuse astrocytoma, IDH-mutant	+	-	-
104	Female	49	Left frontotemporal lobe	-8.47536129	-5.003175504	Astrocytoma	III	Anaplastic astrocytoma, IDH-wildtype	-	-	-
105	Female	68	Left temporal lobe	-4.977966235	-5.574819468	Glioblastoma	IV	Glioblastoma, IDH-wildtype	-	-	-
106	Female	21	Right fronto parietal lobe	-11.77332006	-7.86033972	Oligodendroglioma	II	Diffuse astrocytoma, IDH-mutant	+	-	-
107	Female	27	Left Island leaf	-7.994271272	-8.361059645	Oligodendroglioma	II	endroglioma, IDH-mutant and 1p/19q-cc	+	+	-
108	Male	49	Left frontotemporal lobe	-9.150189312	-9.591203545	Astrocytoma	II	Diffuse astrocytoma, IDH-wildtype	-	-	+
109	Female	35	Right parietal lobe	-11.80505519	-6.547563805	Astrocytoma	II	Diffuse astrocytoma, IDH-wildtype	-	-	-
110	Male	41	Right occipital lobe	-7.62314201	-4.531798006	Glioblastoma	IV	Glioblastoma, IDH-wildtype	-	-	-
111	Male	48	Right temporal lobe	-7.348036056	-5.713208113	Glioblastoma	IV	Glioblastoma, IDH-wildtype	-	-	-
112	Female	48	Right cerebellum	-9.969854085	-9.369969171	Astrocytoma	II	Diffuse astrocytoma, IDH-wildtype	-	-	-
113	Female	31	Left frontal lobe	-9.671566561	-8.148357183	Oligoastrocytoma	II	Diffuse astrocytoma, IDH-wildtype	-	-	-
114	Male	56	Right frontal lobe	-7.639627242	-5.280212548	Glioblastoma	IV	Glioblastoma, IDH-wildtype	-	-	-
115	Male	47	Right parietal occipital lobe	-9.222645772	-8.30932289	Astrocytoma	II	Diffuse astrocytoma, IDH-mutant	+	-	-
116	Male	29	otemporal lobe, insular lobe and basa	-8.123576413	-5.608099707	Oligoastrocytoma	II	Diffuse astrocytoma, IDH-mutant	+	-	+
117	Female	23	Right frontal lobe	-9.310632667	-7.0113531	Oligoastrocytoma	II	Diffuse astrocytoma, IDH-mutant	+	-	+
118	Female	61	Right frontal lobe and corpus callosun	-10.27277559	-7.235287199	Oligodendroglioma	II	endroglioma, IDH-mutant and 1p/19q-cc	+	+	+
119	Male	51	Left frontal and temporal part	-8.683348619	-5.3857682	Astrocytoma	II	Diffuse astrocytoma, IDH-mutant	+	-	+
120	Male	74	Left temporal lobe	-6.989224049	-3.795009122	Glioblastoma	IV	Glioblastoma, IDH-wildtype	-	-	-
121	Female	19	Right temporal lobe	-7.597976807	-7.229594418	Oligoastrocytoma	II	Diffuse astrocytoma, IDH-wildtype	-	-	-
122	Female	53	rontotemporal lobe and parietooccipit	-8.34650259	-3.44571192	Glioblastoma	IV	Glioblastoma, IDH-wildtype	-	-	+
123	Male	50	Left temporal parietal lobe	-5.126205237	-3.562866956	Glioblastoma	IV	Glioblastoma, IDH-mutant	+	-	+
124	Female	63	Right fronto parietal lobe	-7.420609873	-4.207203302	Astrocytoma	III	Anaplastic astrocytoma, IDH-wildtype	-	-	-
125	Male	23	Left temporal lobe	-12.10893873	-5.666366746	Oligodendroglioma	II	endroglioma, IDH-mutant and 1p/19q-cc	+	+	-
126	Male	55	ontotemporal lobe and parietooccipita	-7.443396272	-4.073528313	Glioblastoma	IV	Glioblastoma, IDH-wildtype	-	-	-
127	Female	47	Left frontal lobe	-8.886824525	-4.525171371	Glioblastoma	IV	Glioblastoma, IDH-wildtype	-	-	-
128	Male	53	Left parietal occipital lobe	-6.546490249	-4.225431462	Glioblastoma	IV	Glioblastoma, IDH-wildtype	-	-	-
129	Male	18	Left temporal lobe	-10.27523773	-7.970719721	Astrocytoma	II	Diffuse astrocytoma, IDH-wildtype	-	-	+
130	Male	43	Right frontal lobe	-11.3800836	-7.343684289	Astrocytoma	II	Diffuse astrocytoma, IDH-mutant	+	-	-
131	Male	52	Left frontal lobe and corpus callosum	-9.494448748	-6.483002912	Astrocytoma	II	Diffuse astrocytoma, IDH-mutant	+	-	+
132	Male	38	Right temporal occipital lobe	-8.75103509	-3.993161948	Astrocytoma	III	Anaplastic astrocytoma, IDH-mutant	+	-	-

133	Male	52	Right basal ganglia and temporal lobe	-7.316858432	-5.745140856	Glioblastoma	IV	Glioblastoma, IDH-mutant	+	-	-
134	Female	31	Right frontal lobe	-9.024921368	-4.684797286	Astrocytoma	II	Diffuse astrocytoma, IDH-mutant	+	-	-
135	Female	48	Left frontal lobe	-6.934633095	-3.777468658	Glioblastoma	IV	Glioblastoma, IDH-wildtype	-	-	+
136	Female	43	Right frontal lobe	-9.318516397	-8.158741555	Astrocytoma	II	Diffuse astrocytoma, IDH-mutant	+	-	-
137	Male	25	Left parietal lobe	-9.26499874	-6.088923672	Astrocytoma	II	Diffuse astrocytoma, IDH-mutant	+	-	-
138	Male	30	Bilateral frontal lobe	-8.784394443	-7.5856362	Astrocytoma	II	endroglioma, IDH-mutant and 1p/19q-cc	+	+	+
139	Male	55	Left frontal lobe	-8.236361698	-3.933505042	Glioblastoma	IV	Glioblastoma, IDH-wildtype	-	-	+
140	Female	35	Left frontal lobe	-8.296360882	-5.787652985	Astrocytoma	II	Diffuse astrocytoma, IDH-mutant	+	-	-
141	Female	49	Right cerebellum and brainstem	-9.479506776	-4.799257214	Astrocytoma	II	Diffuse astrocytoma, NOS	NA	-	-
142	Male	28	Left frontotemporal lobe	-9.000061232	-5.514632347	Oligoastrocytoma	II	endroglioma, IDH-mutant and 1p/19q-cc	+	+	+
143	Male	45	Right frontal lobe	-7.866263119	-2.929694717	Oligoastrocytoma	III	ligodendroglioma, IDH-mutant and 1p/1	+	+	+
144	Male	47	Right frontal lobe	-8.735791475	-5.40754318	Oligodendroglioma	III	ligodendroglioma, IDH-mutant and 1p/1	+	+	+
145	Male	46	Right parietal lobe	-6.295149433	-4.300070816	Glioblastoma	IV	Glioblastoma, IDH-wildtype	-	-	-
146	Male	52	Left frontotemporal lobe	-7.017698252	-4.179118639	Glioblastoma	IV	Glioblastoma, IDH-mutant	+	+	+
147	Female	51	NA	-7.639544944	-4.570733634	Glioblastoma	IV	Glioblastoma, IDH-wildtype	-	-	-
148	Male	51	nporal occipital lobe and temporal par	-7.86188375	-3.786489189	Glioblastoma	IV	Glioblastoma, IDH-wildtype	-	-	-
149	Female	53	Left frontal lobe	-7.197734512	-2.968163124	Glioblastoma	IV	Glioblastoma, IDH-wildtype	-	-	-
150	Female	22	Right frontal lobe	-8.557919283	-5.21221561	Astrocytoma	III	Anaplastic astrocytoma, IDH-wildtype	-	-	+
151	Female	63	Left frontal lobe	-5.088513833	-4.67973619	Glioblastoma	IV	Glioblastoma, IDH-wildtype	-	-	+
152	Female	43	Left parietal lobe	-7.277523607	-8.194092882	Oligodendroglioma	III	NA	NA	+	+
153	Male	58	Left frontal lobe	-8.349461013	-8.527945433	Oligodendroglioma	II	endroglioma, IDH-mutant and 1p/19q-cc	+	+	+
154	Female	48	Left temporal and insular lobes	-9.389463995	-11.12770508	Astrocytoma	II	Diffuse astrocytoma, IDH-mutant	+	-	+
155	Male	45	Left frontal lobe	-7.831731591	-8.258710771	Glioblastoma	IV	Glioblastoma, IDH-wildtype	-	-	-
156	Male	42	Right frontal lobe	-12.6823464	-8.763258985	Astrocytoma	II	Diffuse astrocytoma, IDH-mutant	+	-	+
157	Female	15	Right temporal lobe	-7.003412565	-7.085080098	Oligodendroglioma	III	Anaplastic oligodendroglioma, NOS	+	-	-
158	Female	52	Bilateral frontal lobe	-8.105917288	-6.502651625	Astrocytoma	III	Anaplastic astrocytoma, IDH-wildtype	-	-	-
159	Male	45	Right frontal lobe	-5.229122908	-7.279486652	Oligodendroglioma	III	Anaplastic oligodendroglioma, NOS	+	-	+
160	Male	68	Right frontal lobe and corpus callosun	-7.047083139	-6.265639439	Astrocytoma	III	Anaplastic astrocytoma, IDH-wildtype	-	-	+
161	Male	50	Left temporal lobe	-7.407345722	-4.822291932	Glioblastoma	IV	Glioblastoma, IDH-wildtype	-	+	-
162	Male	44	Left temporal lobe	-6.750513863	-9.280734602	Astrocytoma	III	Anaplastic astrocytoma, IDH-mutant	+	-	+
163	Female	37	Left temporal lobe	-5.378831021	-5.890632492	Glioblastoma	IV	Glioblastoma, IDH-wildtype	-	-	+
164	Male	41	Left frontal lobe	-7.671459474	-7.463167949	Oligoastrocytoma	II	endroglioma, IDH-mutant and 1p/19q-cc	+	+	+
165	Female	36	Left frontal lobe	-6.721831522	-6.874907807	Glioblastoma	IV	Glioblastoma, IDH-wildtype	-	-	-
166	Female	31	Left frontal lobe	-5.976767767	-7.853989092	Glioblastoma	IV	Glioblastoma, IDH-wildtype	-	-	-
167	Male	28	Left frontal lobe	-13.68382685	-9.383763274	Astrocytoma	II	Diffuse astrocytoma, IDH-wildtype	-	-	-
168	Female	38	Right frontal lobe	-9.446724063	-6.011677442	Oligoastrocytoma	II	endroglioma, IDH-mutant and 1p/19q-cc	+	+	+
169	Male	66	Left temporal lobe	-8.808836034	-4.140792379	Glioblastoma	IV	Glioblastoma, IDH-wildtype	-	-	-
170	Male	42	Left frontal lobe	-13.11175892	-7.442064692	Astrocytoma	II	Diffuse astrocytoma, IDH-mutant	+	-	-
171	Male	30	Right temporal lobe	-7.301228709	-5.753759445	Glioblastoma	IV	Glioblastoma, IDH-mutant	+	-	+
172	Male	53	Occipital lobe	-5.029043807	-4.800037782	Glioblastoma	IV	Glioblastoma, IDH-wildtype	-	-	-
173	Male	49	Left occipital lobe	-6.75029977	-5.233739067	Glioblastoma	IV	Glioblastoma, IDH-wildtype	-	-	-
174	Female	61	Right frontotemporal parietal lobe	-6.032600762	-7.107944679	Glioblastoma	IV	Glioblastoma, IDH-wildtype	-	-	+
175	Male	69	Right frontal lobe	-6.114732831	-3.542920547	Glioblastoma	IV	Glioblastoma, IDH-wildtype	-	-	+
176	Female	28	Left fronto parietal lobe	-6.666065716	-2.980675024	Glioblastoma	IV	Glioblastoma, IDH-wildtype	-	-	-
177	Male	40	Right frontal lobe	-6.342898343	-3.47118732	Glioblastoma	IV	Glioblastoma, IDH-wildtype	-	-	-
178	Female	61	Right parietal lobe	-6.450617307	-2.675680592	Astrocytoma	III	Anaplastic astrocytoma, IDH-wildtype	-	-	-
179	Male	45	NA	-7.429805493	-4.199906258	Oligoastrocytoma	II	Diffuse astrocytoma, IDH-wildtype	-	-	-
180	Male	28	Right frontal lobe	-6.509895811	-3.557626354	NA	III	NA	-	-	-
181	Female	68	Left temporal lobe	-6.527495409	-3.192403776	Glioblastoma	IV	Glioblastoma, IDH-wildtype	-	-	-
182	Male	46	Left temporal lobe	-6.032190652	-4.261391466	Glioblastoma	IV	Glioblastoma, IDH-wildtype	-	-	-
183	Female	36	Right frontal lobe	-6.598620252	-5.855815295	Astrocytoma	II	Diffuse astrocytoma, IDH-wildtype	-	-	-
184	Male	61	Right frontal lobe	-6.903522317	-8.294710497	Astrocytoma	III	Anaplastic astrocytoma, IDH-mutant	+	+	-
185	Male	35	Right temporal lobe	-5.828764394	-4.312824736	Glioblastoma	IV	Glioblastoma, IDH-wildtype	-	-	+
186	Female	57	Right temporal lobe	-5.287885904	-3.370636504	Glioblastoma	IV	Glioblastoma, IDH-wildtype	-	-	-
187	Male	47	Left frontal lobe	-8.522071568	-6.892104574	Astrocytoma	III	Anaplastic astrocytoma, IDH-wildtype	-	-	-
188	Female	33	Left frontal lobe	-6.403208491	-5.558352379	Astrocytoma	III	Anaplastic astrocytoma, IDH-mutant	+	-	-
189	Female	42	Left frontal lobe	-6.536853569	-4.890917437	Oligoastrocytoma	III	Anaplastic astrocytoma, IDH-mutant	+	-	-
190	Male	48	Left temporal lobe	-7.247629793	-3.615986235	Glioblastoma	IV	Glioblastoma, IDH-wildtype	-	-	-
191	Male	71	Right temporal occipital lobe	-5.994393255	-3.163941729	Glioblastoma	IV	Glioblastoma, IDH-wildtype	-	-	-
192	Male	37	Right parietal lobe	-9.423582685	-3.91307704	Oligoastrocytoma	II	endroglioma, IDH-mutant and 1p/19q-cc	+	+	+
193	Female	72	Left temporal lobe	-6.568730663	-2.986966102	Glioblastoma	IV	Glioblastoma, IDH-wildtype	-	-	-
194	Male	20	Right temporal lobe	-10.80781003	-6.418458087	NA	II	Diffuse astrocytoma, IDH-wildtype	-	-	-
195	Male	24	Left temporal parietal lobe	-6.961184098	-5.154980341	Glioblastoma	IV	Glioblastoma, IDH-wildtype	-	-	-
196	Male	48	Left frontal and temporal lobes	-7.587215403	-3.675370422	Astrocytoma	III	Anaplastic astrocytoma, IDH-wildtype	-	-	-
197	Male	73	Left frontal lobe	-6.481996444	-4.548809395	Glioblastoma	IV	Glioblastoma, IDH-wildtype	-	-	-
198	Female	53	Right frontal lobe	-9.808247251	-10.50150218	Astrocytoma	III	Anaplastic astrocytoma, IDH-mutant	+	+	+
199	Male	30	Left parietal lobe	-7.440988577	-4.860013896	Astrocytoma	II	Diffuse astrocytoma, IDH-mutant	+	-	-

200	Male	41	Right frontal lobe	-8.310501636	-5.40141863	Oligodendroglioma	II	endroglioma, IDH-mutant and 1p/19q-cc	+	+	+
201	Male	18	Left occipital lobe	-7.183588207	-4.501161431	Glioblastoma	IV	Glioblastoma, IDH-wildtype	-	-	+
202	Male	30	Right frontal lobe	-6.428641518	-6.922803575	Glioblastoma	IV	Glioblastoma, IDH-wildtype	-	-	-
203	Female	22	Left frontal lobe	-8.869839398	-4.74580711	Oligoastrocytoma	II	Diffuse astrocytoma, IDH-wildtype	-	-	-
204	Male	62	Right frontal lobe	-7.824705603	-4.194974941	Glioblastoma	IV	Glioblastoma, IDH-wildtype	-	-	-
205	Female	54	Right temporal lobe	-7.721934492	-4.604151335	Oligodendroglioma	III	Anaplastic oligodendroglioma, NOS	-	+	-
206	Male	20	Right temporal occipital lobe	-6.786517664	-3.521141288	Glioblastoma	IV	Glioblastoma, IDH-wildtype	-	NA	-
207	Male	58	Right temporal lobe	-4.515624089	-3.468329974	Glioblastoma	IV	Glioblastoma, IDH-wildtype	-	-	-
208	Female	36	Left frontal lobe	-8.454073653	-3.81015536	Glioblastoma	IV	Glioblastoma, IDH-wildtype	-	-	-
209	Female	13	Cervicaland thoracic spinal cord	-6.94937223	-5.14779709	Oligodendroglioma	II	Oligodendroglioma, NOS	NA	+	-
210	Female	44	Left temporal lobe	-7.458897446	-3.011278983	Oligoastrocytoma	III	ligodendroglioma, IDH-mutant and 1p/1	+	+	+
211	Female	46	Left temporal lobe	-9.119897826	-6.209572606	Oligodendroglioma	II	Oligodendroglioma, NOS	NA	-	-
212	Male	42	Right frontal lobe	-9.730427103	-8.928409133	Glioblastoma	IV	Glioblastoma, IDH-mutant	+	+	+
213	Male	49	Left frontal lobe	-8.889508841	-13.31048972	Oligoastrocytoma	II	endroglioma, IDH-mutant and 1p/19q-cc	+	+	+
214	Male	53	Left frontotemporal lobe	-9.530232334	-3.572401928	Astrocytoma	III	Anaplastic astrocytoma, IDH-wildtype	-	-	-
215	Female	42	Left frontal lobe	-7.726154688	-10.84549005	Astrocytoma	II	Diffuse astrocytoma, IDH-mutant	+	-	-
216	Female	16	Left temporal lobe	-8.592883017	-5.417088398	Oligodendroglioma	II	Diffuse astrocytoma, IDH-wildtype	-	-	+
217	Male	57	Left parietal lobe	-6.907840891	-4.812034377	Glioblastoma	IV	Glioblastoma, IDH-wildtype	-	-	-
218	Female	18	Cerebellum	-9.114231122	-5.607823398	Astrocytoma	II	Diffuse astrocytoma, IDH-wildtype	-	-	-
219	Female	32	Right frontotemporal lobe	-7.903050757	-4.626428099	Astrocytoma	II	Diffuse astrocytoma, IDH-wildtype	-	-	-
220	Female	24	Right thalamus	-8.23072667	-3.591919524	Glioblastoma	IV	Glioblastoma, IDH-wildtype	-	-	-
221	Female	62	Left frontotemporal lobe	-8.668158985	-4.398389813	Oligoastrocytoma	III	Anaplastic astrocytoma, IDH-wildtype	-	-	-
222	Male	64	Thoracic spinal cord	-7.83537742	-10.76611476	Astrocytoma	II	Diffuse astrocytoma, IDH-wildtype	-	-	-
223	Male	36	Left frontal lobe	-8.958797173	-12.06599675	Oligodendroglioma	II	Oligodendroglioma, NOS	NA	+	-
224	Male	50	Left temporal lobe	-7.298154868	-2.853663343	Glioblastoma	IV	Glioblastoma, IDH-wildtype	-	+	-
225	Male	18	Thoracic and lumbar spinal cord	-6.207084107	-3.717041614	Glioblastoma	IV	Glioblastoma, NOS	NA	-	-
226	Female	26	Left frontal lobe	-7.118531198	-4.116028853	Astrocytoma	II	Diffuse astrocytoma, NOS	NA	-	-
227	Female	49	Left temporal lobe	-11.32948166	-14.73406504	Astrocytoma	II	Diffuse astrocytoma, IDH-mutant	+	-	-
228	Male	61	Left temporal lobe	-5.117320831	-7.15942793	Glioblastoma	IV	Glioblastoma, IDH-wildtype	-	-	+
229	Male	59	Right frontal lobe	-7.80280818	-11.9792154	Oligoastrocytoma	II	Diffuse astrocytoma, IDH-wildtype	-	+	-
230	Male	52	Left temporal lobe	-5.090360946	-4.053166656	Glioblastoma	IV	Glioblastoma, IDH-mutant	+	-	+
231	Female	15	Left parietal lobe	-6.324165966	-4.625599854	Oligodendroglioma	III	Anaplastic oligodendroglioma, NOS	-	-	-
232	Female	61	Left fronto parietal lobe	-6.455202372	-12.50373389	Oligodendroglioma	II	endroglioma, IDH-mutant and 1p/19q-cc	+	+	+
233	Male	46	Left parietal lobe	-8.080233502	-5.701746874	Oligoastrocytoma	II	Diffuse astrocytoma, IDH-wildtype	-	-	-
234	Female	24	Right parietal lobe	-5.331956446	-3.108421879	Astrocytoma	III	Anaplastic astrocytoma, IDH-wildtype	-	-	-
235	Female	30	Left frontal lobe	-11.01634733	-14.23222644	Astrocytoma	II	Diffuse astrocytoma, IDH-wildtype	-	+	+
236	Male	42	Left temporal parietal lobe	-7.468739897	-5.718743837	NA	III	NA	-	-	-
237	Female	20	Left frontal lobe	-5.388583559	-3.443626077	Glioblastoma	IV	Glioblastoma, IDH-wildtype	-	-	-
238	Male	35	Right frontal lobe	-5.600599642	-5.381797109	NA	III	NA	-	-	-
239	Female	62	Frontal lobe	-6.29099227	-5.662224526	Oligodendroglioma	II	Oligodendroglioma, NOS	NA	+	+
240	Male	38	Right frontal lobe	-5.176127159	-5.53970082	Astrocytoma	III	Anaplastic astrocytoma, NOS	NA	-	-
241	Female	46	Right frontal lobe	-4.269724807	-4.551376821	NA	III	NA	-	+	NA
242	Male	43	Left temporal lobe	-5.801269576	-6.330664922	Glioblastoma	IV	Glioblastoma, NOS	NA	-	-
243	Male	60	Left frontal lobe	-5.514869068	-4.910364914	Glioblastoma	IV	Glioblastoma, IDH-wildtype	-	-	+
244	Female	61	Left frontal lobe	-6.892305125	-7.980115097	Astrocytoma	II	Diffuse astrocytoma, IDH-wildtype	-	-	-
245	Female	45	Left temporal lobe	-5.2066005	-4.973628578	Astrocytoma	III	Anaplastic astrocytoma, NOS	NA	-	-
246	Male	64	Right temporal occipital lobe	-5.285563072	-3.767227489	Glioblastoma	IV	Glioblastoma, IDH-wildtype	-	-	+
247	Male	78	Left frontal lobe	-5.378055344	-3.426015518	Glioblastoma	IV	Glioblastoma, IDH-wildtype	-	-	-
248	Female	59	Right frontal lobe	-6.00479961	-5.062791678	Glioblastoma	IV	Glioblastoma, IDH-wildtype	-	-	+
249	Male	62	Frontal lobe	-5.553234262	-8.516592468	Glioblastoma	IV	Glioblastoma, IDH-wildtype	-	-	-
250	Male	50	Left frontal lobe	-5.5495657	-5.046290716	Glioblastoma	IV	Glioblastoma, IDH-wildtype	-	-	+
251	Male	27	Left frontal lobe	-5.861085358	-5.996487692	Oligoastrocytoma	III	Anaplastic astrocytoma, IDH-wildtype	-	-	+
252	Male	33	Left temporal lobe	-5.732195062	-5.141300763	Glioblastoma	IV	Glioblastoma, IDH-wildtype	-	+	+
253	Female	73	Right occipital lobe	-5.633661596	-5.525351073	Glioblastoma	IV	Glioblastoma, IDH-wildtype	-	-	-
254	Male	59	Right frontal lobe	-6.141263688	-6.627115884	Glioblastoma	IV	Glioblastoma, IDH-wildtype	-	-	-
255	Male	36	Right parietal lobe	-5.974784327	-4.737121682	Glioblastoma	IV	Glioblastoma, IDH-wildtype	-	-	+
256	Male	49	Left temporal lobe	-4.864693628	-4.399046678	Glioblastoma	IV	Glioblastoma, IDH-mutant	+	-	+
257	Female	47	Right frontal lobe	-5.742555243	-5.566125973	Astrocytoma	II	Diffuse astrocytoma, NOS	NA	+	-
258	Male	36	Right temporal lobe	-9.846733218	-11.05749566	Astrocytoma	II	Diffuse astrocytoma, IDH-wildtype	-	-	-
259	Male	72	Right occipital lobe	-5.302874722	-5.703268876	Oligodendroglioma	III	Anaplastic oligodendroglioma, NOS	NA	-	-
260	Male	62	Right parietal occipital lobe	-5.628570397	-3.254506753	Glioblastoma	IV	Glioblastoma, IDH-wildtype	-	-	-
261	Female	25	Left frontal lobe	-5.852373278	-7.684228062	Glioblastoma	IV	Glioblastoma, NOS	NA	-	-
262	Male	42	Right frontal lobe	-12.03827321	-12.30744365	Astrocytoma	II	endroglioma, IDH-mutant and 1p/19q-cc	+	+	-
263	Female	51	Right frontal lobe	-10.11433509	-10.50400629	Astrocytoma	II	Diffuse astrocytoma, IDH-mutant	+	-	-
264	Male	11	Posterior fossa	-5.240260641	-5.207807743	Glioblastoma	IV	Glioblastoma, IDH-wildtype	-	-	-
265	Male	37	frontal lobe	-6.242160818	-5.747731111	Oligoastrocytoma	II	Diffuse astrocytoma, IDH-mutant	+	-	-

266	Female	39	Right frontal lobe	-5.891209119	-3.772550222	Glioblastoma	IV	Glioblastoma, IDH-wildtype	-	-	-
267	Male	22	Right thalamus	-5.158075601	-4.752234831	Oligoastrocytoma	III	Anaplastic astrocytoma, IDH-wildtype	-	-	-
268	Female	14	Right parietal occipital lobe	-5.3094434	-4.376848956	Glioblastoma	IV	Glioblastoma, IDH-wildtype	-	-	-
269	Male	52	Left frontal lobe	-4.540746628	-4.637908376	Glioblastoma	IV	Glioblastoma, IDH-mutant	+	-	-
270	Male	51	Left parietal occipital lobe	-4.48210209	-5.696392591	Glioblastoma	IV	Glioblastoma, IDH-mutant	+	+	+
271	Male	47	Left frontal lobe	-4.670021267	-6.655103864	Oligoastrocytoma	II	Oligodendroglioma, NOS	-	+	-
272	Female	46	Left frontal lobe	-6.021010449	-6.303707325	Oligodendroglioma	II	Oligodendroglioma, NOS	-	+	+
273	Male	45	Left frontal lobe	-3.735715001	-6.985420472	Glioblastoma	IV	Glioblastoma, IDH-wildtype	-	-	-
274	Male	60	Right frontal lobe	-11.06446928	-10.11184195	Oligoastrocytoma	II	endroglioma, IDH-mutant and 1p/19q-cc	+	+	+
275	Male	70	Left frontal lobe	-5.335180479	-3.258805219	Glioblastoma	IV	Glioblastoma, IDH-wildtype	-	-	-
276	Male	23	Right fronto parietal lobe	-6.217214688	-6.264750618	Astrocytoma	II	Diffuse astrocytoma, IDH-wildtype	-	-	-
277	Female	41	Right frontotemporal lobe	-7.759603695	-4.626981453	Oligoastrocytoma	II	Diffuse astrocytoma, IDH-wildtype	-	-	-
278	Male	30	Left frontal lobe	-5.904924436	-3.706648319	Oligoastrocytoma	II	Diffuse astrocytoma, IDH-mutant	+	-	NA
279	Male	39	Left frontotemporal lobe	-5.922500884	-4.56111753	Oligodendroglioma	III	Anaplastic oligodendroglioma, NOS	-	+	+
280	Male	77	Right temporal lobe	-5.564983459	-7.393350617	Glioblastoma	IV	Glioblastoma, IDH-wildtype	-	-	+
281	Female	42	Left temporal lobe	-4.725930278	-6.436838386	Oligodendroglioma	II	Oligodendroglioma, NOS	-	-	-
282	Male	56	Left frontal lobe	-5.407888799	-4.823335731	Astrocytoma	III	Anaplastic astrocytoma, IDH-wildtype	-	-	-
283	Female	57	Right temporal lobe	-7.441516751	-7.594796521	Astrocytoma	II	Diffuse astrocytoma, IDH-wildtype	-	-	-
284	Male	39	Right frontal lobe	-6.333303582	-6.770417236	Astrocytoma	II	Diffuse astrocytoma, IDH-wildtype	-	-	+
285	Male	47	Right frontotemporal lobe	-6.357452733	-3.394642307	Glioblastoma	IV	Glioblastoma, IDH-wildtype	-	-	-
286	Female	38	Right frontal lobe	-7.418297635	-5.465122313	Astrocytoma	II	Diffuse astrocytoma, IDH-mutant	+	-	+
287	Male	20	Right temporal lobe	-8.526201509	-5.21772281	Astrocytoma	II	Diffuse astrocytoma, IDH-wildtype	-	-	NA
288	Female	47	Right lateral ventricle	-8.491853096	-6.602946407	Astrocytoma	II	Diffuse astrocytoma, IDH-wildtype	-	-	-
289	Male	51	Right frontal lobe	-5.049007102	-6.153462573	Glioblastoma	IV	Glioblastoma, IDH-wildtype	-	-	-
290	Female	16	Left cerebellum	-8.691391539	-7.580291294	Astrocytoma	II	Diffuse astrocytoma, IDH-wildtype	-	-	-
291	Female	58	Left temporal lobe	-5.466347625	-3.268543465	Glioblastoma	IV	Glioblastoma, IDH-wildtype	-	-	-
292	Male	57	Right temporal lobe	-4.807289941	-3.797741696	Glioblastoma	IV	Glioblastoma, IDH-wildtype	-	+	-
293	Male	58	Left frontal lobe	-5.321878571	-7.085339924	Oligoastrocytoma	III	Anaplastic astrocytoma, IDH-mutant	+	-	-
294	Male	76	Left parietal lobe	-5.458772034	-4.276210092	Glioblastoma	IV	Glioblastoma, IDH-wildtype	-	-	-
295	Male	71	Right frontal lobe	-5.930441905	-5.871409009	Glioblastoma	IV	Glioblastoma, IDH-wildtype	-	-	-
296	Female	43	Right temporal parietal lobe	-6.72615866	-5.056610086	Astrocytoma	III	Anaplastic astrocytoma, IDH-wildtype	-	-	-
297	Male	35	Right parietal occipital lobe	-9.90518074	-11.44847931	NA	II	NA	-	-	-
298	Male	49	Left frontal lobe	-5.655555797	-5.492944188	Glioblastoma	IV	Glioblastoma, IDH-wildtype	-	-	-
299	Female	34	Right frontal lobe	-5.894048865	-5.914979076	Astrocytoma	II	Diffuse astrocytoma, IDH-mutant	+	-	+
300	Male	46	Left frontal lobe	-5.811524357	-2.718120574	Astrocytoma	III	Anaplastic astrocytoma, IDH-mutant	+	-	-
301	Male	25	Right temporal lobe	-5.905745218	-3.744117919	Astrocytoma	III	Anaplastic astrocytoma, IDH-wildtype	-	-	+
302	Male	61	Left frontal lobe	-4.695680704	-6.049081629	Glioblastoma	IV	Glioblastoma, IDH-wildtype	-	-	-
303	Female	40	Left frontal lobe	-6.665620002	-7.142259768	Astrocytoma	II	Diffuse astrocytoma, IDH-wildtype	-	-	-
304	Female	37	Left frontal lobe	-5.724536942	-5.355889255	NA	III	NA	-	-	-
305	Female	25	Right frontal lobe	-5.784839109	-5.147670734	Oligodendroglioma	III	ligodendroglioma, IDH-mutant and 1p/1	+	+	NA
306	Female	64	Right fronto parietal lobe	-5.293969912	-4.412332661	Glioblastoma	IV	Glioblastoma, IDH-wildtype	-	-	NA
307	Female	33	Left temporal lobe	-6.313625799	-3.526800313	Glioblastoma	IV	Glioblastoma, IDH-wildtype	-	-	NA
308	Female	47	Right parietal occipital lobe	-6.414394516	-4.557606475	Astrocytoma	III	Anaplastic astrocytoma, IDH-wildtype	-	-	NA
309	Female	51	Right temporal parietal lobe	-5.088221526	-7.195890544	Glioblastoma	IV	Glioblastoma, IDH-wildtype	-	-	-
310	Male	59	Left frontal lobe	-5.950362533	-6.653097405	Astrocytoma	II	Diffuse astrocytoma, IDH-wildtype	-	-	NA
311	Male	46	Left frontal lobe	-4.883828351	-9.527128571	Astrocytoma	III	Anaplastic astrocytoma, IDH-mutant	+	+	NA
312	Female	37	Left frontal lobe	-7.217689302	-8.490570515	Oligoastrocytoma	II	NA	+	NA	+
313	Female	44	Left Insular lobe	-6.212496593	-5.042839642	Astrocytoma	II	Diffuse astrocytoma, IDH-wildtype	-	NA	+
314	Female	41	NA	-12.19991729	-10.56709684	Oligodendroglioma	II	Oligodendroglioma, NOS	-	NA	-
315	Male	58	Right parietal lobe	-5.142542027	-7.898504742	Astrocytoma	III	Anaplastic astrocytoma, IDH-wildtype	-	NA	NA
316	Female	37	Left Insular lobe	-6.228975153	-5.026603173	Astrocytoma	III	Anaplastic astrocytoma, IDH-wildtype	-	NA	NA
317	Male	41	Left temporal lobe	-7.432311223	-13.58415952	NA	III	NA	-	-	NA
318	Male	59	Right occipital part	-7.58228882	-6.476964581	Oligodendroglioma	II	Oligodendroglioma, NOS	-	+	+
319	Female	73	Right frontal lobe	-5.443569337	-4.691618396	Glioblastoma	IV	Glioblastoma, IDH-wildtype	-	+	+
320	Male	21	Left frontal lobe	-6.31954685	-5.462433263	Astrocytoma	III	Anaplastic astrocytoma, IDH-wildtype	-	-	-
321	Female	49	Right parietal occipital lobe	-5.714795464	-8.418105115	Astrocytoma	III	Anaplastic astrocytoma, IDH-wildtype	-	-	+
322	Male	40	Bilateral frontal lobe	-10.11271406	-12.51576827	Astrocytoma	II	endroglioma, IDH-mutant and 1p/19q-cc	+	+	+
323	Male	64	Right temporal lobe	-5.89040324	-4.125590805	Glioblastoma	IV	Glioblastoma, IDH-wildtype	-	+	-
324	Female	39	Right insular lobe	-5.890305319	-8.449029902	Astrocytoma	III	Anaplastic astrocytoma, IDH-wildtype	-	-	-
325	Male	58	Right temporal occipital lobe	-5.18045411	-5.251152051	Glioblastoma	IV	Glioblastoma, IDH-wildtype	-	-	+
326	Male	44	Right frontal lobe	-7.093315364	-7.671757807	Astrocytoma	II	Diffuse astrocytoma, IDH-mutant	+	-	-
327	Male	61	Right frontotemporal lobe	-6.456140901	-4.427996886	Glioblastoma	IV	Glioblastoma, IDH-wildtype	-	NA	+
328	Male	46	Right frontal lobe	-5.895292176	-7.756866263	Glioblastoma	IV	Glioblastoma, IDH-wildtype	-	-	-
329	Male	58	Left frontotemporal lobe	-6.049943945	-4.672047593	Oligodendroglioma	III	Anaplastic oligodendroglioma, NOS	-	-	-
330	Male	68	Left parietal lobe	-6.734108971	-4.590445889	Glioblastoma	IV	Glioblastoma, IDH-wildtype	-	-	-
331	Female	27	Right frontal lobe	-6.121879649	-6.945491787	Oligoastrocytoma	III	ligodendroglioma, IDH-mutant and 1p/1	+	+	+
332	Female	49	Right frontal lobe	-6.739146349	-11.50973854	Astrocytoma	III	Anaplastic astrocytoma, IDH-wildtype	-	-	+

333	Male	51	Left frontal lobe	-5.274875983	-3.680471037	Oligoastrocytoma	III	Anaplastic astrocytoma, IDH-wildtype	-	-	+
334	Female	24	spinal cord	-6.833395034	-5.466503558	Oligoastrocytoma	II	Diffuse astrocytoma, IDH-wildtype	-	-	-
335	Male	67	Right temporal lobe	-6.097132169	-5.517712751	Glioblastoma	IV	Glioblastoma, IDH-wildtype	-	-	-
336	Female	12	Pons, medulla oblongata and brainstem	-4.899889669	-4.046833212	Glioblastoma	IV	Glioblastoma, IDH-wildtype	-	-	-
337	Female	59	Right frontotemporal lobe	-6.045466513	-4.499392709	Glioblastoma	IV	Glioblastoma, IDH-wildtype	-	-	+
338	Male	59	Right temporal lobe	-6.276979437	-6.71417292	Glioblastoma	IV	Glioblastoma, IDH-wildtype	-	-	-

Supplementary Table 2: the clinicopathologic mutational and survival information of patients with glioma in TCGA dataset

Sample	Age	Gender	ASCL2 level	ATG9B level	ATG9B CNV	Histology	Grade	Integrated diagonosis	Subtype	Transcriptome.Subtype	survival time(days)	survival time(months)	status(0 survival;1 dead)	IDH.status	Chr.1p_19q.codeletion	MGMT.promoter.status	Chr.7.gain_Chr.10.loss	ATRX.status
TCGA.02.0047	78	Male	3.930879235	5.80488735	diploid	GBM	IV	Glioblastoma, IDH-wildtype	LGm6-GBM	ME	441	14.7	1	WT	non-codel	Unmethylated	No combined CNA	WT
TCGA.02.0055	62	Female	3.74353651	4.05951103	Gain	GBM	IV	Glioblastoma, IDH-wildtype	Mesenchymal-like	ME	75	2.5	1	WT	non-codel	Unmethylated	No combined CNA	WT
TCGA.02.2483	43	Male	4.542059898	4.964828327	diploid	GBM	IV	Glioblastoma, IDH-mutant	G-CIMP-low	PN	459	15.3	0	Mutant	non-codel	Methylated	No combined CNA	Mutant
TCGA.02.2485	53	Male	3.181325221	4.31883019	Gain	GBM	IV	Glioblastoma, IDH-wildtype	Classic-like	CL	462	15.4	0	WT	non-codel	Unmethylated	Gain chr 7 & loss chr 10	WT
TCGA.02.2486	64	Male	5.164540928	3.126262564	Gain	GBM	IV	Glioblastoma, IDH-wildtype	Classic-like	ME	609	20.3	1	WT	non-codel	Unmethylated	Gain chr 7 & loss chr 10	WT
TCGA.06.0125	63	Female	1.558022674	4.899862004	Gain	GBM	IV	Glioblastoma, IDH-wildtype	Classic-like	CL	1428	47.6	1	WT	non-codel	Methylated	No combined CNA	WT
TCGA.06.0129	30	Male	5.382141535	4.40888166	diploid	GBM	IV	Glioblastoma, IDH-mutant	G-CIMP-low	PN	1011	33.7	1	Mutant	non-codel	Methylated	No combined CNA	Mutant
TCGA.06.0130	54	Male	6.244059327	4.302436041	Gain	GBM	IV	Glioblastoma, IDH-wildtype	Mesenchymal-like	ME	387	12.9	1	WT	non-codel	Unmethylated	No combined CNA	WT
TCGA.06.0132	49	Male	5.311208821	5.090455449	Gain	GBM	IV	Glioblastoma, IDH-wildtype		NE	762	25.4	1	WT	non-codel		No combined CNA	WT
TCGA.06.0138	43	Male	5.267149979	6.150259084	Gain	GBM	IV	Glioblastoma, IDH-wildtype		ME	726	24.2	1	WT	non-codel		Gain chr 7 & loss chr 10	
TCGA.06.0141	62	Male	4.496583783	6.601194338	Gain	GBM	IV	Glioblastoma, IDH-wildtype	LGm6-GBM	ME	309	10.3	1	WT	non-codel	Unmethylated	No combined CNA	WT
TCGA.06.0156	57	Male	3.823494625	7.893842705	NA	GBM	IV	Glioblastoma, IDH-mutant		ME	174	5.8	1	Mutant				
TCGA.06.0157	63	Female	4.081936082	4.620955834	Gain	GBM	IV	Glioblastoma, IDH-wildtype		CL	96	3.2	1	WT	non-codel		Gain chr 7 & loss chr 10	WT
TCGA.06.0158	73	Male	4.750767272	4.057736183	diploid	GBM	IV	Glioblastoma, IDH-wildtype		CL	324	10.8	1	WT	non-codel		No combined CNA	WT
TCGA.06.0168	59	Female	5.125308074	4.87657097	Gain	GBM	IV	Glioblastoma, IDH-wildtype		ME	591	19.7	1	WT	non-codel		Gain chr 7 & loss chr 10	WT
TCGA.06.0174	54	Male	2.918386234	6.748592723	Gain	GBM	IV	Glioblastoma, IDH-wildtype		PN	96	3.2	1	WT	non-codel		Gain chr 7 & loss chr 10	WT
TCGA.06.0178	38	Male	4.73545719	4.322779034	diploid	GBM	IV	Glioblastoma, IDH-mutant		PN	2643	88.1	1	Mutant	non-codel		No combined CNA	WT
TCGA.06.0184	63	Male	5.914882589	3.60844261	Gain	GBM	IV	Glioblastoma, IDH-wildtype		ME	2097	69.9	1	WT	non-codel		Gain chr 7 & loss chr 10	WT
TCGA.06.0187	69	Male	3.469898997	5.502212834	Gain	GBM	IV	Glioblastoma, IDH-wildtype		CL	816	27.2	1	WT	non-codel		No combined CNA	
TCGA.06.0190	62	Male	4.810813242	5.983461092	Gain	GBM	IV	Glioblastoma, IDH-wildtype		ME	312	10.4	1	WT	non-codel		Gain chr 7 & loss chr 10	WT
TCGA.06.0210	72	Female	4.508758229	6.550191145	Gain	GBM	IV	Glioblastoma, IDH-wildtype		ME	222	7.4	1	WT	non-codel		Gain chr 7 & loss chr 10	WT
TCGA.06.0211	47	Male	3.895176574	3.59489485	Gain	GBM	IV	Glioblastoma, IDH-wildtype		CL	354	11.8	1	WT	non-codel		Gain chr 7 & loss chr 10	WT
TCGA.06.0219	67	Male	4.327162783	4.824375819	diploid	GBM	IV	Glioblastoma, IDH-wildtype		NE	21	0.7	1	WT	non-codel		No combined CNA	WT
TCGA.06.0238	46	Male	5.060566245	7.16437298	Gain	GBM	IV	Glioblastoma, IDH-wildtype		PN	399	13.3	1	WT	non-codel		Gain chr 7 & loss chr 10	WT
TCGA.06.0644	71	Male	3.421559945	5.236182616	Gain	GBM	IV	Glioblastoma, IDH-wildtype		ME	378	12.6	1	WT	non-codel		Gain chr 7 & loss chr 10	WT
TCGA.06.0645	55	Female	5.129315992	5.121102458	Gain	GBM	IV	Glioblastoma, IDH-wildtype		ME	174	5.8	1	WT	non-codel		Gain chr 7 & loss chr 10	WT
TCGA.06.0646	60	Male	4.759773695	7.189487561	Gain	GBM	IV	Glioblastoma, IDH-wildtype		ME	174	5.8	1	WT	non-codel		Gain chr 7 & loss chr 10	WT
TCGA.06.0649	73	Female	4.79215257	9.478227622	Gain	GBM	IV	Glioblastoma, IDH-wildtype		ME	63	2.1	1	WT	non-codel		Gain chr 7 & loss chr 10	WT
TCGA.06.0686	53	Male	4.099985243	8.067835517	Gain	GBM	IV	Glioblastoma, IDH-wildtype		PN	426	14.2	1	WT	non-codel		Gain chr 7 & loss chr 10	WT
TCGA.06.0743	69	Male	4.291154119	6.49815511	Gain	GBM	IV	Glioblastoma, IDH-wildtype		CL	792	26.4	1	WT	non-codel		Gain chr 7 & loss chr 10	WT
TCGA.06.0744	66	Male	4.752994728	4.62684794	Gain	GBM	IV	Glioblastoma, IDH-wildtype		CL	1407	46.9	1	WT	non-codel		Gain chr 7 & loss chr 10	WT
TCGA.06.0745	59	Male	5.59819546	7.209464088	Gain	GBM	IV	Glioblastoma, IDH-wildtype		ME	237	7.9	1	WT	non-codel		Gain chr 7 & loss chr 10	WT
TCGA.06.0747	53	Male	3.038102511	3.893983426	Gain	GBM	IV	Glioblastoma, IDH-wildtype		CL	81	2.7	1	WT	non-codel		No combined CNA	WT
TCGA.06.0749	50	Male	6.030035854	6.498212565	Gain	GBM	IV	Glioblastoma, IDH-wildtype		NE	81	2.7	1	WT	non-codel		Gain chr 7 & loss chr 10	WT
TCGA.06.0750	43	Male	4.515434926	6.740525557	Gain	GBM	IV	Glioblastoma, IDH-wildtype		ME	27	0.9	1	WT	non-codel		Gain chr 7 & loss chr 10	WT
TCGA.06.0878	74	Male	4.754785976	4.907284878	Gain	GBM	IV	Glioblastoma, IDH-wildtype	Mesenchymal-like	ME	216	7.2	0	WT	non-codel	Unmethylated	Gain chr 7 & loss chr 10	WT
TCGA.06.0882	30	Male	5.302077729	4.639423198	Gain	GBM	IV	Glioblastoma, IDH-wildtype	LGm6-GBM	ME	624	20.8	1	WT	non-codel	Unmethylated	Gain chr 7 & loss chr 10	WT
TCGA.06.1804	81	Female	3.471889774	5.459316854	Gain	GBM	IV	Glioblastoma, IDH-wildtype	Classic-like		408	13.6	1	WT	non-codel	Methylated	Gain chr 7 & loss chr 10	WT
TCGA.06.2557	76	Male	5.489921799	4.213347282	Gain	GBM	IV	Glioblastoma, IDH-wildtype	Classic-like	ME	33	1.1	1	WT	non-codel	Unmethylated	No combined CNA	WT
TCGA.06.2558	75	Female	3.65777718	6.454953962	Gain	GBM	IV	Glioblastoma, IDH-wildtype	Mesenchymal-like	PN	375	12.5	1	WT	non-codel	Unmethylated	Gain chr 7 & loss chr 10	WT
TCGA.06.2559	83	Male	3.925097452	6.779452785	Gain	GBM	IV	Glioblastoma, IDH-wildtype	Mesenchymal-like	ME	147	4.9	1	WT	non-codel	Methylated	No combined CNA	WT
TCGA.06.2561	53	Female	3.973730653	4.711880308	Gain	GBM	IV	Glioblastoma, IDH-wildtype	Mesenchymal-like	ME	528	17.6	1	WT	non-codel	Unmethylated	Gain chr 7 & loss chr 10	WT
TCGA.06.2562	81	Male	4.093357346	6.591064501	Gain	GBM	IV	Glioblastoma, IDH-wildtype	Classic-like	ME	378	12.6	1	WT	non-codel	Unmethylated	Gain chr 7 & loss chr 10	WT
TCGA.06.2563	72	Female	4.089091319	4.018162337	Gain	GBM	IV	Glioblastoma, IDH-wildtype	Classic-like	CL	918	30.6	0	WT	non-codel	Methylated	Gain chr 7 & loss chr 10	WT
TCGA.06.2564	50	Male	4.892629027	4.176242975	Gain	GBM	IV	Glioblastoma, IDH-wildtype	Mesenchymal-like	CL	180	6	0	WT	non-codel	Unmethylated	Gain chr 7 & loss chr 10	WT
TCGA.06.2565	59	Male	3.728475649	4.635882058	diploid	GBM	IV	Glioblastoma, IDH-wildtype	Classic-like	CL	498	16.6	1	WT	non-codel	Methylated	No combined CNA	WT
TCGA.06.2567	65	Male	4.918977694	4.393155317	Gain	GBM	IV	Glioblastoma, IDH-wildtype	Mesenchymal-like	ME	132	4.4	1	WT	non-codel	Methylated	Gain chr 7 & loss chr 10	WT
TCGA.06.2569	24	Female	1.255742421	2.837014776	NA	GBM	IV	Glioblastoma, IDH-wildtype	LGm6-GBM		12	0.4	0	WT	non-codel	Unmethylated	No combined CNA	WT
TCGA.06.2570	21	Female	4.451026435	5.523819109	diploid	GBM	IV	Glioblastoma, IDH-mutant	G-CIMP-low	PN	945	31.5	0	Mutant	non-codel	Methylated	No combined CNA	Mutant
TCGA.06.5408	54	Female	3.635417762	6.400087017	Gain	GBM	IV	Glioblastoma, IDH-wildtype	Mesenchymal-like	CL	351	11.7	1	WT	non-codel	Unmethylated	Gain chr 7 & loss chr 10	WT
TCGA.06.5410	72	Female	5.389621875	4.083732475	Gain	GBM	IV	Glioblastoma, IDH-wildtype	LGm6-GBM	ME	105	3.5	1	WT	non-codel	Methylated	No combined CNA	WT
TCGA.06.5411	51	Male	3.209094597	6.697102403	Gain	GBM	IV	Glioblastoma, IDH-wildtype	Mesenchymal-like	NE	249	8.3	1	WT	non-codel	Unmethylated	Gain chr 7 & loss chr 10	WT
TCGA.06.5412	78	Female	4.810288852	5.412734112	Gain	GBM	IV	Glioblastoma, IDH-wildtype	Mesenchymal-like	ME	135	4.5	1	WT	non-codel	Methylated	No combined CNA	WT
TCGA.06.5413	67	Male	3.500598124	7.996089849	Gain	GBM	IV	Glioblastoma, IDH-wildtype	Classic-like	ME	264	8.8	0	WT	non-codel	Unmethylated	Gain chr 7 & loss chr 10	WT
TCGA.06.5414	61	Male	3.706408988	1.780100084	Gain	GBM	IV	Glioblastoma, IDH-wildtype	Classic-like	CL	270	9	0	WT	non-codel	Unmethylated	Gain chr 7 & loss chr 10	WT
TCGA.06.5415	60	Male	3.608915667	5.094058678	Gain	GBM	IV	Glioblastoma, IDH-wildtype	Classic-like	CL	258	8.6	0	WT	non-codel	Unmethylated	Gain chr 7 & loss chr 10	WT
TCGA.06.5416	23	Female	-1	7.644830401	diploid	GBM	IV	Glioblastoma, NOS		PN	201	6.7	0		non-codel	Unmethylated	No combined CNA	
TCGA.06.5417	45	Female	1.268075023	2.109962253	NA	GBM	IV	Glioblastoma, IDH-mutant			153	5.1	0	Mutant		Methylated		Mutant
TCGA.06.5418	75	Female	3.74453787	5.911418355	Gain	GBM	IV	Glioblastoma, IDH-wildtype	Mesenchymal-like	ME	81	2.7	1	WT	non-codel	Unmethylated	Gain chr 7 & loss chr 10	WT
TCGA.06.5856	58	Male	4.076011739	5.920338562	Gain	GBM	IV	Glioblastoma, IDH-wildtype	Classic-like	CL	114	3.8	1	WT	non-codel	Unmethylated	Gain chr 7 & loss chr 10	WT
TCGA.06.5858	45	Female	4.596994757	5.644606196	Gain	GBM	IV	Glioblastoma, IDH-wildtype	LGm6-GBM	ME	186	6.2	0	WT	non-codel	Unmethylated	No combined CNA	Mutant
TCGA.06.5859	63	Male	4.195922357	6.685724345	Gain	GBM	IV	Glioblastoma, IDH-wildtype	Classic-like	ME	138	4.6	0	WT	non-codel	Unmethylated	Gain chr 7 & loss chr 10	WT
TCGA.08.0386	74	Male	2.785822535	7.159102367	Gain	GBM	IV	Glioblastoma, IDH-wildtype			540	18	1	WT	non-codel		Gain chr 7 & loss chr 10	WT
TCGA.12.0616	36	Female	4.440553608	5.798257933	Gain	GBM	IV	Glioblastoma, IDH-wildtype		PN	441	14.7	1	WT	non-codel		No combined CNA	WT
TCGA.12.0618	49	Male	2.762221232	8.039209693	Gain	GBM	IV	Glioblastoma, IDH-wildtype		PN	390	13	1	WT	non-codel		Gain chr 7 & loss chr 10	WT
TCGA.12.0619	60	Male	4.156064869	7.255182512	Gain	GBM	IV	Glioblastoma, IDH-wildtype		ME	1047	34.9	1	WT	non-codel		Gain chr 7 & loss chr 10	WT
TCGA.12.0821	62	Male	3.246225649	6.737159443	Gain	GBM	IV	Glioblastoma, IDH-wildtype	LGm6-GBM		318	10.6	1	WT	non-codel	Unmethylated	Gain chr 7 & loss chr 10	WT
TCGA.12.1597	62	Female	4.423584894	5.859283807	NA	GBM	IV	Glioblastoma, IDH-wildtype	Classic-like	PN	666	22.2	1	WT		Unmethylated		WT
TCGA.12.3650	46	Male	4.139412192	5.650118303	Gain	GBM	IV	Glioblastoma, IDH-wildtype	Classic-like	CL	327	10.9	1	WT	non-codel	Unmethylated	Gain chr 7 & loss chr 10	WT
TCGA.12.3652	60	Male	3.43065995	4.869891142	diploid	GBM	IV	Glioblastoma, IDH-wildtype	Classic-like	CL	1047	34.9	1	WT	non-codel	Unmethylated	No combined CNA	WT
TCGA.12.3653	34	Female	4.522313171	3.253308589	Gain	GBM	IV	Glioblastoma, IDH-wildtype	Mesenchymal-like	CL	435	14.5	1	WT	non-codel	Unmethylated	Gain chr 7 & loss chr 10	WT
TCGA.12.5295	60	Female	4.642054582	3.441031903	Gain	GBM	IV	Glioblastoma, IDH-wildtype	Classic-like	ME	447	14.9	1	WT	non-codel	Methylated	Gain chr 7 & loss chr 10	WT
TCGA.12.5299	56	Female	4.172839506	5.187320398	Gain	GBM	IV	Glioblastoma, IDH-wildtype	Mesenchymal-like	CL	96	3.2	1	WT	non-codel	Unmethylated	Gain chr 7 & loss chr 10	WT
TCGA.14.0781	49	Male	4.573004837	5.101292888	Gain	GBM	IV	Glioblastoma, IDH-wildtype	Mesenchymal-like	ME	30	1	1	WT	non-codel	Unmethylated	No combined CNA	WT
TCGA.14.0787	69	Male	4.22050022	4.328147114	Gain	GBM	IV	Glioblastoma, IDH-wildtype	Classic-like	CL	66	2.2	1	WT	non-codel	Methylated	Gain chr 7 & loss chr 10	WT
TCGA.14.0789	54	Male	4.916022745	5.575197346	Gain	GBM	IV	Glioblastoma, IDH-wildtype	Classic-like	ME	336	11.2	1	WT	non-codel	Methylated	Gain chr 7 & loss chr 10	WT
TCGA.14.0790	64	Female	2.731900066	3.041558406	Gain	GBM	IV	Glioblastoma, IDH-wildtype	Classic-like	CL	414	13.8	1	WT	non-codel	Methylated	Gain chr 7 & loss chr 10	WT
TCGA.14.0817	69	Female	5.974233309	6.135920609	Gain	GBM	IV	Glioblastoma, IDH-wildtype	Classic-like	ME	162	5.4	1	WT	non-codel	Unmethylated	Gain chr 7 & loss chr 10	WT
TCGA.14.1034	60	Female	4.170253578	3.050728053	Gain	GBM	IV	Glioblastoma, IDH-wildtype	Classic-like	ME	477	15.9	1	WT	non-codel	Methylated	Gain chr 7 & loss chr 10	WT
TCGA.14.1823	58	Female	5.219524793	4.150096575	Gain	GBM	IV	Glioblastoma, IDH-wildtype	Classic-like	ME	537	17.9	1	WT	non-codel	Methylated	Gain chr 7 & loss chr 10	WT
TCGA.14.1825	70	Male	3.396899284	5.775601063	NA	GBM	IV	Glioblastoma, IDH-wildtype	Mesenchymal-like	PN	228	7.6	1	WT	non-codel	Unmethylated	Gain chr 7 & loss chr 10	WT
TCGA.14.1829	57	Male	3.210123868	7.689974916	Gain	GBM	IV	Glioblastoma, IDH-wildtype	Mesenchymal-like	ME	216	7.2	0	WT	non-codel	Unmethylated	Gain chr 7 & loss chr 10	WT
TCGA.14.2554	52	Female	4.970770844	3.668243959	Gain	GBM	IV	Glioblastoma, IDH-wildtype	Mesenchymal-like	ME	525	17.5	1	WT	non-codel	Unmethylated	Gain chr 7 & loss chr 10	WT
TCGA.15.0742	65	Male	3.714970805	4.440321047	Gain	GBM	IV	Glioblastoma, IDH-wildtype		CL	414	13.8	1	WT	non-codel		Gain chr 7 & loss chr 10	WT
TCGA.15.1444	21	Male	4.497101361	2.761732057	diploid	GBM	IV	Glioblastoma, IDH-mutant	G-CIMP-high		1515	50.5	1	Mutant	non-codel	Methylated	No combined CNA	Mutant
TCGA.16.0846	85	Male	5.718334224	5.994466585	Gain	GBM	IV	Glioblastoma, IDH-wildtype	Classic-like		117	3.9	1	WT	non-codel	Methylated	Gain chr 7 & loss chr 10	WT

TCGA.16.1045	49	Female	5.119684025	4.88462234	Gain	GBM	IV	Glioblastoma, IDH-wildtype	Mesenchymal-like	ME	870	29	1	WT	non-codel	Methylated	Gain chr 7 & loss chr 10	WT
TCGA.19.1390	63	Female	3.301880253	7.369502254	Gain	GBM	IV	Glioblastoma, IDH-wildtype	Classic-like	PN	762	25.4	1	WT	non-codel	Methylated	No combined CNA	WT
TCGA.19.1787	48	Male	5.14501301	2.361263047	Gain	GBM	IV	Glioblastoma, NOS		ME	381	12.7	1		non-codel	Methylated	Gain chr 7 & loss chr 10	
TCGA.19.2619	55	Female	2.560397037	5.042215317	Gain	GBM	IV	Glioblastoma, IDH-wildtype	Mesenchymal-like	CL	291	9.7	0	WT	non-codel	Methylated	Gain chr 7 & loss chr 10	WT
TCGA.19.2620	70	Male	3.271649772	4.408834118	Gain	GBM	IV	Glioblastoma, IDH-wildtype	Classic-like	CL	147	4.9	1	WT	non-codel	Methylated	No combined CNA	WT
TCGA.19.2624	51	Male	2.074950644	7.166912236	Gain	GBM	IV	Glioblastoma, IDH-wildtype	Mesenchymal-like	CL	6	0.2	1	WT	non-codel	Unmethylated	Gain chr 7 & loss chr 10	WT
TCGA.19.2625	76	Female	4.355749269	5.55095298	diploid	GBM	IV	Glioblastoma, IDH-wildtype	Mesenchymal-like	ME	123	4.1	1	WT	non-codel	Unmethylated	No combined CNA	WT
TCGA.19.2629	60	Male	4.987207156	2.171270889	Gain	GBM	IV	Glioblastoma, IDH-mutant	G-CIMP-low		726	24.2	1	Mutant	non-codel	Unmethylated	No combined CNA	Mutant
TCGA.19.4065	36	Male	4.176266915	6.055451644	Gain	GBM	IV	Glioblastoma, NOS		ME	210	7	0		non-codel	Unmethylated	Gain chr 7 & loss chr 10	
TCGA.19.5960	56	Male	3.129794053	4.821051976	Gain	GBM	IV	Glioblastoma, IDH-wildtype	Classic-like	CL	450	15	1	WT	non-codel	Unmethylated	Gain chr 7 & loss chr 10	WT
TCGA.26.1442	43	Male	2.971111255	4.381705597	Gain	GBM	IV	Glioblastoma, IDH-mutant	G-CIMP-high		939	31.3	0	Mutant	non-codel	Methylated	No combined CNA	WT
TCGA.26.5132	74	Male	4.042189046	5.119908081	Gain	GBM	IV	Glioblastoma, IDH-wildtype	Classic-like	ME	282	9.4	0	WT	non-codel	Methylated	Gain chr 7 & loss chr 10	WT
TCGA.26.5133	59	Male	2.911269736	2.400674542	diploid	GBM	IV	Glioblastoma, IDH-wildtype	LGm6-GBM		447	14.9	0	WT	non-codel	Unmethylated	No combined CNA	WT
TCGA.26.5134	74	Male	3.891837158	5.908073121	diploid	GBM	IV	Glioblastoma, IDH-wildtype	Mesenchymal-like		165	5.5	0	WT	non-codel	Unmethylated	No combined CNA	WT
TCGA.26.5135	72	Female	4.187569822	6.62223782	Gain	GBM	IV	Glioblastoma, IDH-wildtype	Mesenchymal-like	ME	267	8.9	1	WT	non-codel	Methylated	Gain chr 7 & loss chr 10	WT
TCGA.26.5136	78	Female	5.417255065	6.310500088	Gain	GBM	IV	Glioblastoma, IDH-wildtype	Mesenchymal-like	ME	570	19	1	WT	non-codel	Methylated	Gain chr 7 & loss chr 10	WT
TCGA.26.5139	65	Female	3.564841513	6.175911769	Gain	GBM	IV	Glioblastoma, IDH-wildtype	Mesenchymal-like	CL	48	1.6	0	WT	non-codel	Unmethylated	Gain chr 7 & loss chr 10	WT
TCGA.27.1830	57	Male	3.811645705	6.528616637	Gain	GBM	IV	Glioblastoma, IDH-wildtype	Mesenchymal-like	ME	153	5.1	1	WT	non-codel	Unmethylated	Gain chr 7 & loss chr 10	WT
TCGA.27.1831	66	Male	3.860893293	4.477573719	Gain	GBM	IV	Glioblastoma, IDH-wildtype	Mesenchymal-like	ME	498	16.6	1	WT	non-codel	Unmethylated	Gain chr 7 & loss chr 10	WT
TCGA.27.1832	59	Female	5.005103513	7.028342608	Gain	GBM	IV	Glioblastoma, IDH-wildtype	Mesenchymal-like	ME	297	9.9	1	WT	non-codel	Unmethylated	Gain chr 7 & loss chr 10	WT
TCGA.27.1834	56	Male	4.92680122	7.761210796	Gain	GBM	IV	Glioblastoma, IDH-wildtype	Mesenchymal-like		1215	40.5	1	WT	non-codel	Methylated	Gain chr 7 & loss chr 10	WT
TCGA.27.1835	53	Female	2.975941948	4.526813761	Gain	GBM	IV	Glioblastoma, IDH-wildtype	Mesenchymal-like	CL	639	21.3	1	WT	non-codel	Methylated	Gain chr 7 & loss chr 10	WT
TCGA.27.1837	36	Male	4.436401735	5.297371192	Gain	GBM	IV	Glioblastoma, IDH-wildtype	Mesenchymal-like	CL	420	14	1	WT	non-codel	Methylated	No combined CNA	WT
TCGA.27.2519	48	Male	4.147534646	8.817570498	Gain	GBM	IV	Glioblastoma, IDH-wildtype	Mesenchymal-like	ME	543	18.1	1	WT	non-codel	Unmethylated	Gain chr 7 & loss chr 10	WT
TCGA.27.2521	34	Male	2.418540795	3.575820573	Gain	GBM	IV	Glioblastoma, IDH-mutant	G-CIMP-low	PN	312	10.4	0	Mutant	non-codel	Methylated	No combined CNA	Mutant
TCGA.27.2523	63	Male	3.194228919	5.547372707	Gain	GBM	IV	Glioblastoma, IDH-wildtype	Classic-like	CL	483	16.1	1	WT	non-codel	Methylated	Gain chr 7 & loss chr 10	WT
TCGA.27.2524	56	Male	4.870206878	5.995957048	Gain	GBM	IV	Glioblastoma, IDH-wildtype	Mesenchymal-like	ME	228	7.6	1	WT	non-codel	Unmethylated	Gain chr 7 & loss chr 10	WT
TCGA.27.2526	79	Female	4.893046647	3.746452514	Gain	GBM	IV	Glioblastoma, IDH-wildtype	Mesenchymal-like	CL	87	2.9	1	WT	non-codel	Unmethylated	No combined CNA	WT
TCGA.27.2528	62	Male	1.265376883	10.30281151	Gain	GBM	IV	Glioblastoma, IDH-wildtype	Classic-like	CL	474	15.8	1	WT	non-codel	Methylated	No combined CNA	WT
TCGA.28.1747	44	Male	4.989552315	4.022962525	Gain	GBM	IV	Glioblastoma, IDH-wildtype	Mesenchymal-like	CL	75	2.5	1	WT	non-codel	Methylated	No combined CNA	WT
TCGA.28.1753	53	Male	4.141514537	5.347467237	Gain	GBM	IV	Glioblastoma, IDH-wildtype	Mesenchymal-like	ME	36	1.2	0	WT	non-codel	Unmethylated	Gain chr 7 & loss chr 10	WT
TCGA.28.2499	59	Male	3.591057019	4.151046991	NA	GBM	IV	Glioblastoma, IDH-wildtype	Classic-like	CL	93	3.1	0	WT		Unmethylated		WT
TCGA.28.2509	77	Female	4.04719809	6.636595621	Gain	GBM	IV	Glioblastoma, IDH-wildtype	Classic-like	ME	144	4.8	0	WT	non-codel	Methylated	Gain chr 7 & loss chr 10	WT
TCGA.28.2510			3.097593943	6.250400753	Gain	GBM	IV	Glioblastoma, IDH-wildtype	LGm6-GBM	NE		0		WT	non-codel	Unmethylated	No combined CNA	WT
TCGA.28.2513	69	Female	4.641933239	5.708347281	Gain	GBM	IV	Glioblastoma, IDH-wildtype	Mesenchymal-like	ME	219	7.3	0	WT	non-codel	Unmethylated	Gain chr 7 & loss chr 10	WT
TCGA.28.2514	45	Male	3.210045919	7.185743673	diploid	GBM	IV	Glioblastoma, IDH-wildtype	Mesenchymal-like	CL	159	5.3	0	WT	non-codel	Unmethylated	No combined CNA	WT
TCGA.28.5204	72	Male	3.993737756	5.479676807	Gain	GBM	IV	Glioblastoma, IDH-wildtype	Classic-like	CL	447	14.9	1	WT	non-codel	Unmethylated	Gain chr 7 & loss chr 10	WT
TCGA.28.5207	71	Male	3.630020981	4.793287263	Gain	GBM	IV	Glioblastoma, IDH-wildtype	Mesenchymal-like	CL	339	11.3	1	WT	non-codel	Unmethylated	Gain chr 7 & loss chr 10	WT
TCGA.28.5208	52	Male	3.984197409	3.464825073	diploid	GBM	IV	Glioblastoma, IDH-wildtype	Classic-like	CL	537	17.9	1	WT	non-codel	Methylated	No combined CNA	WT
TCGA.28.5209	66	Female	3.300123725	4.74990428	Gain	GBM	IV	Glioblastoma, IDH-wildtype	Classic-like	CL	75	2.5	0	WT	non-codel	Methylated	Gain chr 7 & loss chr 10	WT
TCGA.28.5213	72	Male	5.220511828	4.652245548	Gain	GBM	IV	Glioblastoma, IDH-wildtype	Mesenchymal-like	ME	939	31.3	0	WT	non-codel	Unmethylated	Gain chr 7 & loss chr 10	WT
TCGA.28.5215	62	Female	3.438465858	6.614915929	Gain	GBM	IV	Glioblastoma, IDH-wildtype	Mesenchymal-like	PN	330	11	1	WT	non-codel	Methylated	No combined CNA	WT
TCGA.28.5216	52	Male	3.985664364	6.875772692	diploid	GBM	IV	Glioblastoma, IDH-wildtype	Mesenchymal-like	ME	408	13.6	0	WT	non-codel	Unmethylated	No combined CNA	WT
TCGA.28.5218	63	Male	5.469934803	4.688605606	Gain	GBM	IV	Glioblastoma, IDH-wildtype	LGm6-GBM	ME	156	5.2	1	WT	non-codel	Unmethylated	No combined CNA	WT
TCGA.28.5220	67	Male	4.744198777	7.250402648	Gain	GBM	IV	Glioblastoma, IDH-wildtype	Mesenchymal-like		315	10.5	0	WT	non-codel	Unmethylated	Gain chr 7 & loss chr 10	WT
TCGA.32.1970	59	Male	3.778450407	4.465426006	Gain	GBM	IV	Glioblastoma, IDH-wildtype	Mesenchymal-like	CL	462	15.4	1	WT	non-codel	Unmethylated	Gain chr 7 & loss chr 10	WT
TCGA.32.1980	72	Male	4.916987707	6.277871335	Gain	GBM	IV	Glioblastoma, IDH-wildtype	LGm6-GBM	NE	36	1.2	1	WT	non-codel	Unmethylated	No combined CNA	WT
TCGA.32.1982	76	Female	4.377040434	7.067568809	Gain	GBM	IV	Glioblastoma, IDH-wildtype	Classic-like	ME	141	4.7	1	WT	non-codel	Methylated	Gain chr 7 & loss chr 10	WT
TCGA.32.2615	62	Male	3.96521171	6.066763009	diploid	GBM	IV	Glioblastoma, IDH-wildtype	Mesenchymal-like	ME	477	15.9	1	WT	non-codel	Unmethylated	No combined CNA	WT
TCGA.32.2616	48	Female	4.520019872	6.436701549	Gain	GBM	IV	Glioblastoma, NOS		ME	222	7.4	1		non-codel	Methylated	No combined CNA	
TCGA.32.2632	80	Male	5.292218547	4.913775035	Gain	GBM	IV	Glioblastoma, IDH-wildtype	Mesenchymal-like	ME	264	8.8	1	WT	non-codel	Unmethylated	No combined CNA	WT
TCGA.32.2634	82	Male	1.970780046	6.043066667	diploid	GBM	IV	Glioblastoma, IDH-wildtype	Classic-like		684	22.8	0	WT	non-codel	Methylated	No combined CNA	WT
TCGA.32.2638	67	Male	3.665108479	4.230694897	Gain	GBM	IV	Glioblastoma, IDH-wildtype	Classic-like	CL	756	25.2	1	WT	non-codel	Methylated	Gain chr 7 & loss chr 10	WT
TCGA.32.4213	47	Female	5.454722066	5.309136161	diploid	GBM	IV	Glioblastoma, IDH-wildtype	Mesenchymal-like	ME	597	19.9	0	WT	non-codel	Methylated	No combined CNA	WT
TCGA.32.5222	66	Male	3.865661521	4.021177654	Gain	GBM	IV	Glioblastoma, IDH-wildtype	Classic-like	CL	576	19.2	1	WT	non-codel	Methylated	Gain chr 7 & loss chr 10	WT
TCGA.41.2571	89	Male	3.222201767	9.177107543	Gain	GBM	IV	Glioblastoma, IDH-wildtype	Mesenchymal-like	PN	24	0.8	1	WT	non-codel	Unmethylated	Gain chr 7 & loss chr 10	WT
TCGA.41.2572	67	Male	4.836343284	3.728225294	Gain	GBM	IV	Glioblastoma, IDH-wildtype	Classic-like	CL	399	13.3	1	WT	non-codel	Unmethylated	Gain chr 7 & loss chr 10	WT
TCGA.41.3915	48	Male	4.881933754	7.463212096	Gain	GBM	IV	Glioblastoma, IDH-wildtype	Mesenchymal-like	ME	354	11.8	1	WT	non-codel	Methylated	Gain chr 7 & loss chr 10	WT
TCGA.41.4097	63	Female	5.251927341	5.089074365	diploid	GBM	IV	Glioblastoma, IDH-wildtype	Classic-like	ME	6	0.2	1	WT	non-codel	Unmethylated	No combined CNA	WT
TCGA.41.5651	59	Female	2.25286976	6.821000937	Gain	GBM	IV	Glioblastoma, IDH-wildtype	Classic-like	PN	453	15.1	1	WT	non-codel	Methylated	Gain chr 7 & loss chr 10	WT
TCGA.76.4925	76	Male	2.382667253	6.991386924	Gain	GBM	IV	Glioblastoma, IDH-wildtype	Classic-like	CL	144	4.8	1	WT	non-codel	Methylated	Gain chr 7 & loss chr 10	WT
TCGA.76.4926	68	Male	3.239611894	6.201039803	Gain	GBM	IV	Glioblastoma, IDH-wildtype	Mesenchymal-like	CL	135	4.5	1	WT	non-codel	Unmethylated	Gain chr 7 & loss chr 10	WT
TCGA.76.4927	58	Male	4.418426104	6.755129267	NA	GBM	IV	Glioblastoma, IDH-wildtype	Classic-like	CL	528	17.6	1	WT		Unmethylated		WT
TCGA.76.4928	85	Female	4.206854175	4.909019404	Gain	GBM	IV	Glioblastoma, IDH-wildtype	Mesenchymal-like	ME	93	3.1	1	WT	non-codel	Methylated	Gain chr 7 & loss chr 10	WT
TCGA.76.4929	76	Female	4.284203174	8.332625594	Gain	GBM	IV	Glioblastoma, IDH-wildtype	Classic-like		108	3.6	1	WT	non-codel	Methylated	Gain chr 7 & loss chr 10	WT
TCGA.76.4931	70	Female	3.440008691	6.164279444	Gain	GBM	IV	Glioblastoma, IDH-wildtype	Mesenchymal-like	CL	276	9.2	1	WT	non-codel	Unmethylated	Gain chr 7 & loss chr 10	WT
TCGA.76.4932	50	Female	4.636804402	4.365580566	NA	GBM	IV	Glioblastoma, IDH-wildtype	Classic-like	CL	1437	47.9	1	WT		Methylated		WT
TCGA.CS.4938	31	Female	3.009329255	3.700262145	Gain	Astrocytoma	II	Diffuse astrocytoma, IDH-mutant	G-CIMP-high		141	4.7	0	Mutant	non-codel	Unmethylated	No combined CNA	Mutant
TCGA.CS.4941	67	Male	4.931716146	7.595150045	Gain	Astrocytoma	III	Anaplastic astrocytoma, IDH-wildtype	Mesenchymal-like	CL	231	7.7	1	WT	non-codel	Methylated	Gain chr 7 & loss chr 10	WT
TCGA.CS.4942	44	Female	4.037118612	2.619905955	Gain	Astrocytoma	III	Anaplastic astrocytoma, IDH-mutant	G-CIMP-high	PN	1317	43.9	1	Mutant	non-codel	Unmethylated	No combined CNA	Mutant
TCGA.CS.4943	37	Male	3.534372924	2.459090578	diploid	Astrocytoma	III	Anaplastic astrocytoma, IDH-mutant	G-CIMP-high	PN	543	18.1	0	Mutant	non-codel	Methylated	No combined CNA	Mutant
TCGA.CS.4944	50	Male	4.404521695	2.85259817	diploid	Astrocytoma	II	Diffuse astrocytoma, IDH-mutant	G-CIMP-high		318	10.6	0	Mutant	non-codel	Methylated	No combined CNA	WT
TCGA.CS.5390	47	Female	0.299010149	2.576763978	diploid	Oligodendroglioma	II	Oligodendroglioma, IDH-mutant and 1p/19	Codel	PN	1938	64.6	0	Mutant	codel	Methylated	No combined CNA	WT
TCGA.CS.5393	39	Male	3.22901873	4.467670755	diploid	Astrocytoma	III	Anaplastic astrocytoma, IDH-mutant	G-CIMP-high	PN	1206	40.2	0	Mutant	non-codel	Methylated	No combined CNA	WT
TCGA.CS.5394	40	Male	0.588516767	3.508834274	diploid	Astrocytoma	III	Anaplastic astrocytoma, IDH-mutant	Codel	PN	3	0.1	0	Mutant	non-codel	Methylated	No combined CNA	WT
TCGA.CS.5395	43	Male	2.305416093	3.912151637	Gain	Oligodendroglioma	II	Oligodendroglioma, NOS	Mesenchymal-like	CL	630	21	1	WT	non-codel	Unmethylated	Gain chr 7 & loss chr 10	WT
TCGA.CS.5396	53	Female	2.350949786	2.546956178	Gain	Oligodendroglioma	III	Anaplastic oligodendroglioma, IDH-mutant	Codel	PN	300	10	0	Mutant	codel	Methylated	No combined CNA	WT
TCGA.CS.5397	54	Female	2.980482356	5.276850224	Gain	Astrocytoma	III	Anaplastic astrocytoma, IDH-wildtype	Mesenchymal-like	NE	192	6.4	1	WT	non-codel	Unmethylated	Gain chr 7 & loss chr 10	WT
TCGA.CS.6186	58	Male	4.234823178	-1	Gain	Oligoastrocytoma	III	Anaplastic astrocytoma, IDH-wildtype	Classic-like	CL	531	17.7	1	WT	non-codel	Unmethylated	Gain chr 7 & loss chr 10	WT
TCGA.CS.6188	48	Male	3.971764252	3.378844514	Gain	Astrocytoma	III	Anaplastic astrocytoma, IDH-wildtype	Mesenchymal-like	CL	714	23.8	0	WT	non-codel	Unmethylated	Gain chr 7 & loss chr 10	WT
TCGA.CS.6290	31	Male	4.462942273	2.501872207	diploid	Astrocytoma	III	Anaplastic astrocytoma, IDH-mutant	G-CIMP-high		537	17.9	0	Mutant	non-codel	Methylated	No combined CNA	WT
TCGA.CS.6665	51	Female	3.165606489	5.002432496	Gain	Astrocytoma	III	Anaplastic astrocytoma, IDH-mutant	G-CIMP-low		372	12.4	0	Mutant	non-codel	Methylated	No combined CNA	WT
TCGA.CS.6666	22	Male	1.763539053	2.847255331	diploid	Astrocytoma	III	Anaplastic astrocytoma, IDH-mutant	G-CIMP-high	PN	1410	47	0	Mutant	non-codel	Methylated	No combined CNA	Mutant
TCGA.CS.6667	39	Female	2.015747322	3.093830572	diploid	Astrocytoma	II	Diffuse astrocytoma, IDH-mutant	G-CIMP-high		228	7.6	0	Mutant	non-codel	Methylated	No combined CNA	WT
TCGA.CS.6668	57	Female	1.69014887	3.670942282	diploid	Oligodendroglioma	II	Oligodendroglioma, IDH-mutant and 1p/19	Codel	PN	1497	49.9	0	Mutant	codel	Methylated	No combined CNA	WT
TCGA.CS.6669	26	Female	4.359471974	6.697662633	diploid	Oligodendroglioma	II	Oligodendroglioma, NOS	PA-like	NE	1473	49.1	0	WT	non-codel	Unmethylated	No combined CNA	WT
TCGA.CS.6670	43	Male	1.646439186	4.285705523	Gain	Oligodendroglioma	III	Anaplastic oligodendroglioma, IDH-mutant	Codel	PN	198	6.6	0	Mutant	codel	Methylated	No combined CNA	WT
TCGA.DB.5270	38	Female	3.118143737	5.362743393	Gain	Oligoastrocytoma	III	Anaplastic astrocytoma, IDH-mutant	G-CIMP-high	NE	3681	122.7	0	Mutant	non-codel	Methylated	No combined CNA	Mutant

TCGA.DB.5273	33	Male	4.162347015	1.517124486	diploid	Astrocytoma	III	Anaplastic astrocytoma, IDH-mutant	G-CIMP-high		2460	82	0	Mutant	non-codel	Unmethylated	No combined CNA	Mutant
TCGA.DB.5274	37	Female	3.651797967	3.721339846	diploid	Oligoastrocytoma	III	Anaplastic oligodendroglioma, IDH-mutant	Codel	PN	2256	75.2	0	Mutant	codel	Methylated	No combined CNA	WT
TCGA.DB.5275	36	Male	1.938285792	3.386921282	diploid	Oligoastrocytoma	III	Anaplastic astrocytoma, IDH-mutant	G-CIMP-high	PN	1437	47.9	0	Mutant	non-codel	Methylated	No combined CNA	Mutant
TCGA.DB.5276	32	Male	3.667358536	3.899958651	diploid	Oligoastrocytoma	III	Anaplastic astrocytoma, IDH-mutant	G-CIMP-high	NE	2187	72.9	0	Mutant	non-codel	Methylated	No combined CNA	Mutant
TCGA.DB.5277	34	Male	2.527320608	4.84029705	Gain	Astrocytoma	III	Anaplastic astrocytoma, IDH-mutant	G-CIMP-high	PN	1527	50.9	1	Mutant	non-codel	Methylated	No combined CNA	WT
TCGA.DB.5278	17	Male	1.966799585	5.498193414	diploid	Oligodendroglioma	II	Oligodendroglioma, IDH-mutant and 1p/19	Codel	PN	2733	91.1	0	Mutant	codel	Methylated	No combined CNA	WT
TCGA.DB.5279	59	Male	2.145807862	3.633861281	diploid	Oligodendroglioma	II	Oligodendroglioma, IDH-mutant and 1p/19	Codel	NE	1335	44.5	0	Mutant	codel	Methylated	No combined CNA	WT
TCGA.DB.5280	43	Male	2.61223453	4.826248477	diploid	Oligoastrocytoma	II	Diffuse astrocytoma, IDH-mutant	G-CIMP-high	PN	1098	36.6	0	Mutant	non-codel	Methylated	No combined CNA	Mutant
TCGA.DB.5281	61	Male	2.298365055	4.54620921	Gain	Oligoastrocytoma	III	Anaplastic astrocytoma, IDH-mutant	G-CIMP-high	NE	2349	78.3	0	Mutant	non-codel	Methylated	No combined CNA	Mutant
TCGA.DB.A4X9	33	Female	1.859929804	4.770120736	Gain	Oligoastrocytoma	II	Diffuse astrocytoma, IDH-mutant	G-CIMP-high	PN	1392	46.4	0	Mutant	non-codel	Methylated	No combined CNA	WT
TCGA.DB.A4XA	30	Male	3.040980077	3.564475727	diploid	Oligoastrocytoma	II	Oligodendroglioma, IDH-mutant and 1p/19	Codel	PN	564	18.8	0	Mutant	codel	Methylated	No combined CNA	WT
TCGA.DB.A4XB	38	Male	2.086715841	4.701698752	Gain	Astrocytoma	III	Anaplastic astrocytoma, IDH-mutant	G-CIMP-high	PN	906	30.2	0	Mutant	non-codel	Methylated	No combined CNA	WT
TCGA.DB.A4XC	26	Male	3.136864006	4.293738322	diploid	Oligoastrocytoma	II	Diffuse astrocytoma, IDH-mutant	G-CIMP-high	NE	1401	46.7	0	Mutant	non-codel	Methylated	No combined CNA	Mutant
TCGA.DB.A4XD	32	Male	2.263665447	3.52883383	Gain	Astrocytoma	III	Anaplastic astrocytoma, IDH-mutant	G-CIMP-high	PN	1194	39.8	0	Mutant	non-codel	Methylated	No combined CNA	Mutant
TCGA.DB.A4XE	27	Female	2.206736984	4.312483664	Gain	Oligoastrocytoma	III	Anaplastic astrocytoma, IDH-mutant	G-CIMP-high	PN	1125	37.5	0	Mutant	non-codel	Methylated	No combined CNA	Mutant
TCGA.DB.A4XF	41	Female	1.93413903	3.893653439	diploid	Astrocytoma	III	Anaplastic astrocytoma, IDH-mutant	G-CIMP-high	PN	1008	33.6	0	Mutant	non-codel	Methylated	No combined CNA	WT
TCGA.DB.A4XG	34	Male	2.562987243	3.844566431	diploid	Oligodendroglioma	III	Anaplastic oligodendroglioma, IDH-mutant	Codel	PN	2190	73	0	Mutant	codel	Methylated	No combined CNA	WT
TCGA.DB.A4XH	53	Female	1.108290261	4.177176327	diploid	Oligoastrocytoma	II	Oligodendroglioma, IDH-mutant and 1p/19	Codel	NE	948	31.6	0	Mutant	codel	Methylated	No combined CNA	WT
TCGA.DB.A64L	67	Female	3.068516044	5.167173243	diploid	Oligodendroglioma	II	Oligodendroglioma, IDH-mutant and 1p/19	Codel	NE	726	24.2	0	Mutant	codel	Methylated	No combined CNA	WT
TCGA.DB.A64O	59	Male	3.144715338	6.437442489	Gain	Oligoastrocytoma	II	Diffuse astrocytoma, IDH-wildtype	Mesenchymal-like	NE	765	25.5	1	WT	non-codel	Unmethylated	Gain chr 7 & loss chr 10	WT
TCGA.DB.A64P	40	Male	2.101347522	1.750349241	diploid	Oligodendroglioma	III	Anaplastic oligodendroglioma, IDH-mutant	Codel	PN	903	30.1	0	Mutant	codel	Methylated	No combined CNA	WT
TCGA.DB.A64Q	31	Female	2.202417722	3.659365177	diploid	Oligoastrocytoma	II	Oligodendroglioma, IDH-mutant and 1p/19	Codel	PN	180	6	0	Mutant	codel	Methylated	No combined CNA	WT
TCGA.DB.A64R	24	Female	1.285698126	4.597602681	diploid	Oligodendroglioma	II	Oligodendroglioma, IDH-mutant and 1p/19	Codel	PN	942	31.4	0	Mutant	codel	Methylated	No combined CNA	WT
TCGA.DB.A64S	20	Male	2.300065137	3.553839684	diploid	Oligoastrocytoma	II	Diffuse astrocytoma, IDH-mutant	G-CIMP-high		822	27.4	0	Mutant	non-codel	Unmethylated	No combined CNA	Mutant
TCGA.DB.A64U	38	Female	2.644710013	3.523223197	diploid	Oligoastrocytoma	II	Oligodendroglioma, IDH-mutant and 1p/19	Codel	PN	834	27.8	0	Mutant	codel	Methylated	No combined CNA	WT
TCGA.DB.A64V	54	Male	4.62479107	3.720453592	diploid	Oligodendroglioma	II	Oligodendroglioma, IDH-mutant and 1p/19	Codel		876	29.2	0	Mutant	codel	Methylated	No combined CNA	WT
TCGA.DB.A64W	65	Female	-0.083141235	4.991807522	diploid	Oligoastrocytoma	III	Anaplastic oligodendroglioma, IDH-mutant	Codel	PN	432	14.4	1	Mutant	codel	Methylated	No combined CNA	WT
TCGA.DB.A64X	56	Female	3.099227866	4.177256073	Gain	Astrocytoma	III	Anaplastic astrocytoma, IDH-mutant	G-CIMP-high		555	18.5	0	Mutant	non-codel	Methylated	No combined CNA	WT
TCGA.DB.A75K	55	Female	4.414108465	4.251946271	diploid	Oligoastrocytoma	III	Anaplastic oligodendroglioma, IDH-mutant	Codel	PN	363	12.1	0	Mutant	codel	Methylated	No combined CNA	WT
TCGA.DB.A75L	36	Female	2.032347738	4.647095728	diploid	Astrocytoma	III	Anaplastic astrocytoma, IDH-mutant	G-CIMP-high	NE	336	11.2	0	Mutant	non-codel	Methylated	No combined CNA	WT
TCGA.DB.A75M	47	Male	2.171623176	3.497816717	diploid	Astrocytoma	II	Diffuse astrocytoma, IDH-mutant	G-CIMP-high		537	17.9	0	Mutant	non-codel	Methylated	No combined CNA	Mutant
TCGA.DB.A75O	29	Male	4.810751559	3.958285901	Gain	Astrocytoma	III	Anaplastic astrocytoma, IDH-mutant	G-CIMP-high		921	30.7	0	Mutant	non-codel	Methylated	No combined CNA	WT
TCGA.DB.A75P	25	Female	3.751570837	5.983233054	diploid	Astrocytoma	II	Diffuse astrocytoma, IDH-wildtype	PA-like	NE	486	16.2	0	WT	non-codel	Unmethylated	No combined CNA	WT
TCGA.DH.5140	38	Female	3.157868334	6.179379673	diploid	Oligoastrocytoma	III	Anaplastic astrocytoma, IDH-wildtype	PA-like	PN	600	20	1	WT	non-codel	Unmethylated	No combined CNA	WT
TCGA.DH.5141	32	Male	2.861975209	1.767061498	diploid	Oligodendroglioma	III	Anaplastic oligodendroglioma, IDH-mutant	Codel	PN	954	31.8	0	Mutant	codel	Methylated	No combined CNA	WT
TCGA.DH.5142	29	Male	4.246400486	2.382445921	diploid	Astrocytoma	III	Anaplastic astrocytoma, IDH-mutant	G-CIMP-high	PN	1917	63.9	0	Mutant	non-codel	Methylated	No combined CNA	Mutant
TCGA.DH.5143	30	Male	3.455346949	3.331676557	diploid	Oligoastrocytoma	III	Anaplastic astrocytoma, IDH-mutant	G-CIMP-high	NE	1383	46.1	0	Mutant	non-codel	Methylated	No combined CNA	WT
TCGA.DH.5144	56	Female	1.994724529	2.198682729	Gain	Oligodendroglioma	III	Anaplastic oligodendroglioma, IDH-mutant	Codel	PN	240	8	0	Mutant	codel	Methylated	No combined CNA	Mutant
TCGA.DH.A669	70	Male	3.684448481	3.531493752	Gain	Oligodendroglioma	III	Anaplastic oligodendroglioma, IDH-mutant	Codel	PN	906	30.2	1	Mutant	codel	Methylated	No combined CNA	WT
TCGA.DH.A66B	52	Male	3.07563531	1.86844972	NA	Astrocytoma	III	Anaplastic astrocytoma, IDH-mutant	G-CIMP-high		1260	42	0	Mutant	non-codel	Methylated	No combined CNA	WT
TCGA.DH.A66D	43	Female	3.885056932	4.115091522	Gain	Astrocytoma	III	Anaplastic astrocytoma, IDH-mutant	G-CIMP-high	PN	834	27.8	0	Mutant	non-codel	Methylated	No combined CNA	Mutant
TCGA.DH.A66F	49	Male	2.627396722	2.643879273	diploid	Oligodendroglioma	II	Oligodendroglioma, IDH-mutant and 1p/19	Codel	PN	516	17.2	0	Mutant	codel	Methylated	No combined CNA	WT
TCGA.DH.A66G	49	Female	2.812559834	4.778702709	Gain	Oligodendroglioma	III	Anaplastic astrocytoma, IDH-mutant	G-CIMP-high		516	17.2	0	Mutant	non-codel	Methylated	No combined CNA	Mutant
TCGA.DH.A7UR	59	Female	1.706906234	5.623612308	diploid	Oligodendroglioma	III	Anaplastic oligodendroglioma, IDH-mutant	Codel		1626	54.2	0	Mutant	codel	Methylated	No combined CNA	WT
TCGA.DH.A7US	50	Male	2.417542007	3.886130642	diploid	Oligodendroglioma	II	Oligodendroglioma, IDH-mutant and 1p/19	Codel	PN	696	23.2	0	Mutant	codel	Methylated	No combined CNA	WT
TCGA.DH.A7UT	30	Male	3.658976908	5.726700453	Gain	Astrocytoma	III	Anaplastic astrocytoma, IDH-mutant	G-CIMP-high		525	17.5	1	Mutant	non-codel	Methylated	No combined CNA	WT
TCGA.DH.A7UU	43	Male	2.620516033	3.44796263	diploid	Astrocytoma	III	Anaplastic astrocytoma, IDH-mutant	G-CIMP-high	PN	411	13.7	0	Mutant	non-codel	Methylated	No combined CNA	WT
TCGA.DH.A7UV	49	Male	2.952501312	3.870966334	Gain	Astrocytoma	III	Anaplastic astrocytoma, IDH-mutant	G-CIMP-high	PN	558	18.6	0	Mutant	non-codel	Methylated	No combined CNA	Mutant
TCGA.DU.5847	34	Female	4.2982624	6.069566843	diploid	Astrocytoma	III	Anaplastic astrocytoma, IDH-wildtype	Mesenchymal-like	ME	540	18	0	WT	non-codel	Methylated	No combined CNA	WT
TCGA.DU.5849	48	Male	1.394898224	4.928332419	Gain	Oligodendroglioma	II	Oligodendroglioma, IDH-mutant and 1p/19	Codel	NE	438	14.6	0	Mutant	codel	Methylated	No combined CNA	WT
TCGA.DU.5851	40	Female	2.92314918	5.125411404	diploid	Oligoastrocytoma	III	Anaplastic astrocytoma, IDH-mutant	G-CIMP-high		525	17.5	0	Mutant	non-codel	Unmethylated	No combined CNA	Mutant
TCGA.DU.5852	61	Female	5.095755674	5.246225649	diploid	Oligoastrocytoma	III	Anaplastic astrocytoma, IDH-wildtype	Mesenchymal-like	CL	201	6.7	1	WT	non-codel	Methylated	No combined CNA	Mutant
TCGA.DU.5853	29	Male	3.102154192	3.340747401	diploid	Oligoastrocytoma	II	Diffuse astrocytoma, IDH-mutant	G-CIMP-high	PN	402	13.4	0	Mutant	non-codel	Methylated	No combined CNA	Mutant
TCGA.DU.5854	57	Female	4.393601537	4.351790849	Gain	Astrocytoma	III	Anaplastic astrocytoma, IDH-wildtype	Mesenchymal-like	CL	252	8.4	0	WT	non-codel	Unmethylated	Gain chr 7 & loss chr 10	WT
TCGA.DU.5855	49	Female	4.008854364	3.788309851	Gain	Oligoastrocytoma	III	Anaplastic astrocytoma, IDH-mutant	G-CIMP-high	PN	204	6.8	0	Mutant	non-codel	Methylated	No combined CNA	Mutant
TCGA.DU.5870	34	Female	1.914908892	2.285638949	diploid	Oligodendroglioma	II	Oligodendroglioma, IDH-mutant and 1p/19	Codel	PN	5469	182.3	0	Mutant	codel	Methylated	No combined CNA	WT
TCGA.DU.5871	37	Female	1.69692859	3.772117912	diploid	Oligoastrocytoma	II	Diffuse astrocytoma, IDH-mutant	G-CIMP-high	PN	567	18.9	0	Mutant	non-codel	Methylated	No combined CNA	Mutant
TCGA.DU.5872	43	Female	3.023894043	4.134878054	diploid	Oligoastrocytoma	II	Diffuse astrocytoma, IDH-mutant	G-CIMP-high	PN	525	17.5	0	Mutant	non-codel	Methylated	No combined CNA	Mutant
TCGA.DU.5874	62	Female	0.96761131	2.853556586	diploid	Oligodendroglioma	II	Oligodendroglioma, IDH-mutant and 1p/19	Codel	PN	456	15.2	0	Mutant	codel	Methylated	No combined CNA	WT
TCGA.DU.6392	35	Female	2.983768886	6.226701185	Gain	Astrocytoma	III	Anaplastic astrocytoma, IDH-wildtype	PA-like	PN	6336	211.2	0	WT	non-codel	Unmethylated	No combined CNA	Mutant
TCGA.DU.6393	66	Male	1.740711733	2.89373109	diploid	Oligodendroglioma	III	Anaplastic oligodendroglioma, IDH-mutant	Codel	PN	1563	52.1	1	Mutant	codel	Methylated	No combined CNA	WT
TCGA.DU.6394	53	Male	1.386424705	2.427284346	diploid	Oligodendroglioma	III	Anaplastic oligodendroglioma, IDH-mutant	Codel	PN	672	22.4	1	Mutant	codel	Methylated	No combined CNA	WT
TCGA.DU.6395	31	Male	1.720803782	3.743514968	Gain	Oligoastrocytoma	II	Diffuse astrocytoma, IDH-mutant	G-CIMP-high	PN	1470	49	1	Mutant	non-codel	Methylated	No combined CNA	Mutant
TCGA.DU.6396	31	Female	4.784666251	1.40506656	diploid	Oligoastrocytoma	III	Anaplastic astrocytoma, IDH-mutant	G-CIMP-high	PN	2256	75.2	1	Mutant	non-codel	Methylated	No combined CNA	Mutant
TCGA.DU.6397	45	Male	2.43151599	1.750692249	diploid	Oligodendroglioma	III	Anaplastic oligodendroglioma, IDH-mutant	Codel	PN	1383	46.1	1	Mutant	codel	Methylated	No combined CNA	WT
TCGA.DU.6399	54	Male	2.46524314	2.546857428	Gain	Oligodendroglioma	II	Oligodendroglioma, NOS	G-CIMP-high	PN	1974	65.8	1	Mutant	non-codel	Methylated	No combined CNA	Mutant
TCGA.DU.6400	66	Female	2.203921541	3.437134504	Gain	Oligodendroglioma	II	Oligodendroglioma, IDH-mutant and 1p/19	Codel	PN	36	1.2	1	Mutant	codel	Methylated	No combined CNA	WT
TCGA.DU.6401	31	Female	1.068258062	3.058801354	diploid	Oligodendroglioma	II	Oligodendroglioma, NOS	G-CIMP-high	PN	2625	87.5	1	Mutant	non-codel	Methylated	No combined CNA	Mutant
TCGA.DU.6402	52	Male	4.155619721	2.957728912	Gain	Astrocytoma	III	Anaplastic astrocytoma, IDH-wildtype	Classic-like	CL	210	7	1	WT	non-codel	Unmethylated	Gain chr 7 & loss chr 10	WT
TCGA.DU.6403	60	Female	4.834574705	5.458418097	Gain	Oligoastrocytoma	III	Anaplastic astrocytoma, IDH-wildtype	Classic-like	PN	348	11.6	1	WT	non-codel	Unmethylated	Gain chr 7 & loss chr 10	WT
TCGA.DU.6404	24	Female	4.867387478	5.654266537	Gain	Oligodendroglioma	III	Anaplastic oligodendroglioma, NOS	PA-like	CL	4011	133.7	1	WT	non-codel	Unmethylated	No combined CNA	WT
TCGA.DU.6405	51	Female	3.430887386	3.079531717	Gain	Astrocytoma	III	Anaplastic astrocytoma, IDH-wildtype	Classic-like	CL	597	19.9	1	WT	non-codel	Methylated	Gain chr 7 & loss chr 10	WT
TCGA.DU.6406	59	Female	2.369354805	4.763985139	Gain	Oligoastrocytoma	III	Anaplastic astrocytoma, IDH-wildtype	Classic-like	CL	504	16.8	1	WT	non-codel	Unmethylated	Gain chr 7 & loss chr 10	WT
TCGA.DU.6407	35	Female	2.146068641	1.129810535	Gain	Oligodendroglioma	II	Oligodendroglioma, NOS	G-CIMP-high	PN	2835	94.5	1	Mutant	non-codel	Methylated	No combined CNA	Mutant
TCGA.DU.6408	23	Female	2.27643711	4.315957426	diploid	Oligodendroglioma	III	Anaplastic oligodendroglioma, NOS	G-CIMP-high	PN	3423	114.1	1	Mutant	non-codel	Methylated	No combined CNA	Mutant
TCGA.DU.6410	56	Male	0.577344228	3.425674135	Gain	Oligodendroglioma	III	Anaplastic oligodendroglioma, IDH-mutant	Codel	PN	240	8	0	Mutant	codel	Methylated	No combined CNA	WT
TCGA.DU.6542	25	Male	3.162145571	3.615380983	diploid	Oligoastrocytoma	III	Anaplastic astrocytoma, IDH-mutant	G-CIMP-high	PN	72	2.4	0	Mutant	non-codel	Methylated	No combined CNA	WT
TCGA.DU.7006	60	Female	3.806344682	4.625720531	Gain	Astrocytoma	III	Anaplastic astrocytoma, IDH-wildtype	Mesenchymal-like	ME	345	11.5	1	WT	non-codel	Methylated	Gain chr 7 & loss chr 10	WT
TCGA.DU.7007	33	Male	2.83594428	4.261929419	diploid	Astrocytoma	II	Diffuse astrocytoma, IDH-mutant	G-CIMP-high	ME	1890	63	1	Mutant	non-codel	Methylated	No combined CNA	Mutant
TCGA.DU.7008	41	Female	2.228449313	4.156970927	Gain	Oligodendroglioma	II	Oligodendroglioma, NOS	G-CIMP-high	PN	4686	156.2	0	Mutant	non-codel	Methylated	No combined CNA	Mutant
TCGA.DU.7009	32	Female	1.80933212	4.220794267	diploid	Oligodendroglioma	II	Oligodendroglioma, IDH-mutant and 1p/19	Codel	PN	4632	154.4	1	Mutant	codel	Methylated	No combined CNA	WT
TCGA.DU.7010	58	Female	2.491493981	3.434748308	diploid	Astrocytoma	III	Anaplastic astrocytoma, IDH-mutant	G-CIMP-low	PN	450	15	1	Mutant	non-codel	Methylated	No combined CNA	WT
TCGA.DU.7011	25	Male	3.733007184	4.517628449	Gain	Oligoastrocytoma	II	Diffuse astrocytoma, IDH-mutant	G-CIMP-high	NE	3156	105.2	1	Mutant	non-codel	Unmethylated	No combined CNA	Mutant
TCGA.DU.7012	74	Female	4.704479153	5.788403825	Gain	Astrocytoma	III	Anaplastic astrocytoma, IDH-wildtype	Mesenchymal-like	ME	195	6.5	1	WT	non-codel	Methylated	Gain chr 7 & loss chr 10	WT
TCGA.DU.7013	59	Male	3.242495805	4.071462363	Gain	Astrocytoma	III	Anaplastic astrocytoma, IDH-wildtype	Classic-like	CL	264	8.8	1	WT	non-codel	Unmethylated	Gain chr 7 & loss chr 10	WT
TCGA.DU.7014	59	Male	0.506195692	2.614238683	Gain	Oligodendroglioma	II	Oligodendroglioma, NOS		PN	3522	117.4	1		non-codel	Methylated	No combined CNA	
TCGA.DU.7015	41	Female	3.990800602	2.317101428	Gain	Oligodendroglioma	II	Oligodendroglioma, NOS	G-CIMP-high	PN	2724	90.8	0	Mutant	non-codel	Methylated	No combined CNA	Mutant
TCGA.DU.7018	57	Female	0.806365306	1.993312058	diploid	Oligodendroglioma	III	Anaplastic oligodendroglioma, IDH-mutant	Codel	PN	921	30.7	1	Mutant	codel	Methylated	No combined CNA	WT

TCGA.DU.7019	39	Male	1.500547137	3.26477661	diploid	Oligoastrocytoma	III	Anaplastic astrocytoma, IDH-mutant	G-CIMP-high		789	26.3	0	Mutant	non-codel	Methylated	No combined CNA	WT
TCGA.DU.7290	45	Female	3.346616558	8.945797882	diploid	Astrocytoma	III	Anaplastic astrocytoma, IDH-wildtype	Mesenchymal-like	ME	312	10.4	1	WT	non-codel	Unmethylated	No combined CNA	WT
TCGA.DU.7292	69	Male	2.488052203	5.429180747	Gain	Astrocytoma	III	Anaplastic astrocytoma, IDH-wildtype	Classic-like	NE	240	8	1	WT	non-codel	Methylated	No combined CNA	WT
TCGA.DU.7294	53	Female	0.590434352	2.724933981	diploid	Oligodendroglioma	II	Oligodendroglioma, IDH-mutant and 1p/19	Codel	PN	2829	94.3	0	Mutant	codel	Methylated	No combined CNA	WT
TCGA.DU.7298	38	Female	2.523110259	3.536413529	diploid	Astrocytoma	III	Anaplastic astrocytoma, IDH-mutant	G-CIMP-high	PN	567	18.9	1	Mutant	non-codel	Methylated	No combined CNA	Mutant
TCGA.DU.7299	33	Male	1.892546459	2.551589842	diploid	Astrocytoma	III	Anaplastic astrocytoma, IDH-mutant	G-CIMP-high	PN	1320	44	1	Mutant	non-codel	Methylated	No combined CNA	WT
TCGA.DU.7300	53	Female	2.69081934	4.367182329	diploid	Oligodendroglioma	III	Anaplastic oligodendroglioma, IDH-mutant	Codel	NE	1860	62	1	Mutant	codel	Methylated	No combined CNA	WT
TCGA.DU.7301	53	Male	2.895225055	2.157852169	Gain	Oligodendroglioma	II	Oligodendroglioma, NOS	G-CIMP-high		777	25.9	1	Mutant	non-codel	Methylated	No combined CNA	Mutant
TCGA.DU.7302	48	Female	1.690059451	4.141089407	diploid	Oligodendroglioma	III	Anaplastic oligodendroglioma, IDH-mutant	Codel	NE	1809	60.3	0	Mutant	codel	Methylated	No combined CNA	WT
TCGA.DU.7304	43	Male	3.642066138	4.496954413	diploid	Oligoastrocytoma	III	Anaplastic astrocytoma, IDH-mutant	G-CIMP-high	NE	699	23.3	1	Mutant	non-codel	Methylated	No combined CNA	Mutant
TCGA.DU.7306	67	Male	1.876094513	0.574634395	Gain	Oligoastrocytoma	II	Diffuse astrocytoma, IDH-mutant	G-CIMP-high		1260	42	0	Mutant	non-codel	Methylated	No combined CNA	Mutant
TCGA.DU.7309	41	Female	2.23526753	5.12164955	Gain	Oligodendroglioma	III	Anaplastic oligodendroglioma, NOS	G-CIMP-high	NE	84	2.8	0	Mutant	non-codel	Methylated	No combined CNA	Mutant
TCGA.DU.8158	57	Female	3.766171089	8.871678577	diploid	Astrocytoma	III	Anaplastic astrocytoma, IDH-wildtype	Mesenchymal-like		153	5.1	1	WT	non-codel	Unmethylated	No combined CNA	WT
TCGA.DU.8161	63	Female	4.245077275	5.549505919	Gain	Oligoastrocytoma	III	Anaplastic astrocytoma, IDH-wildtype	Mesenchymal-like	CL	711	23.7	1	WT	non-codel	Unmethylated	Gain chr 7 & loss chr 10	WT
TCGA.DU.8162	61	Female	3.161355641	5.372457653	diploid	Oligoastrocytoma	III	Anaplastic astrocytoma, IDH-wildtype	Mesenchymal-like	NE	438	14.6	1	WT	non-codel	Unmethylated	No combined CNA	WT
TCGA.DU.8163	29	Male	3.414338525	4.646277884	Gain	Oligoastrocytoma	III	Anaplastic astrocytoma, IDH-mutant	G-CIMP-high	PN	621	20.7	0	Mutant	non-codel	Unmethylated	No combined CNA	Mutant
TCGA.DU.8164	51	Male	1.44948214	3.640019294	diploid	Oligodendroglioma	II	Oligodendroglioma, IDH-mutant and 1p/19	Codel	PN	642	21.4	0	Mutant	codel	Methylated	No combined CNA	WT
TCGA.DU.8165	60	Female	4.023769875	7.308878688	Gain	Oligodendroglioma	III	Anaplastic oligodendroglioma, NOS	Mesenchymal-like		573	19.1	0	WT	non-codel	Unmethylated	Gain chr 7 & loss chr 10	WT
TCGA.DU.8166	29	Female	1.444136957	4.176386607	Gain	Oligoastrocytoma	II	Diffuse astrocytoma, IDH-mutant	G-CIMP-high	PN	510	17	0	Mutant	non-codel	Methylated	No combined CNA	Mutant
TCGA.DU.8167	69	Female	3.210170636	3.998475348	diploid	Oligoastrocytoma	II	Diffuse astrocytoma, IDH-mutant	G-CIMP-high	PN	465	15.5	0	Mutant	non-codel	Methylated	No combined CNA	WT
TCGA.DU.8168	55	Female	2.165558254	3.09000653	diploid	Oligodendroglioma	III	Anaplastic oligodendroglioma, IDH-mutant	Codel	PN	426	14.2	0	Mutant	codel	Methylated	No combined CNA	WT
TCGA.DU.A5TP	33	Male	3.63282657	3.347056138	diploid	Astrocytoma	III	Anaplastic astrocytoma, IDH-mutant	G-CIMP-high	ME	999	33.3	0	Mutant	non-codel	Methylated	No combined CNA	Mutant
TCGA.DU.A5TR	51	Male	5.255274114	4.778213834	Gain	Oligoastrocytoma	II	Diffuse astrocytoma, IDH-mutant	G-CIMP-high	ME	897	29.9	0	Mutant	non-codel	Methylated	No combined CNA	Mutant
TCGA.DU.A5TS	42	Male	3.757151291	3.057467602	diploid	Oligodendroglioma	II	Oligodendroglioma, NOS	G-CIMP-high		951	31.7	0	Mutant	non-codel	Methylated	No combined CNA	Mutant
TCGA.DU.A5TT	70	Male	3.191973167	8.073360751	Gain	Oligodendroglioma	III	Anaplastic oligodendroglioma, NOS	Mesenchymal-like		732	24.4	0	WT	non-codel	Methylated	No combined CNA	WT
TCGA.DU.A5TU	62	Female	5.2217843	4.126807703	Gain	Astrocytoma	II	Diffuse astrocytoma, IDH-mutant	G-CIMP-high	PN	786	26.2	0	Mutant	non-codel	Methylated	No combined CNA	Mutant
TCGA.DU.A5TW	33	Female	1.84631278	4.988093856	Gain	Astrocytoma	III	Anaplastic astrocytoma, IDH-mutant	G-CIMP-high	PN	750	25	0	Mutant	non-codel	Methylated	No combined CNA	Mutant
TCGA.DU.A5TY	46	Female	5.396313644	4.900746075	Gain	Astrocytoma	III	Anaplastic astrocytoma, IDH-wildtype	Mesenchymal-like	CL	1020	34	1	WT	non-codel	Methylated	Gain chr 7 & loss chr 10	WT
TCGA.DU.A6S2	37	Female	1.189603197	5.387889806	diploid	Oligodendroglioma	II	Oligodendroglioma, IDH-mutant and 1p/19	Codel	NE	768	25.6	0	Mutant	codel	Methylated	No combined CNA	WT
TCGA.DU.A6S3	60	Male	1.586164246	4.91334901	diploid	Oligodendroglioma	II	Oligodendroglioma, IDH-mutant and 1p/19	Codel	PN	648	21.6	0	Mutant	codel	Methylated	No combined CNA	WT
TCGA.DU.A6S6	35	Female	2.749126609	5.620806317	Gain	Oligoastrocytoma	II	Oligodendroglioma, IDH-mutant and 1p/19	Codel	NE	2853	95.1	0	Mutant	codel	Methylated	No combined CNA	WT
TCGA.DU.A6S7	27	Female	3.90446483	3.368181655	diploid	Astrocytoma	III	Anaplastic astrocytoma, IDH-mutant	G-CIMP-high	PN	630	21	0	Mutant	non-codel	Methylated	No combined CNA	Mutant
TCGA.DU.A6S8	74	Female	1.646899949	3.249930709	diploid	Oligodendroglioma	III	Anaplastic oligodendroglioma, IDH-mutant	Codel	PN	669	22.3	0	Mutant	codel	Methylated	No combined CNA	WT
TCGA.DU.A76K	87	Male	1.900490089	4.973790339	Gain	Oligodendroglioma	II	Oligodendroglioma, NOS	Classic-like	NE	342	11.4	1	WT	non-codel	Unmethylated	Gain chr 7 & loss chr 10	WT
TCGA.DU.A76L	54	Male	3.655683839	6.998635557	Gain	Oligodendroglioma	III	Anaplastic oligodendroglioma, NOS	Mesenchymal-like	ME	804	26.8	1	WT	non-codel	Methylated	Gain chr 7 & loss chr 10	WT
TCGA.DU.A76O	30	Male	4.309634598	3.668970055	Gain	Astrocytoma	II	Diffuse astrocytoma, IDH-mutant	G-CIMP-high	PN	516	17.2	0	Mutant	non-codel	Methylated	No combined CNA	Mutant
TCGA.DU.A76R	51	Male	2.146915848	4.015399441	Gain	Oligodendroglioma	III	Anaplastic oligodendroglioma, IDH-mutant	Codel	NE	639	21.3	1	Mutant	codel	Methylated	No combined CNA	WT
TCGA.DU.A7T6	73	Female	1.356087454	5.398295639	diploid	Oligodendroglioma	III	Anaplastic oligodendroglioma, IDH-mutant	Codel	PN	540	18	1	Mutant	codel	Methylated	No combined CNA	WT
TCGA.DU.A7T8	35	Male	3.010761939	4.245191393	Gain	Oligoastrocytoma	III	Anaplastic astrocytoma, IDH-mutant	G-CIMP-high	PN	4170	139	1	Mutant	non-codel	Methylated	No combined CNA	Mutant
TCGA.DU.A7TA	32	Male	3.682348561	4.720606811	Gain	Oligodendroglioma	II	Oligodendroglioma, NOS	G-CIMP-high	PN	2529	84.3	0	Mutant	non-codel	Methylated	No combined CNA	Mutant
TCGA.DU.A7TB	56	Male	3.197503729	6.116441697	diploid	Oligodendroglioma	II	Oligodendroglioma, NOS	PA-like	PN	1545	51.5	0	WT	non-codel	Unmethylated	No combined CNA	WT
TCGA.DU.A7TC	32	Male	3.41362116	5.553750617	diploid	Astrocytoma	II	Diffuse astrocytoma, IDH-mutant	G-CIMP-high	NE	1122	37.4	0	Mutant	non-codel	Methylated	No combined CNA	WT
TCGA.DU.A7TD	52	Male	5.377547192	3.84796685	Gain	Oligoastrocytoma	III	Anaplastic astrocytoma, IDH-wildtype	Mesenchymal-like	CL	225	7.5	1	WT	non-codel	Unmethylated	Gain chr 7 & loss chr 10	WT
TCGA.DU.A7TG	40	Male	2.434534825	6.174188671	diploid	Oligodendroglioma	II	Oligodendroglioma, NOS	G-CIMP-high	NE	1332	44.4	1	Mutant	non-codel	Methylated	No combined CNA	WT
TCGA.DU.A7TI	32	Male	3.339222918	4.54799265	Gain	Astrocytoma	III	Anaplastic astrocytoma, NOS		NE	1167	38.9	1		non-codel	Methylated	No combined CNA	
TCGA.DU.A7TJ	55	Male	4.065141438	2.70015115	Gain	Astrocytoma	III	Anaplastic astrocytoma, IDH-wildtype	Mesenchymal-like	CL	18	0.6	0	WT	non-codel	Methylated	Gain chr 7 & loss chr 10	WT
TCGA.E1.5302	41	Male	4.208111425	2.508834274	Gain	Astrocytoma	III	Anaplastic astrocytoma, IDH-mutant	G-CIMP-high	PN	1503	50.1	1	Mutant	non-codel	Methylated	No combined CNA	Mutant
TCGA.E1.5303	38	Male	4.505789328	3.045635177	diploid	Astrocytoma	III	Anaplastic astrocytoma, IDH-mutant	G-CIMP-high		2025	67.5	1	Mutant	non-codel	Methylated	No combined CNA	Mutant
TCGA.E1.5304	42	Male	1.384823459	2.889707158	Gain	Astrocytoma	III	Anaplastic astrocytoma, IDH-mutant	G-CIMP-low	PN	1233	41.1	1	Mutant	non-codel	Unmethylated	No combined CNA	Mutant
TCGA.E1.5305	34	Male	1.82321946	3.027366405	diploid	Astrocytoma	III	Anaplastic astrocytoma, IDH-mutant	G-CIMP-high	NE	2400	80	1	Mutant	non-codel	Methylated	No combined CNA	Mutant
TCGA.E1.5307	62	Female	2.401930769	3.40603319	diploid	Astrocytoma	III	Anaplastic astrocytoma, IDH-mutant	G-CIMP-high	PN	1737	57.9	1	Mutant	non-codel	Methylated	No combined CNA	Mutant
TCGA.E1.5311	31	Male	2.072140109	4.137946101	diploid	Oligodendroglioma	III	Anaplastic oligodendroglioma, IDH-mutant	Codel	PN	4029	134.3	1	Mutant	codel	Methylated	No combined CNA	WT
TCGA.E1.5318	42	Female	1.81077212	3.397296424	diploid	Oligodendroglioma	II	Oligodendroglioma, IDH-mutant and 1p/19	Codel	PN	2346	78.2	1	Mutant	codel	Methylated	No combined CNA	WT
TCGA.E1.5319	48	Female	1.892546459	1.946131952	diploid	Oligodendroglioma	II	Oligodendroglioma, IDH-mutant and 1p/19	Codel	PN	2868	95.6	1	Mutant	codel	Methylated	No combined CNA	WT
TCGA.E1.5322	38	Female	2.18551772	2.359830367	Gain	Oligoastrocytoma	II	Diffuse astrocytoma, IDH-mutant	G-CIMP-high	PN	3924	130.8	1	Mutant	non-codel	Methylated	No combined CNA	Mutant
TCGA.E1.A7YD	57	Male	3.606454073	6.531156851	Gain	Astrocytoma	III	Anaplastic astrocytoma, IDH-wildtype	Mesenchymal-like	CL	429	14.3	1	WT	non-codel	Unmethylated	Gain chr 7 & loss chr 10	WT
TCGA.E1.A7YE	32	Female	3.098436402	3.351472858	diploid	Astrocytoma	III	Anaplastic astrocytoma, IDH-mutant	G-CIMP-low	ME	873	29.1	1	Mutant	non-codel	Unmethylated	No combined CNA	Mutant
TCGA.E1.A7YH	47	Female	3.670035845	4.253437186	diploid	Astrocytoma	III	Anaplastic astrocytoma, IDH-mutant	G-CIMP-high		2796	93.2	1	Mutant	non-codel	Methylated	No combined CNA	Mutant
TCGA.E1.A7YI	33	Female	3.019399468	2.89917563	diploid	Astrocytoma	III	Anaplastic astrocytoma, IDH-mutant	G-CIMP-low	PN	108	3.6	1	Mutant	non-codel	Methylated	No combined CNA	Mutant
TCGA.E1.A7YJ	55	Male	5.361501689	3.608974788	diploid	Astrocytoma	III	Anaplastic astrocytoma, IDH-wildtype	Classic-like	CL	585	19.5	1	WT	non-codel	Unmethylated	Gain chr 7 & loss chr 10	WT
TCGA.E1.A7YK	52	Male	5.071192033	3.853067474	diploid	Astrocytoma	III	Anaplastic astrocytoma, IDH-mutant	G-CIMP-high	ME	372	12.4	1	Mutant	non-codel	Methylated	No combined CNA	Mutant
TCGA.E1.A7YL	46	Male	5.377092507	6.762326224	Gain	Astrocytoma	III	Anaplastic astrocytoma, IDH-wildtype	Mesenchymal-like	ME	486	16.2	1	WT	non-codel	Unmethylated	Gain chr 7 & loss chr 10	WT
TCGA.E1.A7YM	63	Male	3.73433027	4.847155089	Gain	Astrocytoma	III	Anaplastic astrocytoma, IDH-wildtype	Mesenchymal-like	NE	639	21.3	1	WT	non-codel	Unmethylated	Gain chr 7 & loss chr 10	WT
TCGA.E1.A7YN	63	Female	6.448915814	4.551989661	Gain	Astrocytoma	III	Anaplastic astrocytoma, IDH-wildtype	Mesenchymal-like	ME	717	23.9	1	WT	non-codel	Methylated	Gain chr 7 & loss chr 10	WT
TCGA.E1.A7YO	45	Male	1.381560255	4.264679042	diploid	Oligodendroglioma	III	Anaplastic oligodendroglioma, IDH-mutant	Codel	PN	2250	75	1	Mutant	codel	Methylated	No combined CNA	WT
TCGA.E1.A7YQ	58	Female	4.683387673	5.298478701	Gain	Oligodendroglioma	III	Anaplastic oligodendroglioma, NOS	Classic-like	ME	1557	51.9	1	WT	non-codel	Unmethylated	Gain chr 7 & loss chr 10	WT
TCGA.E1.A7YS	71	Male	0.970780046	3.139780528	diploid	Oligodendroglioma	III	Anaplastic oligodendroglioma, IDH-mutant	Codel	PN	459	15.3	1	Mutant	codel	Methylated	No combined CNA	WT
TCGA.E1.A7YU	42	Male	2.3934162	4.366560021	diploid	Oligoastrocytoma	III	Anaplastic astrocytoma, IDH-mutant	G-CIMP-high	NE	24	0.8	1	Mutant	non-codel	Methylated	No combined CNA	WT
TCGA.E1.A7YV	26	Female	1.467696836	4.588264894	diploid	Oligoastrocytoma	III	Anaplastic astrocytoma, IDH-mutant	G-CIMP-low	PN	975	32.5	1	Mutant	non-codel	Methylated	No combined CNA	Mutant
TCGA.E1.A7YW	28	Male	2.212444829	4.022207998	diploid	Oligoastrocytoma	II	Diffuse astrocytoma, IDH-mutant	G-CIMP-high	PN	1104	36.8	1	Mutant	non-codel	Methylated	No combined CNA	Mutant
TCGA.E1.A7YY	27	Female	3.380743299	6.353432689	diploid	Oligodendroglioma	II	Oligodendroglioma, NOS	G-CIMP-high	NE	4383	146.1	1	Mutant	non-codel	Methylated	No combined CNA	WT
TCGA.E1.A7Z2	58	Female	3.121728281	4.727664558	NA	Oligodendroglioma	II	Oligodendroglioma, NOS	Mesenchymal-like	NE	393	13.1	1	WT	non-codel	Unmethylated	Gain chr 7 & loss chr 10	WT
TCGA.E1.A7Z3	31	Female	3.313724735	5.707483378	Gain	Astrocytoma	II	Diffuse astrocytoma, IDH-mutant	G-CIMP-high	NE	2205	73.5	1	Mutant	non-codel	Methylated	No combined CNA	Mutant
TCGA.E1.A7Z4	35	Male	3.881459065	2.762476388	diploid	Astrocytoma	II	Diffuse astrocytoma, IDH-mutant	G-CIMP-high		4353	145.1	1	Mutant	non-codel	Methylated	No combined CNA	Mutant
TCGA.E1.A7Z6	41	Female	3.164561041	2.882995122	Gain	Astrocytoma	II	Diffuse astrocytoma, IDH-mutant	G-CIMP-high		972	32.4	1	Mutant	non-codel	Methylated	No combined CNA	Mutant
TCGA.EZ.7264	47	Female	0.421155961	1.988339307	diploid	Oligodendroglioma	II	Oligodendroglioma, IDH-mutant and 1p/19	Codel	PN	1185	39.5	0	Mutant	codel	Methylated	No combined CNA	WT
TCGA.F6.A8O3	34	Male	0.659833246	3.162371186	diploid	Oligodendroglioma	II	Oligodendroglioma, IDH-mutant and 1p/19	Codel		6	0.2	0	Mutant	codel	Methylated	No combined CNA	WT
TCGA.F6.A8O4	44	Male	2.522959662	4.203506581	Gain	Astrocytoma	II	Diffuse astrocytoma, IDH-mutant	G-CIMP-high		6	0.2	0	Mutant	non-codel	Methylated	No combined CNA	Mutant
TCGA.FG.5962	54	Male	1.747215512	4.778692198	Gain	Oligodendroglioma	III	Anaplastic oligodendroglioma, IDH-mutant	Codel	NE	1434	47.8	0	Mutant	codel	Methylated	No combined CNA	WT
TCGA.FG.5963	23	Male	5.589982151	5.45740058	diploid	Astrocytoma	III	Anaplastic astrocytoma, IDH-wildtype	PA-like	ME	765	25.5	1	WT	non-codel	Unmethylated	No combined CNA	Mutant
TCGA.FG.5964	62	Male	4.472715205	5.263774349	diploid	Oligodendroglioma	II	Oligodendroglioma, IDH-mutant and 1p/19	Codel	PN	1566	52.2	0	Mutant	codel	Methylated	No combined CNA	WT
TCGA.FG.5965	39	Female	2.861915675	3.382238392	Gain	Oligoastrocytoma	II	Diffuse astrocytoma, IDH-mutant	G-CIMP-high	PN	1104	36.8	1	Mutant	non-codel	Methylated	No combined CNA	Mutant
TCGA.FG.6688	59	Female	4.274686579	7.212798876	Gain	Astrocytoma	III	Anaplastic astrocytoma, IDH-wildtype	Mesenchymal-like		564	18.8	0	WT	non-codel	Methylated	Gain chr 7 & loss chr 10	WT
TCGA.FG.6689	30	Male	2.881116409	4.568889439	diploid	Astrocytoma	II	Diffuse astrocytoma, IDH-mutant	G-CIMP-high	NE	447	14.9	0	Mutant	non-codel	Methylated	No combined CNA	Mutant
TCGA.FG.6690	70	Male	2.20798653	2.981870915	Gain	Oligodendroglioma	II	Oligodendroglioma, NOS	G-CIMP-high	PN	1275	42.5	0	Mutant	non-codel	Methylated	No combined CNA	Mutant
TCGA.FG.6691	23	Female	2.907198338	3.220639513	Gain	Astrocytoma	II	Diffuse astrocytoma, IDH-mutant	G-CIMP-high	PN	1239	41.3	0	Mutant	non-codel	Unmethylated	No combined CNA	Mutant
TCGA.FG.6692	63	Male	4.909552316	5.743331846	Gain	Oligodendroglioma	III	Anaplastic oligodendroglioma, NOS	Classic-like	CL	552	18.4	1	WT	non-codel	Methylated	Gain chr 7 & loss chr 10	WT
TCGA.FG.7634	28	Male	2.707789805	3.682674417	Gain	Oligodendroglioma	II	Oligodendroglioma, IDH-mutant and 1p/19	Codel	PN	459	15.3	0	Mutant	codel	Methylated	No combined CNA	WT

TCGA.FG.7636	48	Male	2.785948049	4.465700261	diploid	Astrocytoma	III	Anaplastic astrocytoma, IDH-mutant	G-CIMP-high	PN	537	17.9	0	Mutant	non-codel	Methylated	No combined CNA	Mutant
TCGA.FG.7637	49	Male	0.645148266	1.63862995	NA	Oligoastrocytoma	II	Diffuse astrocytoma, IDH-mutant	Codel	PN	1203	40.1	0	Mutant	non-codel	Methylated		WT
TCGA.FG.7638	31	Female	2.617651119	4.060756448	diploid	Oligodendroglioma	III	Anaplastic oligodendroglioma, IDH-mutant	Codel	NE	678	22.6	0	Mutant	codel	Methylated	No combined CNA	Mutant
TCGA.FG.7641	31	Male	1.597698019	4.924099886	diploid	Oligodendroglioma	II	Oligodendroglioma, IDH-mutant and 1p/19	Codel	NE	618	20.6	0	Mutant	codel	Methylated	No combined CNA	WT
TCGA.FG.7643	49	Female	1.765831741	5.766436149	Gain	Oligoastrocytoma	II	Diffuse astrocytoma, IDH-wildtype	Mesenchymal-like	NE	603	20.1	0	WT	non-codel	Methylated	No combined CNA	WT
TCGA.FG.8181	23	Male	2.762221232	6.762501635	diploid	Oligoastrocytoma	III	Anaplastic astrocytoma, IDH-wildtype	PA-like	NE	849	28.3	0	WT	non-codel	Unmethylated	No combined CNA	WT
TCGA.FG.8182	35	Male	2.736128588	3.539444219	Gain	Oligodendroglioma	II	Oligodendroglioma, NOS	G-CIMP-high	PN	411	13.7	0	Mutant	non-codel	Methylated	No combined CNA	Mutant
TCGA.FG.8185	37	Male	2.947423038	2.65558081	diploid	Astrocytoma	III	Anaplastic astrocytoma, IDH-mutant	G-CIMP-high	PN	426	14.2	0	Mutant	non-codel	Methylated	No combined CNA	Mutant
TCGA.FG.8186	42	Female	4.061041705	4.053884622	Gain	Oligoastrocytoma	III	Anaplastic oligodendroglioma, IDH-mutant	Codel		480	16	0	Mutant	codel	Methylated	No combined CNA	WT
TCGA.FG.8187	31	Male	2.095451881	4.326530158	diploid	Oligoastrocytoma	II	Oligodendroglioma, IDH-mutant and 1p/19	Codel	NE	603	20.1	0	Mutant	codel	Methylated	No combined CNA	WT
TCGA.FG.8188	41	Male	2.793375711	1.846994687	diploid	Oligoastrocytoma	II	Diffuse astrocytoma, IDH-mutant	G-CIMP-high		450	15	0	Mutant	non-codel	Methylated	No combined CNA	Mutant
TCGA.FG.8189	33	Female	3.332836804	6.926744302	diploid	Oligodendroglioma	II	Oligodendroglioma, NOS	G-CIMP-high	NE	675	22.5	0	Mutant	non-codel	Methylated	No combined CNA	WT
TCGA.FG.8191	30	Male	1.915138426	3.495886998	diploid	Oligodendroglioma	III	Anaplastic oligodendroglioma, NOS	G-CIMP-high	PN	978	32.6	0	Mutant	non-codel	Unmethylated	No combined CNA	Mutant
TCGA.FG.A4MT	27	Female	1.916667717	4.354318154	diploid	Oligodendroglioma	II	Oligodendroglioma, NOS	G-CIMP-high	PN	1149	38.3	0	Mutant	non-codel	Methylated	No combined CNA	Mutant
TCGA.FG.A4MU	58	Male	3.253777534	5.457019647	Gain	Oligoastrocytoma	III	Anaplastic astrocytoma, IDH-wildtype	Mesenchymal-like	CL	321	10.7	0	WT	non-codel	Methylated	Gain chr 7 & loss chr 10	WT
TCGA.FG.A4MW	63	Male	3.129085148	4.740085653	Gain	Oligoastrocytoma	III	Anaplastic astrocytoma, IDH-wildtype	Classic-like	CL	552	18.4	1	WT	non-codel	Methylated	Gain chr 7 & loss chr 10	WT
TCGA.FG.A4MX	47	Male	3.529096294	5.535228712	diploid	Astrocytoma	II	Diffuse astrocytoma, IDH-mutant	G-CIMP-high		561	18.7	0	Mutant	non-codel	Methylated	No combined CNA	Mutant
TCGA.FG.A4MY	44	Female	2.176131252	3.589260133	diploid	Oligoastrocytoma	II	Diffuse astrocytoma, IDH-mutant	G-CIMP-high		711	23.7	0	Mutant	non-codel	Methylated	No combined CNA	Mutant
TCGA.FG.A60J	47	Female	1.774375932	4.079924192	diploid	Oligoastrocytoma	II	Diffuse astrocytoma, IDH-mutant	G-CIMP-high	PN	444	14.8	0	Mutant	non-codel	Methylated	No combined CNA	WT
TCGA.FG.A60K	34	Female	2.107922163	3.503819379	diploid	Oligoastrocytoma	II	Oligodendroglioma, IDH-mutant and 1p/19	Codel	PN	534	17.8	0	Mutant	codel	Methylated	No combined CNA	WT
TCGA.FG.A60L	34	Female	3.294547234	4.056644217	Gain	Astrocytoma	II	Diffuse astrocytoma, IDH-mutant	G-CIMP-high		645	21.5	0	Mutant	non-codel	Methylated	No combined CNA	WT
TCGA.FG.A6IZ	60	Male	3.122225431	3.148478616	diploid	Oligodendroglioma	II	Oligodendroglioma, IDH-mutant and 1p/19	Codel	PN	450	15	0	Mutant	codel	Methylated	No combined CNA	WT
TCGA.FG.A6J1	44	Female	3.332937028	4.451277062	diploid	Oligodendroglioma	II	Oligodendroglioma, IDH-mutant and 1p/19	Codel	PN	492	16.4	0	Mutant	codel	Methylated	No combined CNA	WT
TCGA.FG.A6J3	52	Female	5.13480825	2.574392198	diploid	Astrocytoma	III	Anaplastic astrocytoma, IDH-mutant	G-CIMP-low	ME	669	22.3	0	Mutant	non-codel	Methylated	No combined CNA	WT
TCGA.FG.A70Y	20	Female	3.961197309	3.129909423	diploid	Oligodendroglioma	II	Oligodendroglioma, NOS	G-CIMP-high		855	28.5	0	Mutant	non-codel	Methylated	No combined CNA	Mutant
TCGA.FG.A70Z	53	Female	4.105745116	4.872158957	Gain	Oligoastrocytoma	III	Anaplastic astrocytoma, IDH-wildtype	Mesenchymal-like	CL	324	10.8	0	WT	non-codel	Methylated	Gain chr 7 & loss chr 10	WT
TCGA.FG.A710	50	Female	3.82767678	4.193038384	diploid	Oligodendroglioma	II	Oligodendroglioma, IDH-mutant and 1p/19	Codel	PN	1101	36.7	0	Mutant	codel	Methylated	No combined CNA	WT
TCGA.FG.A711	33	Female	2.41267315	4.641829223	Gain	Oligodendroglioma	II	Oligodendroglioma, NOS	G-CIMP-high	NE	1461	48.7	1	Mutant	non-codel	Unmethylated	No combined CNA	Mutant
TCGA.FG.A713	74	Female	2.969141278	6.303375276	diploid	Oligoastrocytoma	II	Oligodendroglioma, IDH-mutant and 1p/19	Codel	NE	612	20.4	0	Mutant	codel	Methylated	No combined CNA	Mutant
TCGA.FG.A87N	37	Female	2.469885976	4.183232413	diploid	Astrocytoma	III	Anaplastic astrocytoma, IDH-mutant	G-CIMP-low		558	18.6	0	Mutant	non-codel	Methylated	No combined CNA	WT
TCGA.FG.A87Q	61	Female	5.298804923	2.255500733	Gain	Astrocytoma	III	Anaplastic astrocytoma, IDH-wildtype	Classic-like		171	5.7	0	WT	non-codel	Methylated	Gain chr 7 & loss chr 10	WT
TCGA.FN.7833	25	Male	3.492712046	3.86409698	diploid	Oligoastrocytoma	III	Anaplastic astrocytoma, IDH-mutant	G-CIMP-high		825	27.5	0	Mutant	non-codel	Methylated	No combined CNA	Mutant
TCGA.HT.7467	54	Male	1.919111216	4.841490128	diploid	Oligodendroglioma	II	Oligodendroglioma, IDH-mutant and 1p/19	Codel	NE	3	0.1	0	Mutant	codel	Methylated	No combined CNA	WT
TCGA.HT.7468	30	Male	0.837216667	2.516090811	diploid	Oligodendroglioma	III	Anaplastic oligodendroglioma, IDH-mutant	Codel	PN	201	6.7	0	Mutant	codel	Methylated	No combined CNA	WT
TCGA.HT.7469	30	Male	2.741553276	3.369089533	diploid	Oligodendroglioma	III	Anaplastic oligodendroglioma, NOS	PA-like	CL	345	11.5	1	WT	non-codel	Methylated	No combined CNA	Mutant
TCGA.HT.7470	37	Male	2.791418187	5.049482657	diploid	Oligodendroglioma	III	Anaplastic oligodendroglioma, NOS	G-CIMP-high	NE	1203	40.1	1	Mutant	non-codel	Methylated	No combined CNA	Mutant
TCGA.HT.7471	37	Female	2.904503366	3.579168077	diploid	Oligodendroglioma	III	Anaplastic oligodendroglioma, IDH-mutant	Codel	PN	3	0.1	0	Mutant	codel	Methylated	No combined CNA	WT
TCGA.HT.7472	38	Male	2.629916102	1.695013873	diploid	Oligodendroglioma	II	Oligodendroglioma, NOS	G-CIMP-high	PN	0	0	0	Mutant	non-codel	Methylated	No combined CNA	Mutant
TCGA.HT.7473	28	Male	4.708374874	2.997816272	Gain	Oligoastrocytoma	II	Diffuse astrocytoma, IDH-mutant	G-CIMP-high		495	16.5	0	Mutant	non-codel	Unmethylated	No combined CNA	Mutant
TCGA.HT.7474	52	Male	2.708739041	5.330328927	diploid	Oligoastrocytoma	II	Diffuse astrocytoma, IDH-mutant	G-CIMP-high	NE	114	3.8	0	Mutant	non-codel	Methylated	No combined CNA	Mutant
TCGA.HT.7475	67	Male	0.864176238	5.192277031	diploid	Oligoastrocytoma	III	Anaplastic astrocytoma, IDH-mutant	G-CIMP-high	NE	522	17.4	0	Mutant	non-codel	Methylated	No combined CNA	Mutant
TCGA.HT.7476	26	Male	4.585857896	4.761136315	diploid	Astrocytoma	II	Diffuse astrocytoma, IDH-mutant	G-CIMP-high	NE	195	6.5	0	Mutant	non-codel	Methylated	No combined CNA	Mutant
TCGA.HT.7477	62	Male	2.211697545	4.547283241	Gain	Astrocytoma	III	Anaplastic astrocytoma, IDH-mutant	G-CIMP-high	PN	729	24.3	0	Mutant	non-codel	Methylated	No combined CNA	Mutant
TCGA.HT.7478	36	Male	3.003854057	3.640297002	diploid	Astrocytoma	II	Diffuse astrocytoma, IDH-mutant	G-CIMP-high	ME	192	6.4	0	Mutant	non-codel	Unmethylated	No combined CNA	Mutant
TCGA.HT.7479	44	Male	3.143475054	3.39179521	diploid	Astrocytoma	III	Anaplastic astrocytoma, IDH-mutant	G-CIMP-high		1209	40.3	0	Mutant	non-codel	Methylated	No combined CNA	WT
TCGA.HT.7480	33	Male	1.386424705	4.024124611	diploid	Oligodendroglioma	II	Oligodendroglioma, IDH-mutant and 1p/19	Codel	PN	2256	75.2	0	Mutant	codel	Methylated	No combined CNA	WT
TCGA.HT.7481	39	Male	-0.177555361	4.133185377	diploid	Oligodendroglioma	II	Oligodendroglioma, IDH-mutant and 1p/19	Codel	NE	2877	95.9	0	Mutant	codel	Methylated	No combined CNA	WT
TCGA.HT.7482	18	Female	2.798071258	2.67737293	diploid	Oligoastrocytoma	II	Diffuse astrocytoma, IDH-mutant	G-CIMP-high	PN	3210	107	0	Mutant	non-codel	Methylated	No combined CNA	Mutant
TCGA.HT.7483	14	Male	1.208454831	3.917489042	diploid	Oligoastrocytoma	II	Diffuse astrocytoma, IDH-mutant	G-CIMP-high	PN	5184	172.8	0	Mutant	non-codel	Unmethylated	No combined CNA	Mutant
TCGA.HT.7485	42	Male	2.665324546	1.299420514	diploid	Astrocytoma	II	Diffuse astrocytoma, IDH-mutant	G-CIMP-high		120	4	0	Mutant	non-codel	Methylated	No combined CNA	Mutant
TCGA.HT.7601	30	Female	3.767834859	3.064520753	diploid	Astrocytoma	III	Anaplastic astrocytoma, IDH-mutant	G-CIMP-high		150	5	0	Mutant	non-codel	Methylated	No combined CNA	WT
TCGA.HT.7602	21	Male	2.871488844	3.506728872	diploid	Oligodendroglioma	II	Oligodendroglioma, NOS	G-CIMP-high		897	29.9	0	Mutant	non-codel	Methylated	No combined CNA	WT
TCGA.HT.7603	29	Male	3.210373278	6.015698267	diploid	Oligodendroglioma	II	Oligodendroglioma, NOS	G-CIMP-high	NE	696	23.2	0	Mutant	non-codel	Methylated	No combined CNA	Mutant
TCGA.HT.7604	50	Male	1.785843455	2.571506928	Gain	Astrocytoma	II	Diffuse astrocytoma, IDH-mutant	G-CIMP-high	PN	3675	122.5	0	Mutant	non-codel	Methylated	No combined CNA	Mutant
TCGA.HT.7605	38	Male	1.976510281	5.224727182	diploid	Oligodendroglioma	II	Oligodendroglioma, IDH-mutant and 1p/19	Codel	NE	138	4.6	0	Mutant	codel	Methylated	No combined CNA	WT
TCGA.HT.7606	30	Female	2.358846331	3.756201359	diploid	Astrocytoma	II	Diffuse astrocytoma, IDH-mutant	G-CIMP-high	PN	519	17.3	0	Mutant	non-codel	Unmethylated	No combined CNA	WT
TCGA.HT.7607	61	Female	2.286023554	5.468840685	diploid	Astrocytoma	II	Diffuse astrocytoma, IDH-mutant	Codel	NE	96	3.2	1	Mutant	codel	Methylated	No combined CNA	WT
TCGA.HT.7608	61	Male	1.885105754	3.125006307	diploid	Oligoastrocytoma	II	Oligodendroglioma, IDH-mutant and 1p/19	Codel	NE	663	22.1	0	Mutant	codel	Methylated	No combined CNA	WT
TCGA.HT.7609	34	Male	1.040120917	1.925847548	diploid	Oligoastrocytoma	III	Anaplastic astrocytoma, IDH-mutant	G-CIMP-high	PN	1380	46	0	Mutant	non-codel	Methylated	No combined CNA	WT
TCGA.HT.7610	25	Female	1.909465911	5.039230534	Gain	Oligoastrocytoma	II	Diffuse astrocytoma, IDH-mutant	G-CIMP-high	NE	1683	56.1	0	Mutant	non-codel	Methylated	No combined CNA	Mutant
TCGA.HT.7611	36	Male	2.44993108	2.613932346	diploid	Oligoastrocytoma	II	Diffuse astrocytoma, IDH-mutant	G-CIMP-high		1728	57.6	0	Mutant	non-codel	Methylated	No combined CNA	Mutant
TCGA.HT.7616	75	Male	3.794374275	1.887525271	diploid	Oligodendroglioma	III	Anaplastic oligodendroglioma, IDH-mutant	Codel	ME	6	0.2	1	Mutant	codel	Methylated	No combined CNA	WT
TCGA.HT.7620	40	Male	1.382667253	2.494927567	diploid	Oligodendroglioma	III	Anaplastic oligodendroglioma, IDH-mutant	Codel	PN	429	14.3	0	Mutant	codel	Methylated	No combined CNA	WT
TCGA.HT.7676	26	Male	3.548991401	2.529596093	diploid	Oligodendroglioma	II	Oligodendroglioma, NOS	G-CIMP-high	PN	6	0.2	0	Mutant	non-codel	Unmethylated	No combined CNA	Mutant
TCGA.HT.7677	53	Male	1.168834553	3.621008601	diploid	Oligodendroglioma	III	Anaplastic oligodendroglioma, IDH-mutant	Codel	PN	486	16.2	0	Mutant	codel	Methylated	No combined CNA	WT
TCGA.HT.7680	32	Female	2.538935409	4.568676673	diploid	Astrocytoma	II	Diffuse astrocytoma, IDH-wildtype	PA-like		24	0.8	0	WT	non-codel	Unmethylated	No combined CNA	WT
TCGA.HT.7681	29	Female	3.087904069	4.968754331	diploid	Oligoastrocytoma	II	Oligodendroglioma, IDH-mutant and 1p/19	Codel	NE	1341	44.7	0	Mutant	codel	Methylated	No combined CNA	WT
TCGA.HT.7684	58	Male	3.175285063	2.075498403	diploid	Oligoastrocytoma	III	Anaplastic astrocytoma, IDH-mutant	G-CIMP-high		180	6	0	Mutant	non-codel	Methylated	No combined CNA	WT
TCGA.HT.7686	29	Female	3.847395658	2.731748042	diploid	Astrocytoma	III	Anaplastic astrocytoma, IDH-mutant	G-CIMP-high		1281	42.7	0	Mutant	non-codel	Methylated	No combined CNA	Mutant
TCGA.HT.7687	74	Male	1.738594919	5.575039984	diploid	Oligodendroglioma	III	Anaplastic oligodendroglioma, IDH-mutant	Codel	PN	3	0.1	0	Mutant	codel	Methylated	No combined CNA	WT
TCGA.HT.7688	59	Male	1.917966341	5.682306419	Gain	Oligodendroglioma	III	Anaplastic oligodendroglioma, NOS	G-CIMP-high	NE	951	31.7	0	Mutant	non-codel	Methylated	No combined CNA	Mutant
TCGA.HT.7689	58	Female	2.572113556	2.926246174	diploid	Oligodendroglioma	II	Oligodendroglioma, NOS	G-CIMP-high	PN	450	15	0	Mutant	non-codel	Methylated	No combined CNA	Mutant
TCGA.HT.7690	29	Male	3.843431174	4.092816327	diploid	Oligoastrocytoma	III	Anaplastic astrocytoma, IDH-mutant	G-CIMP-high	PN	3	0.1	0	Mutant	non-codel	Methylated	No combined CNA	Mutant
TCGA.HT.7691	31	Female	3.247517426	3.059112962	diploid	Astrocytoma	II	Diffuse astrocytoma, IDH-wildtype	PA-like		3	0.1	0	WT	non-codel	Unmethylated	No combined CNA	WT
TCGA.HT.7692	43	Male	2.634082078	4.299076109	diploid	Oligoastrocytoma	II	Oligodendroglioma, IDH-mutant and 1p/19	Codel	PN	90	3	0	Mutant	codel	Methylated	No combined CNA	WT
TCGA.HT.7693	51	Female	3.228695575	4.049195106	Gain	Oligodendroglioma	II	Oligodendroglioma, NOS	G-CIMP-high		525	17.5	0	Mutant	non-codel	Methylated	No combined CNA	Mutant
TCGA.HT.7694	60	Male	1.921779063	5.426912145	diploid	Oligodendroglioma	III	Anaplastic oligodendroglioma, IDH-mutant	Codel	NE	207	6.9	0	Mutant	codel	Methylated	No combined CNA	WT
TCGA.HT.7695	29	Female	1.825704232	5.070483787	diploid	Oligodendroglioma	II	Oligodendroglioma, IDH-mutant and 1p/19	Codel	NE	435	14.5	0	Mutant	codel	Methylated	No combined CNA	WT
TCGA.HT.7854	62	Male	3.928682992	4.113392127	diploid	Astrocytoma	II	Diffuse astrocytoma, IDH-wildtype	PA-like	NE	1185	39.5	0	WT	non-codel	Unmethylated	No combined CNA	WT
TCGA.HT.7855	39	Male	2.02219024	2.360139496	diploid	Astrocytoma	III	Anaplastic astrocytoma, IDH-mutant	G-CIMP-high	PN	576	19.2	0	Mutant	non-codel	Methylated	No combined CNA	Mutant
TCGA.HT.7856	35	Male	2.593473293	6.229851291	diploid	Oligodendroglioma	III	Anaplastic oligodendroglioma, IDH-mutant	Codel	NE	1173	39.1	0	Mutant	codel	Methylated	No combined CNA	WT
TCGA.HT.7857	24	Female	5.455373254	3.542617131	diploid	Astrocytoma	III	Anaplastic astrocytoma, IDH-wildtype	PA-like	ME	6	0.2	0	WT	non-codel	Unmethylated	No combined CNA	Mutant
TCGA.HT.7858	28	Male	3.441045186	3.391259058	diploid	Astrocytoma	II	Diffuse astrocytoma, IDH-mutant	G-CIMP-high	PN	1518	50.6	0	Mutant	non-codel	Methylated	No combined CNA	Mutant
TCGA.HT.7860	60	Female	3.135091552	3.401193551	Gain	Astrocytoma	III	Anaplastic astrocytoma, IDH-wildtype	Classic-like	CL	15	0.5	0	WT	non-codel	Methylated	Gain chr 7 & loss chr 10	WT
TCGA.HT.7873	29	Male	3.578612712	3.340861316	diploid	Oligoastrocytoma	II	Diffuse astrocytoma, IDH-mutant	G-CIMP-high	PN	708	23.6	0	Mutant	non-codel	Methylated	No combined CNA	Mutant
TCGA.HT.7874	41	Female	1.508834274	5.858232201	diploid	Oligodendroglioma	III	Anaplastic oligodendroglioma, IDH-mutant	Codel	NE	1113	37.1	0	Mutant	codel	Methylated	No combined CNA	WT
TCGA.HT.7875	56	Male	2.17635469	4.353076233	diploid	Oligodendroglioma	II	Oligodendroglioma, IDH-mutant and 1p/19	Codel	PN	9	0.3	0	Mutant	codel	Methylated	No combined CNA	WT
TCGA.HT.7877	20	Female	1.577102485	5.154971988	diploid	Oligodendroglioma	II	Oligodendroglioma, IDH-mutant and 1p/19	Codel	NE	3	0.1	0	Mutant	codel	Methylated	No combined CNA	WT

TCGA.HT.7879	31	Male	3.338481465	3.439370478	diploid	Oligoastrocytoma	III	Anaplastic astrocytoma, IDH-mutant	G-CIMP-high	PN	111	3.7	0	Mutant	non-codel	Methylated	No combined CNA	Mutant
TCGA.HT.7880	30	Male	3.180466161	5.429666173	diploid	Oligoastrocytoma	II	Diffuse astrocytoma, IDH-mutant	G-CIMP-high	NE	159	5.3	0	Mutant	non-codel	Methylated	No combined CNA	Mutant
TCGA.HT.7881	38	Male	2.190203958	5.203306887	diploid	Oligodendroglioma	II	Oligodendroglioma, IDH-mutant and 1p/19	Codel	NE	1065	35.5	0	Mutant	codel	Methylated	No combined CNA	WT
TCGA.HT.7882	66	Male	3.487048947	5.223677393	Gain	Oligodendroglioma	III	Anaplastic oligodendroglioma, NOS	Mesenchymal-like	ME	111	3.7	1	WT	non-codel	Methylated	No combined CNA	WT
TCGA.HT.7884	44	Female	1.932817103	4.396460933	Gain	Astrocytoma	II	Diffuse astrocytoma, IDH-mutant	G-CIMP-high	PN	339	11.3	0	Mutant	non-codel	Methylated	No combined CNA	Mutant
TCGA.HT.7902	30	Female	2.315160433	4.561582756	diploid	Oligoastrocytoma	II	Diffuse astrocytoma, IDH-mutant	G-CIMP-high	NE	942	31.4	0	Mutant	non-codel	Methylated	No combined CNA	Mutant
TCGA.HT.8010	64	Female	1.394349504	5.545551434	diploid	Oligodendroglioma	II	Oligodendroglioma, IDH-mutant and 1p/19	Codel	NE	48	1.6	0	Mutant	codel	Methylated	No combined CNA	WT
TCGA.HT.8011	55	Male	1.979732709	6.199342548	Gain	Astrocytoma	III	Anaplastic astrocytoma, IDH-wildtype	Mesenchymal-like		486	16.2	0	WT	non-codel	Unmethylated	Gain chr 7 & loss chr 10	WT
TCGA.HT.8012	30	Female	2.189824559	4.273560647	diploid	Oligodendroglioma	II	Oligodendroglioma, IDH-mutant and 1p/19	Codel	PN	282	9.4	0	Mutant	codel	Methylated	No combined CNA	WT
TCGA.HT.8013	37	Female	4.741267422	3.529146282	Gain	Oligoastrocytoma	II	Diffuse astrocytoma, IDH-mutant	G-CIMP-high	PN	1905	63.5	1	Mutant	non-codel	Methylated	No combined CNA	Mutant
TCGA.HT.8015	21	Male	4.223878147	6.259377954	diploid	Astrocytoma	II	Diffuse astrocytoma, IDH-wildtype	PA-like	NE	3	0.1	0	WT	non-codel	Unmethylated	No combined CNA	WT
TCGA.HT.8018	40	Female	3.611609136	5.455623127	diploid	Oligoastrocytoma	II	Diffuse astrocytoma, IDH-mutant	G-CIMP-high	NE	1137	37.9	1	Mutant	non-codel	Methylated	No combined CNA	WT
TCGA.HT.8019	34	Female	3.182310918	7.023700026	diploid	Oligodendroglioma	III	Anaplastic oligodendroglioma, NOS	PA-like	NE	990	33	0	WT	non-codel	Unmethylated	No combined CNA	WT
TCGA.HT.8104	51	Female	3.122059733	5.010560554	Gain	Astrocytoma	III	Anaplastic astrocytoma, IDH-wildtype	Classic-like	CL	366	12.2	0	WT	non-codel	Unmethylated	Gain chr 7 & loss chr 10	WT
TCGA.HT.8105	54	Male	1.486765851	4.546456184	diploid	Oligodendroglioma	III	Anaplastic oligodendroglioma, IDH-mutant	Codel	PN	186	6.2	0	Mutant	codel	Methylated	No combined CNA	WT
TCGA.HT.8106	53	Male	5.325789346	4.057432942	diploid	Astrocytoma	III	Anaplastic astrocytoma, IDH-mutant	G-CIMP-high	ME	3	0.1	0	Mutant	non-codel	Methylated	No combined CNA	WT
TCGA.HT.8107	62	Male	0.913569218	6.386914386	diploid	Oligodendroglioma	II	Oligodendroglioma, NOS	PA-like	NE	15	0.5	0	WT	non-codel	Methylated	No combined CNA	WT
TCGA.HT.8108	26	Female	2.997671777	3.042294126	Gain	Oligodendroglioma	II	Oligodendroglioma, NOS	G-CIMP-high	PN	75	2.5	0	Mutant	non-codel	Methylated	No combined CNA	Mutant
TCGA.HT.8109	64	Male	2.915616504	4.322822289	diploid	Oligodendroglioma	III	Anaplastic oligodendroglioma, IDH-mutant	Codel	NE	168	5.6	0	Mutant	codel	Methylated	No combined CNA	WT
TCGA.HT.8110	57	Male	4.851973859	7.033594793	Gain	Astrocytoma	III	Anaplastic astrocytoma, IDH-wildtype	Mesenchymal-like	ME	414	13.8	0	WT	non-codel	Methylated	Gain chr 7 & loss chr 10	WT
TCGA.HT.8111	32	Male	3.763783355	4.122681001	diploid	Oligoastrocytoma	III	Anaplastic astrocytoma, IDH-mutant	G-CIMP-high	PN	6	0.2	0	Mutant	non-codel	Methylated	No combined CNA	WT
TCGA.HT.8113	49	Female	2.312316654	6.54934727	diploid	Oligodendroglioma	II	Oligodendroglioma, NOS	Codel	NE	888	29.6	0	Mutant	non-codel	Methylated	No combined CNA	WT
TCGA.HT.8114	36	Male	4.368272468	2.82535825	Gain	Oligoastrocytoma	III	Anaplastic astrocytoma, IDH-mutant	G-CIMP-high		1026	34.2	0	Mutant	non-codel	Methylated	No combined CNA	Mutant
TCGA.HT.8558	29	Female	3.225984379	6.39304889	NA	Oligodendroglioma	II	Oligodendroglioma, NOS	PA-like	NE	411	13.7	0	WT	non-codel	Unmethylated		WT
TCGA.HT.8563	30	Female	4.999702413	2.944764761	diploid	Astrocytoma	III	Anaplastic astrocytoma, IDH-mutant	G-CIMP-high	ME	849	28.3	0	Mutant	non-codel	Unmethylated	No combined CNA	Mutant
TCGA.HT.8564	47	Male	3.235635166	5.91179942	Gain	Astrocytoma	III	Anaplastic astrocytoma, IDH-wildtype	PA-like	NE	471	15.7	0	WT	non-codel	Unmethylated	No combined CNA	WT
TCGA.HT.A4DS	55	Female	5.951042205	6.168094423	Gain	Astrocytoma	III	Anaplastic astrocytoma, IDH-wildtype	Mesenchymal-like	CL	6	0.2	0	WT	non-codel	Unmethylated	No combined CNA	WT
TCGA.HT.A4DV	51	Female	1.887525271	5.00162212	diploid	Oligodendroglioma	III	Anaplastic oligodendroglioma, IDH-mutant	Codel	NE	780	26	0	Mutant	codel	Methylated	No combined CNA	WT
TCGA.HT.A5R5	33	Female	3.034972791	4.915367923	Gain	Oligodendroglioma	II	Oligodendroglioma, NOS	G-CIMP-high	NE	3	0.1	0	Mutant	non-codel	Methylated	No combined CNA	Mutant
TCGA.HT.A5R7	33	Female	2.265676925	5.600341125	NA	Astrocytoma	III	Anaplastic astrocytoma, IDH-mutant	G-CIMP-high	NE	606	20.2	0	Mutant	non-codel	Methylated	No combined CNA	Mutant
TCGA.HT.A5R9	48	Female	0.889317778	5.010457613	Gain	Oligodendroglioma	III	Anaplastic oligodendroglioma, IDH-mutant	Codel	PN	3	0.1	0	Mutant	codel	Methylated	No combined CNA	WT
TCGA.HT.A5RA	65	Female	3.977783634	3.399116393	Gain	Astrocytoma	III	Anaplastic astrocytoma, IDH-wildtype	Mesenchymal-like	CL	822	27.4	0	WT	non-codel	Unmethylated	No combined CNA	WT
TCGA.HT.A5RB	24	Male	2.644456226	4.647809523	diploid	Astrocytoma	II	Diffuse astrocytoma, IDH-mutant	G-CIMP-high	PN	3	0.1	0	Mutant	non-codel	Methylated	No combined CNA	Mutant
TCGA.HT.A5RC	70	Female	3.31314425	3.435068474	Gain	Astrocytoma	III	Anaplastic astrocytoma, IDH-wildtype	Classic-like	CL	159	5.3	1	WT	non-codel	Unmethylated	Gain chr 7 & loss chr 10	WT
TCGA.HT.A614	47	Male	2.966448925	4.348763557	diploid	Oligoastrocytoma	II	Diffuse astrocytoma, IDH-mutant	G-CIMP-high	PN	81	2.7	0	Mutant	non-codel	Methylated	No combined CNA	Mutant
TCGA.HT.A615	38	Female	4.323103412	3.67226692	diploid	Oligodendroglioma	II	Oligodendroglioma, IDH-mutant and 1p/19	Codel	PN	507	16.9	0	Mutant	codel	Methylated	No combined CNA	WT
TCGA.HT.A616	36	Female	2.120517834	4.775129577	Gain	Astrocytoma	II	Diffuse astrocytoma, IDH-mutant	G-CIMP-high	NE	54	1.8	0	Mutant	non-codel	Methylated	No combined CNA	Mutant
TCGA.HT.A617	47	Male	4.683348369	4.727288797	Gain	Oligodendroglioma	II	Oligodendroglioma, NOS	Classic-like	CL	483	16.1	0	WT	non-codel	Unmethylated	Gain chr 7 & loss chr 10	WT
TCGA.HT.A618	37	Female	4.146027898	3.498927371	NA	Astrocytoma	III	Anaplastic astrocytoma, IDH-mutant	G-CIMP-high		504	16.8	0	Mutant	non-codel	Methylated	No combined CNA	Mutant
TCGA.HT.A619	51	Female	-0.069906106	2.548781844	Gain	Oligodendroglioma	III	Anaplastic oligodendroglioma, IDH-mutant	Codel	PN	642	21.4	0	Mutant	codel	Methylated	No combined CNA	WT
TCGA.HT.A61A	20	Female	4.083715458	6.565737296	NA	Oligodendroglioma	II	Oligodendroglioma, NOS	G-CIMP-high		192	6.4	0	Mutant	non-codel	Methylated		WT
TCGA.HT.A61B	22	Male	4.664869631	4.243128274	diploid	Astrocytoma	III	Anaplastic astrocytoma, IDH-mutant	G-CIMP-high		525	17.5	0	Mutant	non-codel	Methylated	No combined CNA	WT
TCGA.HT.A61C	66	Male	4.413966352	6.530056692	Gain	Oligodendroglioma	III	Anaplastic oligodendroglioma, NOS	Mesenchymal-like		528	17.6	1	WT	non-codel	Unmethylated	Gain chr 7 & loss chr 10	WT
TCGA.HT.A74H	62	Male	4.67827477	8.380711269	Gain	Astrocytoma	III	Anaplastic astrocytoma, IDH-wildtype	Mesenchymal-like	NE	72	2.4	0	WT	non-codel	Unmethylated	Gain chr 7 & loss chr 10	WT
TCGA.HT.A74J	33	Male	2.713255902	5.838447593	Gain	Oligoastrocytoma	II	Diffuse astrocytoma, IDH-mutant	G-CIMP-high	PN	315	10.5	0	Mutant	non-codel	Unmethylated	No combined CNA	Mutant
TCGA.HT.A74K	58	Female	4.320520781	3.724803045	Gain	Oligodendroglioma	III	Anaplastic oligodendroglioma, IDH-mutant	Codel	PN	459	15.3	0	Mutant	codel	Methylated	No combined CNA	WT
TCGA.HT.A74L	22	Female	1.541316585	2.349761568	Gain	Oligoastrocytoma	II	Oligodendroglioma, IDH-mutant and 1p/19	Codel	PN	333	11.1	0	Mutant	codel	Methylated	No combined CNA	WT
TCGA.HT.A74O	34	Male	5.257365212	3.544967523	Gain	Astrocytoma	III	Anaplastic astrocytoma, IDH-mutant	G-CIMP-high		3	0.1	0	Mutant	non-codel	Methylated	No combined CNA	Mutant
TCGA.HW.7486	37	Male	2.858378925	2.896736847	diploid	Oligodendroglioma	II	Oligodendroglioma, IDH-mutant and 1p/19	Codel	PN	1698	56.6	0	Mutant	codel	Methylated	No combined CNA	WT
TCGA.HW.7487	39	Male	-1	5.274533772	diploid	Oligodendroglioma	II	Oligodendroglioma, IDH-mutant and 1p/19	Codel	NE	402	13.4	0	Mutant	codel	Methylated	No combined CNA	WT
TCGA.HW.7489	38	Male	1.716112942	5.565481414	diploid	Oligoastrocytoma	II	Diffuse astrocytoma, IDH-mutant	G-CIMP-high	NE	1245	41.5	1	Mutant	non-codel	Methylated	No combined CNA	Mutant
TCGA.HW.7490	41	Male	2.857762087	2.188337619	diploid	Astrocytoma	II	Diffuse astrocytoma, IDH-mutant	G-CIMP-high		1368	45.6	0	Mutant	non-codel	Methylated	No combined CNA	Mutant
TCGA.HW.7491	35	Male	0.454070583	2.339536494	diploid	Oligodendroglioma	II	Oligodendroglioma, IDH-mutant and 1p/19	Codel	PN	2049	68.3	0	Mutant	codel	Unmethylated	No combined CNA	WT
TCGA.HW.7493	40	Female	2.505179567	4.62862197	diploid	Astrocytoma	II	Diffuse astrocytoma, NOS		NE	2079	69.3	0		non-codel	Methylated	No combined CNA	
TCGA.HW.7495	45	Female	1.983166916	4.743999591	Gain	Oligodendroglioma	II	Oligodendroglioma, IDH-mutant and 1p/19	Codel	NE	1062	35.4	0	Mutant	codel	Methylated	No combined CNA	WT
TCGA.HW.8319	34	Female	2.206393169	4.737984138	diploid	Astrocytoma	III	Anaplastic astrocytoma, IDH-mutant	G-CIMP-high	PN	1194	39.8	1	Mutant	non-codel	Unmethylated	No combined CNA	Mutant
TCGA.HW.8320	36	Male	2.335997677	3.081390762	diploid	Astrocytoma	III	Anaplastic astrocytoma, IDH-mutant	G-CIMP-high	PN	1200	40	0	Mutant	non-codel	Methylated	No combined CNA	WT
TCGA.HW.8321	31	Male	0.556797247	3.833740194	diploid	Astrocytoma	III	Anaplastic astrocytoma, IDH-mutant	G-CIMP-high	PN	1275	42.5	0	Mutant	non-codel	Methylated	No combined CNA	WT
TCGA.HW.8322	39	Male	2.001694172	5.180744616	Gain	Oligodendroglioma	II	Oligodendroglioma, IDH-mutant and 1p/19	Codel	PN	186	6.2	0	Mutant	codel	Methylated	No combined CNA	WT
TCGA.HW.A5KJ	68	Male	0.994072791	4.671723626	Gain	Oligodendroglioma	III	Anaplastic oligodendroglioma, IDH-mutant	Codel	PN	318	10.6	0	Mutant	codel	Methylated	No combined CNA	WT
TCGA.HW.A5KK	64	Male	4.871986531	7.065150056	diploid	Astrocytoma	III	Anaplastic astrocytoma, IDH-wildtype	Mesenchymal-like	CL	384	12.8	1	WT	non-codel	Methylated	No combined CNA	WT
TCGA.HW.A5KL	42	Female	2.611361264	2.422959548	diploid	Astrocytoma	II	Diffuse astrocytoma, IDH-mutant	G-CIMP-high	PN	624	20.8	0	Mutant	non-codel	Methylated	No combined CNA	Mutant
TCGA.HW.A5KM	35	Male	5.922000368	1.509493166	Gain	Astrocytoma	II	Diffuse astrocytoma, IDH-mutant	G-CIMP-high	ME	621	20.7	0	Mutant	non-codel	Methylated	No combined CNA	WT
TCGA.IK.7675	43	Male	2.30591217	1.861399611	diploid	Oligodendroglioma	II	Oligodendroglioma, NOS	G-CIMP-high	PN	570	19	1	Mutant	non-codel	Methylated	No combined CNA	Mutant
TCGA.IK.8125	62	Male	2.443261849	4.296200416	diploid	Oligoastrocytoma	III	Anaplastic oligodendroglioma, IDH-mutant	Codel	NE	1284	42.8	0	Mutant	codel	Methylated	No combined CNA	WT
TCGA.KT.A74X	26	Male	2.26447638	2.796722334	diploid	Oligoastrocytoma	III	Anaplastic oligodendroglioma, IDH-mutant	Codel	PN	432	14.4	0	Mutant	codel	Methylated	No combined CNA	WT
TCGA.KT.A7W1	45	Female	3.463334726	3.493570484	Gain	Astrocytoma	III	Anaplastic astrocytoma, IDH-wildtype	Classic-like	CL	432	14.4	0	WT	non-codel	Methylated	Gain chr 7 & loss chr 10	WT
TCGA.P5.A5ET	27	Male	1.521804562	4.550703696	diploid	Oligodendroglioma	II	Oligodendroglioma, IDH-mutant and 1p/19	Codel	PN	-9	-0.3	0	Mutant	codel	Methylated	No combined CNA	WT
TCGA.P5.A5EU	35	Male	4.919111216	6.862926178	diploid	Astrocytoma	III	Anaplastic astrocytoma, IDH-mutant	G-CIMP-low		0	0	0	Mutant	non-codel	Unmethylated	No combined CNA	Mutant
TCGA.P5.A5EV	39	Male	3.096177502	3.666995128	Gain	Astrocytoma	II	Diffuse astrocytoma, IDH-mutant	G-CIMP-high	PN	6	0.2	0	Mutant	non-codel	Methylated	No combined CNA	Mutant
TCGA.P5.A5EW	20	Female	2.337397118	3.185660431	diploid	Astrocytoma	II	Diffuse astrocytoma, IDH-mutant	G-CIMP-high	PN	201	6.7	0	Mutant	non-codel	Methylated	No combined CNA	Mutant
TCGA.P5.A5EX	41	Female	4.218866394	3.019862007	diploid	Oligodendroglioma	III	Anaplastic oligodendroglioma, IDH-mutant	Codel	ME	240	8	0	Mutant	codel	Methylated	No combined CNA	WT
TCGA.P5.A5EY	21	Male	2.485941983	5.220155799	diploid	Astrocytoma	II	Diffuse astrocytoma, IDH-wildtype	PA-like	NE	72	2.4	0	WT	non-codel	Unmethylated	No combined CNA	WT
TCGA.P5.A5EZ	39	Male	4.13651133	4.170758318	Gain	Astrocytoma	III	Anaplastic astrocytoma, IDH-mutant	G-CIMP-high	PN	69	2.3	0	Mutant	non-codel	Methylated	No combined CNA	WT
TCGA.P5.A5F0	33	Male	2.565913949	5.065399977	Gain	Oligodendroglioma	II	Oligodendroglioma, IDH-mutant and 1p/19	Codel	NE	123	4.1	0	Mutant	codel	Methylated	No combined CNA	WT
TCGA.P5.A5F1	30	Male	4.688639172	2.536202137	Gain	Astrocytoma	II	Diffuse astrocytoma, IDH-mutant	G-CIMP-high		177	5.9	0	Mutant	non-codel	Unmethylated	No combined CNA	Mutant
TCGA.P5.A5F2	36	Female	1.95069236	3.706895186	diploid	Astrocytoma	II	Diffuse astrocytoma, IDH-mutant	G-CIMP-high	NE	228	7.6	0	Mutant	non-codel	Methylated	No combined CNA	Mutant
TCGA.P5.A5F4	35	Female	3.0422591	5.347612511	diploid	Oligodendroglioma	III	Anaplastic oligodendroglioma, NOS	G-CIMP-high	NE	24	0.8	0	Mutant	non-codel	Methylated	No combined CNA	WT
TCGA.P5.A5F6	26	Male	1.815288501	3.125088989	diploid	Oligodendroglioma	II	Oligodendroglioma, NOS	PA-like	ME	39	1.3	0	WT	non-codel	Unmethylated	No combined CNA	WT
TCGA.P5.A72U	71	Female	3.918090413	5.641410192	Gain	Oligodendroglioma	III	Anaplastic oligodendroglioma, NOS	Classic-like	CL	6	0.2	0	WT	non-codel	Methylated	Gain chr 7 & loss chr 10	WT
TCGA.P5.A72W	35	Male	3.476757543	2.784483044	diploid	Astrocytoma	III	Anaplastic astrocytoma, IDH-mutant	G-CIMP-high	PN	312	10.4	0	Mutant	non-codel	Unmethylated	No combined CNA	Mutant
TCGA.P5.A72X	21	Male	2.728791253	3.822597597	diploid	Astrocytoma	III	Anaplastic astrocytoma, IDH-mutant	G-CIMP-high		396	13.2	0	Mutant	non-codel	Methylated	No combined CNA	WT
TCGA.P5.A72Z	64	Female	1.359296259	2.198462722	diploid	Oligodendroglioma	III	Anaplastic oligodendroglioma, IDH-mutant	Codel	PN	348	11.6	0	Mutant	codel	Methylated	No combined CNA	WT
TCGA.P5.A730	22	Male	2.518056679	2.841631017	diploid	Oligoastrocytoma	III	Anaplastic oligodendroglioma, IDH-mutant	Codel		327	10.9	0	Mutant	codel	Methylated	No combined CNA	WT
TCGA.P5.A731	59	Female	3.003386356	5.807293091	Gain	Oligoastrocytoma	II	Diffuse astrocytoma, IDH-mutant	G-CIMP-high	NE	270	9	0	Mutant	non-codel	Methylated	No combined CNA	WT
TCGA.P5.A733	52	Female	2.424680275	4.011236202	Gain	Astrocytoma	II	Diffuse astrocytoma, IDH-mutant	G-CIMP-high		408	13.6	0	Mutant	non-codel	Methylated	No combined CNA	Mutant
TCGA.P5.A735	38	Female	2.503425066	2.62396048	diploid	Astrocytoma	II	Diffuse astrocytoma, IDH-mutant	G-CIMP-high	PN	288	9.6	0	Mutant	non-codel	Methylated	No combined CNA	Mutant
TCGA.P5.A736	44	Female	5.354762445	5.795278626	Gain	Astrocytoma	III	Diffuse astrocytoma, IDH-mutant	G-CIMP-high		228	7.6	0	Mutant	non-codel	Methylated	No combined CNA	Mutant

TCGA.P5.A737	47	Male	2.441536597	3.285076651	diploid	Oligoastrocytoma	II	Oligodendroglioma, IDH-mutant and 1p/19	Codel	PN	366	12.2	0	Mutant	codel	Methylated	No combined CNA	WT
TCGA.P5.A77W	37	Female	1.69968488	2.778292696	diploid	Oligoastrocytoma	III	Anaplastic oligodendroglioma, IDH-mutant	Codel	PN	576	19.2	0	Mutant	codel	Methylated	No combined CNA	WT
TCGA.P5.A77X	55	Female	1.746441765	5.015452966	diploid	Oligoastrocytoma	II	Oligodendroglioma, IDH-mutant and 1p/19	Codel	NE	105	3.5	0	Mutant	codel	Methylated	No combined CNA	WT
TCGA.P5.A780	44	Female	1.857025515	3.414135533	diploid	Astrocytoma	III	Anaplastic astrocytoma, IDH-mutant	G-CIMP-high	PN	72	2.4	0	Mutant	non-codel	Methylated	No combined CNA	WT
TCGA.P5.A781	34	Female	2.309496363	4.478382962	diploid	Astrocytoma	III	Anaplastic astrocytoma, IDH-mutant	Codel	NE	132	4.4	0	Mutant	codel	Methylated	No combined CNA	WT
TCGA.QH.A65R	38	Female	0.802234584	4.507084215	diploid	Oligodendroglioma	III	Anaplastic oligodendroglioma, IDH-mutant	Codel	PN	456	15.2	0	Mutant	codel	Methylated	No combined CNA	WT
TCGA.QH.A65S	32	Female	3.079958315	2.800910251	diploid	Oligoastrocytoma	II	Diffuse astrocytoma, IDH-mutant	G-CIMP-high	PN	75	2.5	0	Mutant	non-codel	Methylated	No combined CNA	Mutant
TCGA.QH.A65V	43	Female	2.932193488	4.723045172	diploid	Oligodendroglioma	II	Oligodendroglioma, IDH-mutant and 1p/19	Codel	NE	378	12.6	0	Mutant	codel	Methylated	No combined CNA	WT
TCGA.QH.A65X	28	Female	3.533052459	4.031148138	diploid	Oligoastrocytoma	III	Anaplastic oligodendroglioma, IDH-mutant	Codel		474	15.8	0	Mutant	codel	Methylated	No combined CNA	WT
TCGA.QH.A65Z	54	Male	0.857423709	3.575965753	diploid	Oligodendroglioma	II	Oligodendroglioma, IDH-mutant and 1p/19	Codel	PN	420	14	0	Mutant	codel	Methylated	No combined CNA	WT
TCGA.QH.A6CS	41	Male	3.834761772	6.113329611	Gain	Astrocytoma	III	Anaplastic astrocytoma, IDH-wildtype	PA-like		579	19.3	0	WT	non-codel	Unmethylated	Gain chr 7 & loss chr 10	WT
TCGA.QH.A6CU	52	Female	3.67158777	4.760806423	Gain	Oligodendroglioma	III	Anaplastic oligodendroglioma, IDH-mutant	Codel		444	14.8	0	Mutant	codel	Methylated	No combined CNA	WT
TCGA.QH.A6CV	51	Male	4.81275491	6.869318695	Gain	Oligoastrocytoma	III	Anaplastic astrocytoma, IDH-wildtype	Classic-like	CL	435	14.5	0	WT	non-codel	Unmethylated	Gain chr 7 & loss chr 10	WT
TCGA.QH.A6CW	43	Male	2.184756353	3.28036249	diploid	Oligoastrocytoma	III	Anaplastic astrocytoma, IDH-mutant	G-CIMP-high		408	13.6	0	Mutant	non-codel	Methylated	No combined CNA	Mutant
TCGA.QH.A6CX	66	Male	5.066390618	4.030636241	Gain	Astrocytoma	II	Diffuse astrocytoma, IDH-wildtype	Mesenchymal-like	CL	366	12.2	1	WT	non-codel	Unmethylated	No combined CNA	WT
TCGA.QH.A6CY	38	Male	2.272351716	4.751447653	diploid	Oligoastrocytoma	III	Anaplastic oligodendroglioma, IDH-mutant	Codel	NE	66	2.2	0	Mutant	codel	Methylated	No combined CNA	WT
TCGA.QH.A6CZ	38	Male	2.165333137	4.295069109	diploid	Oligoastrocytoma	II	Oligodendroglioma, IDH-mutant and 1p/19	Codel	PN	276	9.2	0	Mutant	codel	Methylated	No combined CNA	WT
TCGA.QH.A6X3	27	Male	1.613908779	2.965525731	Gain	Oligoastrocytoma	II	Diffuse astrocytoma, IDH-mutant	G-CIMP-high	PN	309	10.3	0	Mutant	non-codel	Methylated	No combined CNA	Mutant
TCGA.QH.A6X4	47	Male	3.096801582	4.025081964	diploid	Oligoastrocytoma	III	Anaplastic oligodendroglioma, IDH-mutant	Codel	PN	435	14.5	0	Mutant	codel	Methylated	No combined CNA	WT
TCGA.QH.A6X5	58	Female	2.265916914	2.630754919	diploid	Oligoastrocytoma	II	Oligodendroglioma, IDH-mutant and 1p/19	Codel	PN	489	16.3	0	Mutant	codel	Methylated	No combined CNA	WT
TCGA.QH.A6X8	56	Female	3.586596629	2.653656238	diploid	Oligodendroglioma	III	Anaplastic oligodendroglioma, IDH-mutant	Codel	PN	501	16.7	0	Mutant	codel	Methylated	No combined CNA	WT
TCGA.QH.A6X9	73	Female	1.714663152	4.144209561	Gain	Oligodendroglioma	II	Oligodendroglioma, NOS	G-CIMP-high	PN	57	1.9	0	Mutant	non-codel	Methylated	No combined CNA	Mutant
TCGA.QH.A6XA	23	Female	1.855750557	3.439889048	Gain	Oligoastrocytoma	II	Diffuse astrocytoma, IDH-mutant	G-CIMP-high	PN	495	16.5	0	Mutant	non-codel	Methylated	No combined CNA	Mutant
TCGA.QH.A6XC	48	Male	3.362932711	5.405202744	Gain	Astrocytoma	III	Anaplastic astrocytoma, IDH-wildtype	Classic-like	CL	150	5	0	WT	non-codel	Methylated	Gain chr 7 & loss chr 10	WT
TCGA.QH.A86X	33	Male	2.035905384	4.41719767	diploid	Oligodendroglioma	II	Oligodendroglioma, IDH-mutant and 1p/19	Codel		333	11.1	0	Mutant	codel	Methylated	No combined CNA	WT
TCGA.QH.A870	38	Female	2.008702008	3.175205209	Gain	Oligoastrocytoma	III	Anaplastic astrocytoma, IDH-mutant	G-CIMP-high		390	13	0	Mutant	non-codel	Methylated	No combined CNA	Mutant
TCGA.R8.A6MK	40	Male	2.697240048	3.905081284	diploid	Oligodendroglioma	II	Oligodendroglioma, IDH-mutant and 1p/19	Codel		2664	88.8	0	Mutant	codel	Methylated	No combined CNA	WT
TCGA.R8.A6ML	52	Male	0.691891445	3.836621006	diploid	Oligodendroglioma	III	Anaplastic oligodendroglioma, IDH-mutant	Codel	PN	2820	94	0	Mutant	codel	Methylated	No combined CNA	WT
TCGA.R8.A6MO	53	Female	3.831076648	2.051928885	diploid	Oligodendroglioma	II	Oligodendroglioma, IDH-mutant and 1p/19	Codel	PN	978	32.6	0	Mutant	codel	Methylated	No combined CNA	WT
TCGA.R8.A6YH	NA	NA	2.86985167	3.546005424	diploid	Astrocytoma	NA	NA	G-CIMP-high	PN		0		Mutant	non-codel	Unmethylated	No combined CNA	Mutant
TCGA.R8.A73M	48	Female	1.420886575	5.243219692	diploid	Oligodendroglioma	II	Oligodendroglioma, IDH-mutant and 1p/19	Codel	PN	1782	59.4	0	Mutant	codel	Methylated	No combined CNA	WT
TCGA.RY.A83X	46	Female	2.457804391	2.984425295	diploid	Oligodendroglioma	II	Oligodendroglioma, IDH-mutant and 1p/19	Codel		927	30.9	0	Mutant	codel	Methylated	No combined CNA	WT
TCGA.RY.A83Y	45	Male	3.142250043	3.4668881	diploid	Oligodendroglioma	II	Oligodendroglioma, IDH-mutant and 1p/19	Codel		165	5.5	0	Mutant	codel	Methylated	No combined CNA	WT
TCGA.RY.A83Z	54	Female	3.648891056	4.309620048	diploid	Astrocytoma	III	Anaplastic astrocytoma, IDH-mutant	G-CIMP-low		297	9.9	0	Mutant	non-codel	Methylated	No combined CNA	Mutant
TCGA.RY.A840	47	Male	2.34848739	1.222186307	diploid	Oligodendroglioma	III	Anaplastic oligodendroglioma, IDH-mutant	Codel		843	28.1	0	Mutant	codel	Methylated	No combined CNA	WT
TCGA.RY.A843	30	Male	3.098369023	4.440267885	diploid	Astrocytoma	III	Anaplastic astrocytoma, IDH-mutant	G-CIMP-high		63	2.1	0	Mutant	non-codel	Methylated	No combined CNA	WT
TCGA.RY.A845	40	Female	3.072363095	3.640158155	Gain	Oligoastrocytoma	II	Diffuse astrocytoma, IDH-mutant	G-CIMP-high		588	19.6	0	Mutant	non-codel	Methylated	No combined CNA	WT
TCGA.RY.A847	45	Male	3.899243316	4.638693657	diploid	Oligodendroglioma	II	Oligodendroglioma, IDH-mutant and 1p/19	Codel		921	30.7	0	Mutant	codel	Methylated	No combined CNA	WT
TCGA.S9.A6TS	48	Female	3.150137204	4.212063451	NA	Astrocytoma	III	Anaplastic astrocytoma, IDH-mutant	G-CIMP-high	NE	1866	62.2	1	Mutant	non-codel	Methylated	No combined CNA	Mutant
TCGA.S9.A6TU	38	Male	2.834286474	3.50315789	diploid	Astrocytoma	II	Diffuse astrocytoma, IDH-mutant	G-CIMP-high	PN	2613	87.1	0	Mutant	non-codel	Methylated	No combined CNA	WT
TCGA.S9.A6TV	50	Male	4.17270352	4.169612384	diploid	Oligoastrocytoma	III	Anaplastic astrocytoma, IDH-mutant	G-CIMP-high	ME	564	18.8	0	Mutant	non-codel	Methylated	No combined CNA	Mutant
TCGA.S9.A6TW	40	Male	-0.012315322	2.665279061	diploid	Oligodendroglioma	III	Anaplastic oligodendroglioma, IDH-mutant	Codel		1233	41.1	0	Mutant	codel	Methylated	No combined CNA	WT
TCGA.S9.A6TX	46	Male	2.065916918	3.640447406	diploid	Oligodendroglioma	III	Anaplastic oligodendroglioma, IDH-mutant	Codel	PN	843	28.1	0	Mutant	codel	Methylated	No combined CNA	WT
TCGA.S9.A6TY	50	Male	0.392097567	4.151412369	Gain	Oligodendroglioma	II	Oligodendroglioma, IDH-mutant and 1p/19	Codel	PN	1062	35.4	0	Mutant	codel	Methylated	No combined CNA	WT
TCGA.S9.A6TZ	39	Female	2.440899059	3.834043709	diploid	Astrocytoma	II	Diffuse astrocytoma, IDH-mutant	G-CIMP-high	PN	1104	36.8	0	Mutant	non-codel	Methylated	No combined CNA	WT
TCGA.S9.A6U0	46	Male	4.228987957	4.802213901	Gain	Astrocytoma	III	Anaplastic astrocytoma, IDH-wildtype	Mesenchymal-like	ME	732	24.4	1	WT	non-codel	Methylated	Gain chr 7 & loss chr 10	WT
TCGA.S9.A6U1	22	Female	3.14891784	2.468296574	diploid	Astrocytoma	III	Anaplastic astrocytoma, IDH-mutant	G-CIMP-high	PN	774	25.8	0	Mutant	non-codel	Methylated	No combined CNA	Mutant
TCGA.S9.A6U2	48	Female	2.250749397	2.944539894	diploid	Oligodendroglioma	II	Oligodendroglioma, IDH-mutant and 1p/19	Codel	PN	897	29.9	0	Mutant	codel	Methylated	No combined CNA	WT
TCGA.S9.A6U5	33	Male	3.670874319	3.655981439	diploid	Astrocytoma	II	Diffuse astrocytoma, IDH-mutant	Codel	PN	978	32.6	0	Mutant	codel	Methylated	No combined CNA	WT
TCGA.S9.A6U6	28	Male	3.02185279	4.473462236	diploid	Astrocytoma	III	Anaplastic astrocytoma, IDH-mutant	G-CIMP-high	NE	1053	35.1	0	Mutant	non-codel	Methylated	No combined CNA	Mutant
TCGA.S9.A6U8	24	Male	4.357052253	3.46920874	diploid	Astrocytoma	II	Diffuse astrocytoma, IDH-mutant	G-CIMP-high	PN	2946	98.2	1	Mutant	non-codel	Methylated	No combined CNA	Mutant
TCGA.S9.A6U9	36	Male	3.197660985	4.369710751	diploid	Astrocytoma	III	Anaplastic astrocytoma, IDH-mutant	G-CIMP-high		2877	95.9	0	Mutant	non-codel	Methylated	No combined CNA	Mutant
TCGA.S9.A6UA	66	Male	3.708893509	4.454840486	diploid	Astrocytoma	III	Anaplastic astrocytoma, IDH-wildtype	PA-like	ME	174	5.8	1	WT	non-codel	Methylated	No combined CNA	Mutant
TCGA.S9.A6UB	52	Male	0.855750557	3.683763816	diploid	Oligodendroglioma	II	Oligodendroglioma, IDH-mutant and 1p/19	Codel	PN	3711	123.7	0	Mutant	codel	Methylated	No combined CNA	WT
TCGA.S9.A6WD	58	Male	2.545671894	3.813586254	diploid	Oligodendroglioma	III	Anaplastic oligodendroglioma, IDH-mutant	Codel	PN	2256	75.2	0	Mutant	codel	Methylated	No combined CNA	WT
TCGA.S9.A6WE	34	Male	2.444932049	2.972692654	diploid	Oligodendroglioma	II	Oligodendroglioma, IDH-mutant and 1p/19	Codel	PN	4056	135.2	0	Mutant	codel	Methylated	No combined CNA	WT
TCGA.S9.A6WG	31	Male	3.619154735	5.619098964	diploid	Astrocytoma	III	Anaplastic astrocytoma, IDH-mutant	G-CIMP-high	ME	2565	85.5	0	Mutant	non-codel	Methylated	No combined CNA	Mutant
TCGA.S9.A6WH	73	Female	1.360027093	2.560666049	diploid	Oligoastrocytoma	II	Oligodendroglioma, IDH-mutant and 1p/19	Codel	PN	1158	38.6	0	Mutant	codel	Methylated	No combined CNA	WT
TCGA.S9.A6WI	56	Female	2.224626867	6.516330387	diploid	Oligoastrocytoma	II	Diffuse astrocytoma, IDH-mutant	G-CIMP-high	NE	2550	85	0	Mutant	non-codel	Methylated	No combined CNA	WT
TCGA.S9.A6WL	52	Male	2.448742405	4.822949339	diploid	Astrocytoma	III	Anaplastic astrocytoma, IDH-mutant	Codel	NE	933	31.1	0	Mutant	codel	Methylated	No combined CNA	WT
TCGA.S9.A6WM	59	Female	3.100960833	6.539974556	Gain	Astrocytoma	III	Anaplastic astrocytoma, IDH-wildtype	Mesenchymal-like	CL	597	19.9	0	WT	non-codel	Unmethylated	Gain chr 7 & loss chr 10	WT
TCGA.S9.A6WN	38	Female	4.456924398	2.30873947	diploid	Astrocytoma	III	Anaplastic astrocytoma, IDH-mutant	Codel	ME	795	26.5	0	Mutant	codel	Methylated	No combined CNA	WT
TCGA.S9.A6WO	29	Male	1.912611546	3.272082927	Gain	Astrocytoma	II	Diffuse astrocytoma, IDH-mutant	G-CIMP-high	PN	558	18.6	0	Mutant	non-codel	Methylated	No combined CNA	Mutant
TCGA.S9.A6WP	42	Male	2.682011391	3.699651569	diploid	Oligoastrocytoma	III	Anaplastic oligodendroglioma, IDH-mutant	Codel	PN	558	18.6	0	Mutant	codel	Methylated	No combined CNA	WT
TCGA.S9.A6WQ	57	Female	3.922026542	3.387279815	diploid	Oligoastrocytoma	II	Diffuse astrocytoma, IDH-mutant	G-CIMP-high	PN	423	14.1	0	Mutant	non-codel	Methylated	No combined CNA	Mutant
TCGA.S9.A7IQ	45	Female	1.964842186	6.481444263	Gain	Oligoastrocytoma	II	Oligodendroglioma, IDH-mutant and 1p/19	Codel	NE	1083	36.1	0	Mutant	codel	Methylated	No combined CNA	WT
TCGA.S9.A7IS	33	Female	3.079241559	3.31605883	diploid	Astrocytoma	III	Anaplastic astrocytoma, IDH-mutant	G-CIMP-low	PN	237	7.9	1	Mutant	non-codel	Methylated	No combined CNA	Mutant
TCGA.S9.A7IX	57	Male	3.525079124	4.980500635	Gain	Astrocytoma	III	Anaplastic astrocytoma, IDH-wildtype	Mesenchymal-like	CL	807	26.9	1	WT	non-codel	Unmethylated	Gain chr 7 & loss chr 10	WT
TCGA.S9.A7IY	39	Male	3.492840203	3.192762292	diploid	Oligoastrocytoma	III	Anaplastic oligodendroglioma, IDH-mutant	Codel		705	23.5	0	Mutant	codel	Methylated	No combined CNA	WT
TCGA.S9.A7IZ	48	Female	2.71918344	4.717484508	Gain	Astrocytoma	III	Anaplastic astrocytoma, IDH-mutant	G-CIMP-high	NE	600	20	0	Mutant	non-codel	Methylated	No combined CNA	Mutant
TCGA.S9.A7J0	30	Female	3.287649592	4.121098312	Gain	Oligodendroglioma	III	Anaplastic oligodendroglioma, NOS	G-CIMP-high	PN	600	20	0	Mutant	non-codel	Methylated	No combined CNA	WT
TCGA.S9.A7J1	43	Male	2.350355799	4.242488183	diploid	Oligodendroglioma	II	Oligodendroglioma, IDH-mutant and 1p/19	Codel	PN	246	8.2	0	Mutant	codel	Methylated	No combined CNA	WT
TCGA.S9.A7J2	25	Male	1.974125448	3.223839543	diploid	Oligodendroglioma	III	Anaplastic oligodendroglioma, IDH-mutant	Codel	PN	60	2	0	Mutant	codel	Methylated	No combined CNA	WT
TCGA.S9.A7J3	52	Female	2.083962184	3.47851887	diploid	Oligodendroglioma	III	Anaplastic oligodendroglioma, IDH-mutant	Codel	PN	621	20.7	0	Mutant	codel	Methylated	No combined CNA	WT
TCGA.S9.A7QW	54	Female	0.843903469	4.923729185	Gain	Astrocytoma	III	Anaplastic astrocytoma, IDH-mutant	G-CIMP-high	PN	453	15.1	0	Mutant	non-codel	Methylated	No combined CNA	Mutant
TCGA.S9.A7QX	36	Female	2.351741389	3.083979198	diploid	Astrocytoma	III	Anaplastic astrocytoma, IDH-mutant	G-CIMP-high	PN	555	18.5	0	Mutant	non-codel	Methylated	No combined CNA	Mutant
TCGA.S9.A7QY	35	Female	1.945944742	4.47095328	Gain	Oligoastrocytoma	II	Oligodendroglioma, IDH-mutant and 1p/19	Codel	PN	852	28.4	0	Mutant	codel	Methylated	No combined CNA	WT
TCGA.S9.A7QZ	41	Male	1.298365055	4.76037	diploid	Oligodendroglioma	II	Oligodendroglioma, IDH-mutant and 1p/19	Codel	PN	816	27.2	0	Mutant	codel	Methylated	No combined CNA	WT
TCGA.S9.A7R1	35	Male	2.370303799	4.549133143	Gain	Oligodendroglioma	II	Oligodendroglioma, IDH-mutant and 1p/19	Codel	NE	5094	169.8	1	Mutant	codel	Methylated	No combined CNA	WT
TCGA.S9.A7R2	69	Male	5.166012402	9.105246707	Gain	Astrocytoma	III	Anaplastic astrocytoma, IDH-wildtype	Mesenchymal-like		312	10.4	1	WT	non-codel	Unmethylated	Gain chr 7 & loss chr 10	WT
TCGA.S9.A7R3	28	Female	2.560910561	3.471876771	diploid	Astrocytoma	II	Diffuse astrocytoma, IDH-mutant	G-CIMP-high		2973	99.1	0	Mutant	non-codel	Methylated	No combined CNA	WT
TCGA.S9.A7R4	46	Male	2.229710962	2.900219563	diploid	Astrocytoma	III	Anaplastic astrocytoma, IDH-mutant	G-CIMP-high	PN	903	30.1	0	Mutant	non-codel	Methylated	No combined CNA	WT
TCGA.S9.A7R7	27	Male	4.322613212	3.079685307	diploid	Astrocytoma	II	Diffuse astrocytoma, IDH-mutant	G-CIMP-high	ME	2958	98.6	0	Mutant	non-codel	Methylated	No combined CNA	Mutant
TCGA.S9.A7R8	44	Female	4.006962211	3.725381256	diploid	Astrocytoma	III	Anaplastic astrocytoma, IDH-mutant	G-CIMP-high	PN	948	31.6	1	Mutant	non-codel	Methylated	No combined CNA	Mutant
TCGA.S9.A89V	70	Male	2.605115002	3.613896995	diploid	Astrocytoma	III	Anaplastic astrocytoma, IDH-wildtype	PA-like		561	18.7	0	WT	non-codel	Methylated	No combined CNA	Mutant
TCGA.S9.A89Z	40	Male	4.147705583	3.504836463	diploid	Astrocytoma	III	Anaplastic astrocytoma, IDH-mutant	G-CIMP-high		615	20.5	0	Mutant	non-codel	Methylated	No combined CNA	Mutant
TCGA.TM.A7C3	43	Female	2.536724343	5.775627399	Gain	Astrocytoma	III	Anaplastic astrocytoma, IDH-wildtype	Classic-like	CL	1644	54.8	1	WT	non-codel	Methylated	Gain chr 7 & loss chr 10	WT
TCGA.TM.A7C4	39	Female	2.870404177	5.497340917	Gain	Astrocytoma	II	Diffuse astrocytoma, IDH-mutant	G-CIMP-high	NE	1449	48.3	0	Mutant	non-codel	Methylated	No combined CNA	Mutant

TCGA.TM.A7C5	30	Male	1.488926268	4.556637865	diploid	Oligoastrocytoma	II	Oligodendroglioma, IDH-mutant and 1p/19	Codel	PN	1479	49.3	0	Mutant	codel	Methylated	No combined CNA	WT
TCGA.TM.A7CA	44	Male	1.522758841	4.064736299	Gain	Astrocytoma	II	Diffuse astrocytoma, IDH-mutant	G-CIMP-high	PN	1044	34.8	0	Mutant	non-codel	Methylated	No combined CNA	Mutant
TCGA.TM.A7CF	41	Female	3.996054248	5.680465018	diploid	Astrocytoma	II	Diffuse astrocytoma, IDH-mutant	G-CIMP-high	NE	1962	65.4	0	Mutant	non-codel	Methylated	No combined CNA	WT
TCGA.TM.A84B	40	Male	1.931872127	5.262662413	Gain	Astrocytoma	III	Anaplastic astrocytoma, IDH-wildtype	Mesenchymal-like		747	24.9	1	WT	non-codel	Unmethylated	Gain chr 7 & loss chr 10	WT
TCGA.TM.A84C	32	Male	2.117960953	1.814263291	diploid	Astrocytoma	II	Diffuse astrocytoma, IDH-wildtype	PA-like		486	16.2	1	WT	non-codel	Unmethylated	No combined CNA	WT
TCGA.TM.A84F	48	Male	5.853950749	5.128969712	diploid	Astrocytoma	III	Anaplastic astrocytoma, IDH-mutant	G-CIMP-high		1770	59	0	Mutant	non-codel	Methylated	No combined CNA	WT
TCGA.TM.A84G	54	Female	1.259844931	2.784252693	diploid	Oligodendroglioma	III	Anaplastic oligodendroglioma, IDH-mutant	Codel		1218	40.6	0	Mutant	codel	Methylated	No combined CNA	WT
TCGA.TM.A84H	44	Female	0.667301759	5.486534186	Gain	Oligoastrocytoma	III	Anaplastic astrocytoma, IDH-mutant	G-CIMP-high		912	30.4	0	Mutant	non-codel	Methylated	No combined CNA	Mutant
TCGA.TM.A84I	30	Male	3.299508434	3.856736756	diploid	Astrocytoma	III	Anaplastic astrocytoma, IDH-mutant	G-CIMP-low		843	28.1	0	Mutant	non-codel	Methylated	No combined CNA	Mutant
TCGA.TM.A84J	63	Male	2.842978832	7.567251092	diploid	Oligodendroglioma	III	Anaplastic oligodendroglioma, NOS	PA-like		726	24.2	0	WT	non-codel	Unmethylated	No combined CNA	WT
TCGA.TM.A84L	31	Male	3.649385529	3.661521596	diploid	Oligoastrocytoma	II	Diffuse astrocytoma, IDH-mutant	G-CIMP-high		1224	40.8	1	Mutant	non-codel	Methylated	No combined CNA	Mutant
TCGA.TM.A84M	40	Male	1.361038407	3.567679295	Gain	Oligodendroglioma	III	Anaplastic oligodendroglioma, IDH-mutant	Codel		744	24.8	0	Mutant	codel	Methylated	No combined CNA	WT
TCGA.TM.A84O	61	Female	4.296589557	3.266711594	diploid	Oligodendroglioma	III	Anaplastic oligodendroglioma, IDH-mutant	Codel		996	33.2	1	Mutant	codel	Methylated	No combined CNA	WT
TCGA.TM.A84Q	31	Male	2.963344646	3.712254599	diploid	Astrocytoma	II	Diffuse astrocytoma, IDH-mutant	G-CIMP-high		762	25.4	0	Mutant	non-codel	Methylated	No combined CNA	Mutant
TCGA.TM.A84R	46	Male	0.767400556	6.038554704	diploid	Oligodendroglioma	II	Oligodendroglioma, IDH-mutant and 1p/19	Codel		558	18.6	0	Mutant	codel	Methylated	No combined CNA	WT
TCGA.TM.A84S	36	Male	2.867975514	3.287206309	diploid	Oligodendroglioma	III	Anaplastic oligodendroglioma, IDH-mutant	Codel		447	14.9	0	Mutant	codel	Methylated	No combined CNA	WT
TCGA.TM.A84T	19	Male	3.41945799	3.142576814	diploid	Oligoastrocytoma	II	Diffuse astrocytoma, IDH-mutant	G-CIMP-high		714	23.8	0	Mutant	non-codel	Methylated	No combined CNA	Mutant
TCGA.TQ.A7RF	27	Female	3.108139687	3.306743447	diploid	Oligodendroglioma	III	Anaplastic oligodendroglioma, NOS	G-CIMP-high		642	21.4	0	Mutant	non-codel	Unmethylated	No combined CNA	WT
TCGA.TQ.A7RG	36	Male	1.303225478	2.754395833	diploid	Oligoastrocytoma	II	Oligodendroglioma, IDH-mutant and 1p/19	Codel	PN	591	19.7	0	Mutant	codel	Methylated	No combined CNA	WT
TCGA.TQ.A7RH	39	Male	3.380216879	4.828174575	Gain	Oligoastrocytoma	II	Diffuse astrocytoma, IDH-mutant	G-CIMP-high	PN	480	16	0	Mutant	non-codel	Methylated	No combined CNA	Mutant
TCGA.TQ.A7RI	37	Female	3.274723846	3.160904058	Gain	Oligodendroglioma	II	Oligodendroglioma, IDH-mutant and 1p/19	Codel	PN	60	2	0	Mutant	codel	Methylated	No combined CNA	WT
TCGA.TQ.A7RJ	25	Female	3.598377117	4.029338031	diploid	Oligoastrocytoma	II	Diffuse astrocytoma, IDH-mutant	G-CIMP-high		1212	40.4	0	Mutant	non-codel	Methylated	No combined CNA	Mutant
TCGA.TQ.A7RK	29	Male	2.525693061	3.576884882	diploid	Oligoastrocytoma	II	Diffuse astrocytoma, IDH-mutant	G-CIMP-high	PN	1323	44.1	0	Mutant	non-codel	Methylated	No combined CNA	Mutant
TCGA.TQ.A7RM	41	Female	1.341245714	3.75084229	Gain	Oligoastrocytoma	III	Anaplastic astrocytoma, IDH-mutant	G-CIMP-high	PN	1101	36.7	0	Mutant	non-codel	Methylated	Gain chr 7 & loss chr 10	WT
TCGA.TQ.A7RN	32	Male	1.051720116	4.217137636	diploid	Oligodendroglioma	II	Oligodendroglioma, IDH-mutant and 1p/19	Codel	PN	1011	33.7	0	Mutant	codel	Methylated	No combined CNA	WT
TCGA.TQ.A7RO	29	Male	2.329094886	4.489819066	diploid	Oligoastrocytoma	II	Oligodendroglioma, IDH-mutant and 1p/19	Codel	PN	936	31.2	0	Mutant	codel	Methylated	No combined CNA	WT
TCGA.TQ.A7RP	66	Male	3.21549223	3.64178877	Gain	Oligoastrocytoma	II	Diffuse astrocytoma, IDH-wildtype	Mesenchymal-like	NE	903	30.1	0	WT	non-codel	Methylated	No combined CNA	WT
TCGA.TQ.A7RQ	38	Female	0.854394678	4.396604781	diploid	Oligodendroglioma	II	Oligodendroglioma, IDH-mutant and 1p/19	Codel	PN	783	26.1	0	Mutant	codel	Methylated	No combined CNA	WT
TCGA.TQ.A7RR	38	Male	3.369954977	4.389553046	diploid	Oligoastrocytoma	II	Diffuse astrocytoma, IDH-mutant	G-CIMP-high	NE	777	25.9	0	Mutant	non-codel	Unmethylated	No combined CNA	Mutant
TCGA.TQ.A7RS	25	Female	1.25960393	3.827747904	diploid	Oligodendroglioma	II	Oligodendroglioma, IDH-mutant and 1p/19	Codel	PN	0	0	0	Mutant	codel	Methylated	No combined CNA	WT
TCGA.TQ.A7RU	51	Male	1.600079412	4.073614609	diploid	Oligodendroglioma	II	Oligodendroglioma, IDH-mutant and 1p/19	Codel	PN	1017	33.9	0	Mutant	codel	Methylated	No combined CNA	WT
TCGA.TQ.A7RV	27	Male	2.547474507	4.508105341	diploid	Astrocytoma	II	Diffuse astrocytoma, IDH-mutant	G-CIMP-high	PN	1842	61.4	0	Mutant	non-codel	Methylated	No combined CNA	Mutant
TCGA.TQ.A7RW	32	Male	3.810833802	3.698351849	diploid	Oligodendroglioma	II	Oligodendroglioma, NOS	G-CIMP-high	PN	810	27	1	Mutant	non-codel	Methylated	No combined CNA	WT
TCGA.TQ.A8XE	42	Female	2.290542363	4.925187674	diploid	Oligodendroglioma	II	Oligodendroglioma, NOS	G-CIMP-high		942	31.4	1	Mutant	non-codel	Methylated	No combined CNA	WT
TCGA.VM.A8C8	50	Female	3.221521389	3.451211112	diploid	Oligodendroglioma	II	Oligodendroglioma, NOS	G-CIMP-high		1290	43	0	Mutant	non-codel	Unmethylated	No combined CNA	Mutant
TCGA.VM.A8C9	37	Female	5.868965737	5.189875942	diploid	Astrocytoma	II	Diffuse astrocytoma, IDH-wildtype	PA-like		1296	43.2	0	WT	non-codel	Unmethylated	No combined CNA	WT
TCGA.VM.A8CA	54	Male	3.789499737	5.016835025	diploid	Oligodendroglioma	II	Oligodendroglioma, NOS	Codel		405	13.5	0	Mutant	non-codel	Methylated	No combined CNA	WT
TCGA.VM.A8CB	33	Male	1.260627908	4.4359419	diploid	Oligodendroglioma	III	Anaplastic oligodendroglioma, IDH-mutant	Codel		3	0.1	0	Mutant	codel	Methylated	No combined CNA	WT
TCGA.VM.A8CD	58	Male	4.349160013	4.03725921	diploid	Astrocytoma	III	Anaplastic astrocytoma, IDH-wildtype	Mesenchymal-like		237	7.9	1	WT	non-codel	Unmethylated	No combined CNA	WT
TCGA.VM.A8CE	25	Male	1.723427501	3.870197012	diploid	Oligodendroglioma	II	Oligodendroglioma, IDH-mutant and 1p/19	Codel		1176	39.2	0	Mutant	codel	Methylated	No combined CNA	WT
TCGA.VM.A8CF	44	Female	6.436340099	4.553102417	diploid	Astrocytoma	III	Anaplastic astrocytoma, IDH-mutant	G-CIMP-high		600	20	0	Mutant	non-codel	Methylated	No combined CNA	Mutant
TCGA.VM.A8CH	24	Female	3.5627675	3.3950628	diploid	Astrocytoma	II	Diffuse astrocytoma, IDH-mutant	G-CIMP-high		705	23.5	0	Mutant	non-codel	Unmethylated	No combined CNA	Mutant
TCGA.VV.A829	44	Male	2.894507369	2.781779346	diploid	Oligoastrocytoma	III	Anaplastic oligodendroglioma, IDH-mutant	Codel		1110	37	0	Mutant	codel	Methylated	No combined CNA	WT
TCGA.VV.A86M	36	Female	2.847275379	3.191941593	diploid	Astrocytoma	III	Anaplastic astrocytoma, IDH-mutant	G-CIMP-high		480	16	0	Mutant	non-codel	Methylated	No combined CNA	WT
TCGA.VW.A7QS	35	Female	1.279174108	3.329683339	Gain	Oligodendroglioma	III	Anaplastic oligodendroglioma, IDH-mutant	Codel		696	23.2	0	Mutant	codel	Methylated	No combined CNA	WT
TCGA.VW.A8FI	66	Male	4.707397788	4.316283342	Gain	Astrocytoma	III	Anaplastic astrocytoma, IDH-wildtype	Classic-like		243	8.1	1	WT	non-codel	Unmethylated	Gain chr 7 & loss chr 10	WT
TCGA.W9.A837	47	Male	0.677801374	4.736518289	diploid	Oligodendroglioma	II	Oligodendroglioma, IDH-mutant and 1p/19	Codel		1533	51.1	0	Mutant	codel	Methylated	No combined CNA	WT
TCGA.WH.A86K	65	Male	2.2347159	2.772751358	diploid	Astrocytoma	II	Diffuse astrocytoma, IDH-mutant	G-CIMP-high		399	13.3	0	Mutant	non-codel	Methylated	No combined CNA	WT
TCGA.WY.A858	32	Female	3.949646983	3.147420677	diploid	Astrocytoma	III	Anaplastic astrocytoma, IDH-mutant	G-CIMP-high		1320	44	0	Mutant	non-codel	Methylated	No combined CNA	Mutant
TCGA.WY.A859	34	Female	3.3492945	5.851609137	diploid	Astrocytoma	II	Diffuse astrocytoma, IDH-mutant	G-CIMP-high		1197	39.9	0	Mutant	non-codel	Methylated	No combined CNA	WT
TCGA.WY.A85A	20	Male	2.772751358	4.138544184	diploid	Astrocytoma	II	Diffuse astrocytoma, IDH-mutant	G-CIMP-high		1302	43.4	0	Mutant	non-codel	Methylated	No combined CNA	Mutant
TCGA.WY.A85B	24	Male	3.436561644	3.855581142	diploid	Astrocytoma	II	Diffuse astrocytoma, IDH-mutant	G-CIMP-high		1374	45.8	0	Mutant	non-codel	Methylated	No combined CNA	Mutant
TCGA.WY.A85C	36	Male	2.197425095	3.266861484	diploid	Astrocytoma	II	Diffuse astrocytoma, IDH-mutant	G-CIMP-high		1407	46.9	0	Mutant	non-codel	Methylated	No combined CNA	WT
TCGA.WY.A85D	60	Male	3.30287467	3.538451252	diploid	Oligoastrocytoma	II	Diffuse astrocytoma, IDH-mutant	G-CIMP-high		1131	37.7	0	Mutant	non-codel	Unmethylated	No combined CNA	Mutant
TCGA.WY.A85E	48	Female	1.9644356	4.997996871	Gain	Oligoastrocytoma	II	Diffuse astrocytoma, IDH-mutant	G-CIMP-high		624	20.8	0	Mutant	non-codel	Methylated	No combined CNA	Mutant

Table S3: Univariate and multivariable Cox regression analyses of factors associated with overall survival in glioma patients(n=610)

Variable	Univariate analysis		Multivariable analysis	
	HR (95% CI)	P	HR (95% CI)	P
Age	5.57(4.11 to 7.55)	<0.01	1.724(1.23 to 2.42)	<0.01
Gender	0.96(0.72 to 1.27)	0.78	\	\
Grade	5.01(3.97 to 6.31)	<0.01	4.67(1.23 to 2.42)	<0.01
Histology	1.91(1.64 to 2.23)	<0.01	\	\
Integrated diagnosis	1.47(1.37 to 1.57)	<0.01	0.81(0.66 to 0.98)	<0.05
Subtype	1.61(1.48 to 1.72)	<0.01	\	\
IDH.status	0.08(0.06 to 0.11)	<0.01	0.19(0.13 to 0.30)	<0.01
Chr.1p/19q.codeletion	0.23(0.15 to 0.37)	<0.01	\	\
MGMT.promoter.status	0.28(0.21 to 0.37)	<0.01	\	\
Chr.7.gain/Chr.10.loss	8.42(6.10 to 11.62)	<0.01	\	\
ATRX.status	0.45(0.33 to 0.63)	<0.01	\	\
ATG9B expression	2.34(1.75 to 3.13)	<0.01	\	\
ATG9B CNV	3.50(2.61 to 4.70)	<0.01	\	\

Table S4: the clinicopathologic information of patients whose samples were used in Figure S6.

Sample ID	Pathology ID	Diagnosis	Grade
II-1	B201614427	Astrocytoma	II
II-2	B201612108	Astrocytoma	II
II-3	B201617117	Oligodendroglioma	II
III-1	B201612262	Anaplastic astrocytoma	III
III-2	B201612197	Anaplastic oligodendroglioma	III
III-3	B201700698	Anaplastic oligoastrocytoma	III
III-4	B201610023	Anaplastic astrocytoma	III
IV-1	B201616230	Glioblastoma	IV
IV-2	B201510156	Glioblastoma	IV
IV-3	B201701054	Glioblastoma	IV
IV-4	B201612296	Glioblastoma	IV
IV-5	B201611388	Glioblastoma	IV

Table S5: the primers of indicated genes

Gene Name	Forward Primer	Reverse Primer
ASCL2	GCAGGAGAAACAGGGCCTAC	GCTGAGGGAAGTCTTGGAGC
ATG9B	CCCCTCATACAAGAAGCTCCC	TGCAGGTTGAGCCTGTGTTG
NES	CTGCTACCCTTGAGACACCTG	GGGCTCTGATCTCTGCATCTAC
PROM1	AGTCGGAAACTGGCAGATAGC	GGTAGTGTTGTACTGGGCCAAT
GAPDH	GGAGCGAGATCCCTCCAAAAT	GGCTGTTGTCATACTTCTCATGG

Table S6: Primary antibodies information list

Primary antibodies	Company	Cat.NO	Isotype	Application	Dilution Ratio
ASCL2	Millipore	#MAB4418	Mouse	CHIP	1:200
ASCL2	R&D systems	AF6539	sheep	WB/IHC/IF	1:500/1:200/
ATG7	Santa Cruz	Sc-33211	Rabbit	WB	1:200
ATG9B	Novus Biologicals	NBP1-77169	Rabbit	IHC/WB	1:200/1:1000
PROMI	CST	#86781	Rabbit	WB	1:1000
PROMI	Millipore	MAB4399-1	Mouse	IF	1:200
ACTB	Beyotime Biotechnology	AF0003	Mouse	WB	1:1000
LC3	MBL	M186-3	Mouse	WB	1:1000
LC3	MBL	M152-3	Mouse	IF	1:100
Nestin	Abcam	ab22035	Mouse	WB/IF	1:1000/1:200
P62	MBL	PM045	Rabbit	WB	1:1000
BECN1	MBL	PD017	Rabbit	WB	1:1000
Ki-67	ZSGB-BIO	ZM-0166	Rabbit	IHC	1:1

Table S7: Second antibodies information list

second antibodies	Company	Cat.NO	Application	Dilution Ratio
sheep IgG Horseradish peroxidase-conjugated Antibody	R&D systems	HAF016	WB/IHC	1:500/1:100
Anti-sheep IgG-NL637	R&D systems	NL011	IF	1:1
Anti-sheep IgG-NL493	R&D systems	NL012	IF	1:1
Anti-mouse IgG, HRP-linked Antibody	CST	#7076	WB	1：1000
Anti-rabbit IgG, HRP-linked Antibody	CST	#7074	WB	1：1000
IRDYR®800CW Goat anti-rabbit	LI-COR®	926-32211	WB	1:10000
IRDYR®800CW Goat anti-mouse	LI-COR®	926-32211	WB	1:10000
Anti-mouse IgG, Alexa Fluor 555-linked Antibody	Beyotime Biotechnology	A0460	IF	1:500
Anti-rabbit IgG, Alexa Fluor 555-linked Antibody	Beyotime Biotechnology	A0453	IF	1:500
Anti-mouse IgG, Alexa Fluor 488-linked Antibody	Beyotime Biotechnology	A0428	IF	1:500
Anti-rabbit IgG, Alexa Fluor 488-linked Antibody	Beyotime Biotechnology	A0423	IF	1:500

Table S8: the clinicopathologic information of primary glioma cells.

ID	Diagnosis	Age	Sex	IDH1/2	1p/19q codeletion
GBM1	Glioblastoma	37	Female	Wildtype	NO
GBM2	Glioblastoma	21	Male	Wildtype	NO
GBM3	Glioblastoma	68	Male	Wildtype	NO

GBM4	Glioblastoma	62	Female	Wildtype	NO
GBM5	Glioblastoma	33	Male	Wildtype	NO
GBM6	Glioblastoma	55	Male	Wildtype	NO
GBM7	Glioblastoma	36	Male	Wildtype	NO
GBM8	Glioblastoma	72	Female	Wildtype	NO

Table S9: the primer of 3 predicted binding region of ATG9B

Binding Site	Name	Forward Primer	Reverse Primer
-370—-365	pATG9B-1	AAGCCATCCTCCCACCT	AAAGAACAGCAGCCAAA
-1060—-1055	pATG9B-2	TGGTGAAACCCCATCTC	TGGCACAATCTCGGCTC
-1721—-1716	pATG9B-3	GCCTCCAGTGAACAATC	AAAGCCCATTACTGAGATA
